# Eremophilane-Type Sesquiterpenoids from Fungus *Aspergillus aurantiobrunneus*

**DOI:** 10.3390/molecules30204068

**Published:** 2025-10-13

**Authors:** Xueying Deng, Mengsha Wei, Yuyi Zheng, Yong Shen, Alan Bao, Mengru Yu, Chunmei Chen, Qin Li, Hucheng Zhu

**Affiliations:** Hubei Key Laboratory of Natural Medicinal Chemistry and Resource Evaluation, School of Pharmacy, Tongji Medical College, Huazhong University of Science and Technology, Wuhan 430030, China

**Keywords:** *Aspergillus*, eremophilane-type sesquiterpenoids, eudesmane-type sesquiterpenes

## Abstract

Six previously undescribed sesquiterpenoids, aurantiophilanes A–F (**1**–**6**), along with six identified analogues (**7**–**12**), were isolated from the fungus *Aspergillus aurantiobrunneus*. Among these, compounds **1** and **3** were identified as highly oxygenated eremophilane sesquiterpenoids, with compound **1** featuring a rare ketone functional group at C-1. The structures of all compounds were unambiguously elucidated using comprehensive spectroscopic analyses, including HRESIMS, NMR, and UV spectroscopy, supplemented by electronic circular dichroism (ECD) analyses and single-crystal X-ray diffraction. All identified compounds were evaluated for immunosuppressive activity; none showed significant effects at concentrations up to 40 µM.

## 1. Introduction

Sesquiterpenoids, biosynthesized from farnesyl pyrophosphate under the catalysis of sesquiterpene cyclases (STCs), represent one of the most structurally diverse groups of terpenoids, with over 300 distinct carbon skeletons identified to date [1,2,3,4,5,6]. Eremophilane sesquiterpenes, which consist of three isoprenes, share a characteristic decalin framework with eudesmanes and form a small but significant family of sesquiterpenoids [7,8]. Due to their structural variability, eremophilane sesquiterpenes exhibit a range of clinical therapeutic potentials including antitumor, neuroprotective [9], anti-inflammatory [10], antibacterial, cytotoxic [11], and immunosuppressive [12] activities. For example, paraconulones B showed inhibitory effects on lipopolysaccharide-induced NO production in BV2 cells [13]. These attributes highlight the importance of further research into eremophilane-type sesquiterpenoids for their promising biological properties.

**Figure 1:**
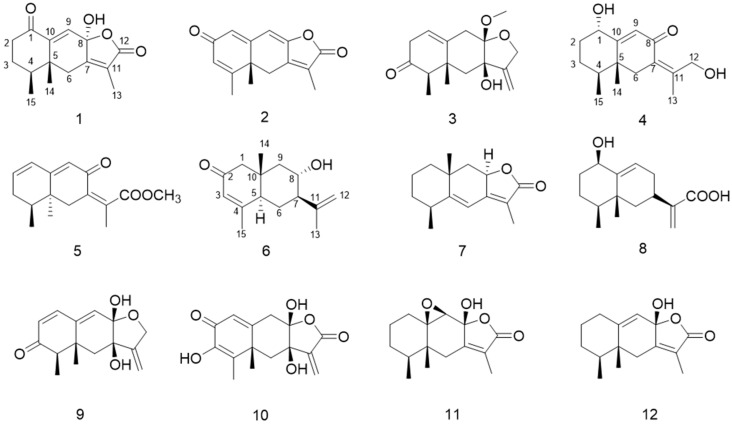
Chemical structures of compounds **1**–**12**.

The genus *Aspergillus* represents a highly significant fungal taxon, renowned for its representative secondary metabolites, among which terpenoids are particularly prominent [14]. Notably, between 2019 and 2024, sesquiterpenoids accounted for the largest proportion among the 217 new terpenoids isolated from various *Aspergillus* species [15]. Motivated by these findings, we turned our attention to less-explored members of the genus and selected *A. aurantiobrunneus* for further investigation. To date, only a single study has documented the secondary metabolites of this species, identifying sesquiterpenoids as its major chemical constituents [16]. To search for structurally unique and bioactive chemistry, six undescribed sesquiterpenoids and six known analogues were isolated from *A. aurantiobrunneus* (Figure 1). Accordingly, this study details the processes of extracting, isolating, and determining the molecular structures and assessing their biological properties.

## 2. Results and Discussion

**Figure 2:**
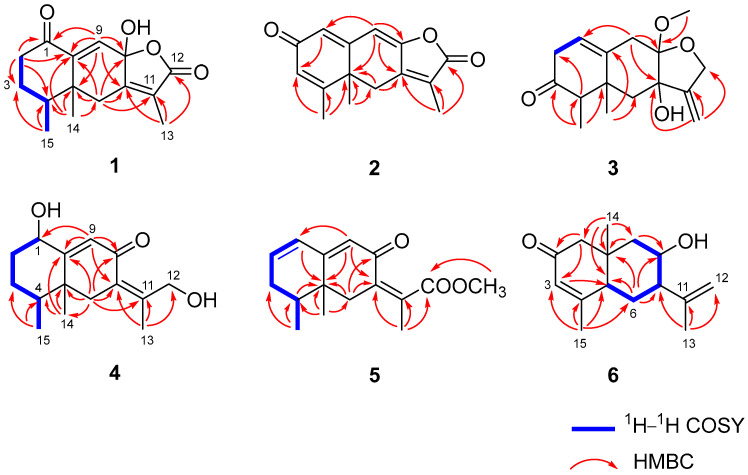
The ^1^H–^1^H COSY and key HMBC correlations of compounds **1**–**6**.

**Figure 3:**
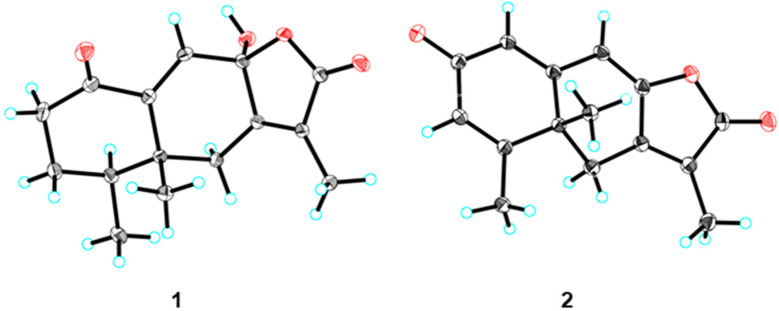
ORTEP drawings of compounds **1** and **2**.

Aurantiophilane A (**1**) was initially attained as colorless crystals. The molecular formula of C_15_H_18_O_4_ was deduced from the HRESIMS peak at *m*/*z* 285.1107 [M + Na]^+^ (calcd. for C_15_H_18_O_4_Na^+^, 285.1103), indicating seven sites of unsaturation. Analysis of the ^1^H NMR data (Table 1) and HSQC spectroscopic data of **1**, three methyl groups (*δ*_H_ 0.81, s; 1.07, d, *J* = 6.9 Hz; 1.83, s) and one olefinic proton (*δ*_H_ 6.29, s) were observed. The ^13^C NMR and DEPT data displayed 15 carbon resonances, comprising three methyls (*δ*_C_ 8.4, 15.3, and 18.4), three methylenes (*δ*_C_ 29.3, 36.7, and 41.4), one olefinic methine (*δ*_C_ 127.7), one quaternary carbon (*δ*_C_ 47.5), four nonprotonated carbons (including one oxygenated at *δ*_C_ 100.8, and three olefinic *δ*_C_ 124.7, 150.3, and 159.4), and one ester carbonyl at *δ*_C_ 173.5, along with one ketone at *δ*_C_ 204.2. One *α*, *β*-conjugated carbonyl, one double bond, and the ester carbonyl occupied four degrees, indicating the presence of the tricyclic ring skeleton. The ^1^H−^1^H COSY correlations of H_2_-2/H_2_-3/H-4/H_3_-15, along with HMBC correlations from H_2_-2 to C-1, C-3, C-4, and C-10, from H_2_-6 to C-5, C-7, C-8, C-10, and C-11, from H-9 to C-1, C-5, C-7, C-8, and C-10, and from H_3_-14 to C-4, C-5, C-6, and C-10 (Figure 2), constructed an eremophilane-type sesquiterpene framework [17]. The chemical shift of C-8 (*δ*_C_ 100.8) revealed the presence of a hemiacetal fragment. Consequently, the HMBC correlations from H_3_-13 to C-7, C-11, and C-12 confirmed the existence of *γ*-lactone. The data of single-crystal X-ray diffraction were acquired by utilizing Cu K*α* radiation, which determined the planar structure and absolute configuration of **1** as 4*S*, 5*S*, 8*R* (Figure 3).

Aurantiophilane B (**2**) was isolated as yellow crystals. Its molecular formula was determined to be C_15_H_14_O_3_, established by the [M + Na]^+^ ion peak at *m*/*z* 265.0835 (calcd. for C_15_H_14_O_3_Na^+^, *m*/*z* 265.0841) in the HRESIMS, corresponding to nine degrees of unsaturation. The ^1^H NMR data (Table 1) of **2** revealed three methyl groups (*δ*_H_ 1.37, s; 2.01, s; 2.12, s), and three olefinic protons (*δ*_H_ 6.19, s; 6.23, s; 6.30, s). The ^13^C NMR data (Table 1) displayed signals for three methyls (*δ*_C_ 9.1, 19.4, and 29.1), one methylene (*δ*_C_ 31.4), three olefinic methines (*δ*_C_ 106.9, 128.0, and 128.3), one quaternary carbon (*δ*_C_ 42.3), five olefinic nonprotonated carbons (*δ*_C_ 125.7, 144.8, 152.1, 157.9, and 162.3), and two carbonyls (an ester carbonyl at *δ*_C_ 169.9 and one ketone at *δ*_C_ 185.1). Among the 15 carbons, the existence of two carbonyls and four double bonds accounted for six degrees of unsaturation, illustrating that **2** possessed a tricyclic ring system. Comparison of the aforementioned data with the reported 2-oxo-3-hydroxy-eremophila-1(10),3,7(11),8-tetraen-8,12-olide revealed a major difference: the hydroxyl group at C-3 was absent in **2** [18], as further confirmed by HMBC correlations from H-1 to C-3 and from H_3_-15 to C-3, C-4, and C-5 (Figure 2). The absolute configuration of **2** was established as 5*R* by single-crystal X-ray diffraction (Figure 3).

**Figure 4:**
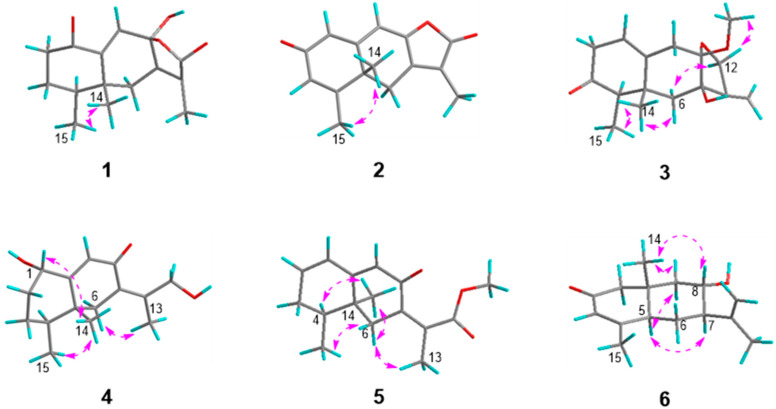
The key NOESY correlations of compounds **1**–**6**.

**Figure 5:**
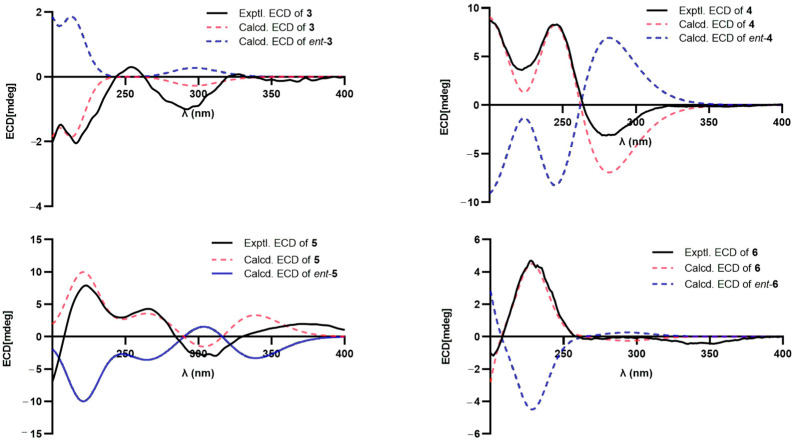
The experimental and calculated ECD curves of compounds **3**–**6**.

Aurantiophilane C (**3**), identified as colorless oil, was determined as C_16_H_22_O_4_, representing six sites of unsaturation, which was confirmed by an ion peak at *m*/*z* 301.1416 [M + Na]^+^ (calcd. for C_16_H_22_O_4_Na^+^, 301.1416) in the HRESIMS. The ^1^H and ^13^C NMR data (Table 1) of **3** were quite comparable to those of septoreremophilane D [10]. The HMBC correlation (Figure 2) from OMe-8 to C-8 indicated that the hydroxyl group at C-8 in septoreremophilane D was replaced by a methoxyl group in **3**. The NOESY correlations (Figure 4) of H_3_-14/H_3_-15, H_3_-14/H-9*β*, H-4/H-6*α* indicated that H_3_-14 and H_3_-15 were cofacial and were assigned as *β*-oriented; H_3_-14/H-6*β*, H-6*α*/H-12*α*, and H-12*β*/OCH_3_-8 indicated that OH-7 and OCH_3_-8 were on the same side and assigned as *β*-orientated. The calculated spectrum of 4*R*, 5*R*, 7*R*, 8*S*-**3** showed a close similarity with the experimental ECD spectra of **3**. Thus, the absolute configuration of **3** was established to be 4*R*, 5*R*, 7*R*, 8*S*. (Figure 5).

Aurantiophilane E (**4**), identified as yellow oil, was confirmed as C_15_H_22_O_4_ ([M + Na]^+^ *m*/*z* 273.1467 (calcd. For C_15_H_22_O_3_Na, 273.1467) by its HRESIMS spectra, which possessed five sites of unsaturation. The ^1^H and ^13^C NMR data (Table 2) indicated **4** to be an eremophilane derivative [19]. The significant difference between (4a*S*, 5*S*, 8*R*)-5,6,7,8-tetrahydro-8-hydroxy-3-(1-hydroxypropan-2-yl)-4a,5-dimethylnaph-thalen-2(4a*H*)-one and compound **4** was that the double bond Δ^6^ was replaced by Δ^7,11^ in **4**, which was supported by the HMBC correlations (Figure 2) from H_2_-6 to C-7, C-8, C-10, and C-11, from H_3_-13 to C-7, C-11, and C-12, and from H-9 to C-7. NOESY correlations (Figure 4) of H_3_-14/H_3_-15, H-1/H_3_-14 indicated that H-1, H_3_-14, and H_3_-15 were on the same side and assigned as *β*-oriented, while OH-1 was *α*-oriented. The *Z* geometry for the double bond Δ^7,11^ was evident from the NOESY correlations of H-6 and H_3_-13. The absolute configuration was assigned as 1*S*, 4*S*, 5*R*, supported by a comparison of the experimental ECD spectrum with the calculated ECD spectrum of **4** (Figure 5).

Aurantiophilane F (**5**) was isolated as colorless oil. The molecular formula of C_16_H_20_O_3_, indicating seven sites of unsaturation, was determined by an ion peak at *m*/*z* 283.1313 [M + Na]^+^ (calcd. for C_16_H_20_O_3_Na, 283.1310) in the HRESIMS spectrum. The ^1^H NMR spectroscopic data (Table 2), along with the HSQC spectrum, suggested the presence of three methyl groups (*δ*_H_ 0.98, s; 1.02, d, *J* = 6.8 Hz; 2.01, s) and three olefinic protons (*δ*_H_ 5.78, s; 6.16, d, *J* = 2.6 Hz; 6.25, m). The ^13^C NMR data (Table 2) displayed signals for three methyls (*δ*_C_ 14.7, 17.0, and 17.1), two methylenes (*δ*_C_ 32.6 and 38.1), four methines including three olefinic ones (*δ*_C_ 124.3, 128.0, and 138.8), one quaternary carbon (*δ*_C_ 38.5), three olefinic nonprotonated carbons (*δ*_C_ 132.0, 137.2, and 163.1), one ester carbonyl (*δ*_C_ 172.6), and one ketone (*δ*_C_ 187.4). One *α*, *β*-conjugated carbonyl, two double bonds, and the ester carbonyl occupied five degrees, indicating the existence of the bicyclic ring skeleton in **5**. The ^1^H−^1^H COSY correlations of H-1/H-2/H_2_-3/H-4/H_3_-15 and HMBC correlations (Figure 2) from H_3_-14 to C-4, C-5, C-6, and C-10, from H_3_-15 to C-3, C-4, and C-5, from H_2_-6 to C-5, C-7, C-8, and C-11, from H-9 to C-1, C-5, and C-7, from H_3_-13 to C-7, C-11, and C-12, and from OMe-12 to C-12 confirmed the planar structure of **5**. NOESY correlations (Figure 4) of H-4/H_3_-14, H_3_-15/H-6*β*, H-6*α*/H_3_-15, H_3_-15/H-3*β*, and H-3*β*/H-6*β* indicated the contrary orientation of H_3_-14 and H_3_-15. The *Z* geometry for the double bond Δ^7,11^ was evident from the NOESY correlations of H-6/H_3_-13. The calculated ECD curve for 4*S*, 5*R*-**5** was in good accordance with the experimental ECD spectrum, assigning the absolute configuration of **5** as 4*S*, 5*R* (Figure 5).

Aurantiophilane G (**6**) was purified as a white powder with the molecular formula of C_15_H_22_O_2_([M + Na]^+^ *m*/*z* 257.1514 (calcd. for C_15_H_22_O_2_Na, 257.1517) by HRESIMS. The ^1^H NMR data (Table 2) of **6** revealed three methyl groups (*δ*_H_ 0.94, s; 1.78, s; 1.90, s), two terminal olefinic protons (*δ*_H_ 4.97, d, *J* = 3.1 Hz; 4.93, d, *J* = 3.1 Hz), and one olefinic proton (*δ*_H_ 5.89, s). The ^13^C NMR and DEPT data (Table 2) displayed signals for three methyls (*δ*_C_ 18.1, 19.5, and 22.3), four methylenes including one olefinic (*δ*_C_ 28.1, 47.4, 55.4, and 114.0), one olefinic methine (*δ*_C_ 127.3), one quaternary carbon (*δ*_C_ 39.0), two olefinic nonprotonated carbons (*δ*_C_ 145.7 and 161.8), and one ketone (*δ*_C_ 198.8). The ^1^H−^1^H COSY correlations of H-5/H_2_-6/H-7/H-8/H_2_-9 and HMBC correlations (Figure 2) from H-1 to C-2, C-3, C-9, and C-10, from H_2_-9 to C-1, C-5, C-7, C-8, and C-10, from H_3_-13 to C-7, C-11 and C-12, from H_3_-14 to C-1, C-5, C-9, and C-10, and from H_3_-15 to C-3, C-4, C-5, and C-6 constructed an eudesmane-type sesquiterpenoid framework. The NOESY correlation (Figure 4) of H_3_-14/H-8 suggested that H_3_-14 and H-8 were on the same side and assigned as *β*-oriented; accordingly, OH-8 was *α*-oriented. The NOESY correlations of H_3_-14/H-9*β*, H-9*α*/H-5, H-5/H-7 suggested that H-5 and H-7 were *α*-oriented. Finally, the absolute configurations of **6** were deduced as 5*R*, 7*R*, 8*S*, 10*S* by comparing the calculated and experimental ECD spectra (Figure 5).

**Figure 6:**
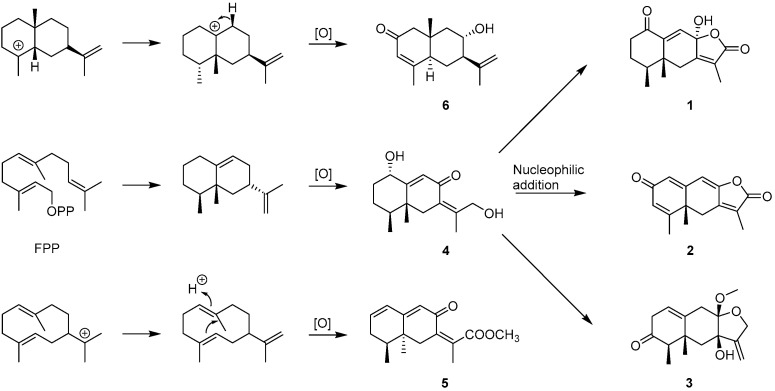
Proposed biosynthetic pathways for compounds **1**–**6**.

Six new compounds, aurantiophilanes A–H (**1**–**6**) and six known ones, neoalantolactone (**7**) [20], artefreynic acid D (**8**) [8], dihydrobipolaroxin D (**9**) [21], septoreremophilane F (**10**) [10], rel-[(4*S*, 5*R*)-9*β*, 10*β*-epoxy-8*β*-hydroxy-eremophil-12,8-olide] (**11**) [22], tsoongianus (**12**) [23], were identified as a result of chemical analysis of the fungus *Aspergillus aurantiobrunna*. Compounds **1**–**3** may be derived from **4** via nucleophilic addition and subsequent hydroxylation (Figure 6). According to the methods reported in the previous report [24], the inhibitory activities of compounds **1**–**12** against LPS-induced B lymphocyte proliferation were estimated. However, none of them exhibited any notable activity up to a concentration of 40 µM. Analyzing the experimental results, it is hypothesized that the phenomenon may be attributed to the presence of an unstable hemiacetal moiety in the molecular structure, coupled with the low bioavailability of the compounds.

## 3. Materials and Methods

### 3.1. General Experimental Procedures

A PerkinElmer PE-341 polarimeter (PerkinElmer, Waltham, MA, USA) was used to obtain optical rotations. IR spectra were acquired with a Bruker Vertex 70 FTIR spectrophotometer (Bruker, Karlsruhe, Germany). A PerkinElmer Lambda 35 spectrophotometer (PerkinElmer, Inc., Shelton, CT, USA) was employed to measure UV spectra. A JASCO-810 spectrometer was used to obtain experimental ECD spectra. A Bruker NMR spectrometer (Bruker, Germany) was employed to measure the NMR spectra. The chemical shifts for the CD_3_OD (*δ*_H_ 3.31/*δ*_C_ 49.0) and CDCl_3_ (*δ*_H_ 7.26/*δ*_C_ 77.16) signals are provided in ppm. For chromatographic separations, Sephadex LH-20 (GE Healthcare Bio-Sciences AB, Uppsala, Sweden) and silica gel (Qingdao Marine Chemical, Inc., Qingdao, China) were used. Precoated plates (200–250 µm thickness, silica gel 60 F254, Qingdao Marine Chemical, Inc.) were utilized for thin-layer chromatography analyses. Semi-preparative HPLC purifications were achieved on an H&E HPLC system utilizing a reversed-phase (RP) column (5 µm, 10 × 250 mm, Welch Ultimate XB-C18). A microTOF II instrument (Bruker, Karlsruhe, Germany) was used to obtain HRESIMS data. Graphite-monochromated Cu K*α* radiation was applied in single-crystal X-ray diffraction investigations utilizing a Bruker D8 Quest diffractometer. An X-4B microscopic melting point device (Shanghai Shenguang, Shanghai, China) was used to determine the melting points.

### 3.2. Fungal Material

The fungal strain employed in this investigation was procured from the BeNa Culture Collection (BNCC, accession no. 465.65). According to ITS sequence analysis, its sequence was 99% similar to that of *Aspergillus aurantiobrunneus*, which was deposited in the culture center of Tongji Medical College, Huazhong University of Science and Technology.

### 3.3. Fermentation, Extraction, and Isolation

The *Aspergillus aurantiobrunneus* seed culture was preserved on potato dextrose agar medium at 28 °C for 7 days. Erlenmeyer flasks (1 L) with 250.0 g of rice and 200.0 mL of distilled water that had previously been autoclaved (120 °C, 30 min) were then infected with a piece of mycelium. All flasks were kept in a foster environment at 28 °C for 45 days. Following cultivation, ethyl acetate/H_2_O (1:1) was used to extract the fermented rice, which was first extracted using 95% EtOH. The ethyl acetate-soluble fraction (380.0 g) was gained under reduced pressure. This portion was separated using column chromatography on silica gel [100–200 mesh, petroleum ether/ethyl acetate/methanol system (20:1:0 → 0:1:0 → 0:3:1, *v*:*v*:*v*)] to attain eight fractions (Fr. 1–Fr. 8).

Fr. 7 (87.7 g) was split into eight fractions (Fr. 7.1–Fr. 7.8) using an ODS column (MeOH–H_2_O 30:70 → 90:10, *v*:*v*). Fr7.1 (11.1 g) was separated into 12 fractions (Fr. 7.1.1–Fr. 7.1.12) by using silica gel CC (petroleum ether/ethyl acetate, 10:1–1:1). Sephadex LH-20 (MeOH) was employed to separate Fr. 7.1.5, yielding two fractions (Fr. 7.1.5.1–Fr. 7.1.5.2). Fr. 7.1.5.1 was purified by semi-preparative HPLC (MeOH/H_2_O, 48/52, *v*/*v*, 2.5 mL/min) separations to yield compound **11** (5.0 mg, *t*_R_ = 61.1 min) Five fractions (Fr. 7.1.8.1–Fr. 7.1.8.5) were extracted by subjecting Fr. 7.1.8 to silica gel CC (petroleum ether/dichloromethane/methanol, 2:1:0–0:0:1). Fr. 7.1.8.5 was further separated using Sephadex LH-20 (MeOH) to obtain five fractions (Fr. 7. 1.8.5.1–Fr. 7.1.8.5.5). Purification of Fr. 7.1.8.5.4 was performed by semi-preparative HPLC (MeOH/H_2_O, 29/71, *v*/*v*, 3.0 mL/min) separations to collect **10** (12.6 mg, *t*_R_ = 32.2 min). Fr. 7.2 was further purified using Sephadex LH-20 (MeOH) to obtain two fractions (Fr. 7.2.1 and Fr. 7.2.4), Fr. 7.2.2 was then submitted using silica gel column chromatography (100–200 mesh, petroleum ether/ethyl acetate, 20:1–0:1) to obtain ten fractions (Fr. 7.2.2.1–Fr. 7.2.2.10). Subsequently, Fr. 7.2.2.6 was purified by semi-preparative HPLC (MeCN/H_2_O, 26/74, *v*/*v*, 3.0 mL/min) separations to yield **1** (22.5 mg, *t*_R_ = 47.8 min) and **4** (24.1 mg, *t*_R_ = 57.6 min). Fr7.3 (2.3 g) was split into 12 fractions (Fr. 7.3.1–Fr. 7.3.12) by subjecting it to silica gel CC (petroleum ether/ethyl acetate, 15:1–1:1). Fr. 7.3.2 was further purified by using semi-preparative HPLC (MeCN/H_2_O, 28/72, *v*/*v*, 3.0 mL/min) to yield **3** (12.5 mg, *t*_R_ = 30.0 min). Then, **9** (1.5 mg, *t*_R_ = 25.4 min) was purified from Fr. 7.3.5 using semi-preparative HPLC (MeCN/H_2_O, 32/68, v/v, 3.0 mL/min). Fr. 7.3.7 was purified to afford **2** (11.8 mg, *t*_R_ = 85.7 min) and **6** (8.8 mg, *t*_R_ =70.8) using semi-preparative HPLC (MeOH/H_2_O, 48/52, *v*/*v*, 3.0 mL/min). Fr. 7.6 was separated to afford four fractions (Fr. 7.6.1–Fr. 7.6.4) using silica gel column chromatography (100–200 mesh, petroleum ether/dichloromethane, 5:1–0:1). Then, **5** (2.6 mg, *t*_R_ = 36.0 min) was purified from Fr. 7.6.1 using semi-preparative HPLC (MeCN/H_2_O, 47/53, *v*/*v*, 3.0 mL/min). Fr7.5 (11.2 g) was split into four fractions (Fr. 7.5.1–Fr. 7.5.4) by using silica gel CC (petroleum ether/ethyl acetate, 20:1–1:1). Fr. 7.5.3 was separated to obtain three fractions (Fr. 7.5.3.1–Fr. 7.5.3.3) by using silica gel column chromatography (100–200 mesh, petroleum ether/dichloromethane, 5:1–0:1). Then, **8** (12.4 mg, *t*_R_ = 37.5 min) was purified from Fr. 7.5.3.3 using semi-preparative HPLC (MeCN/H_2_O, 55/45, *v*/*v*, 3.0 mL/min). Fr. 7.8 (4.5 g) was separated using silica gel column chromatography (100–200 mesh, petroleum ether/dichloromethane, 10:1–0:1) to obtain three fractions (Fr. 7.8.1–Fr. 7.8.2). Compounds **7** (4.6 mg, *t*_R_ = 81.5 min) and **12** (4.1 mg, *t*_R_ = 42.8 min) were further purified from Fr. 7.8.1 using semi-preparative HPLC (MeCN/H_2_O, 49/51, *v*/*v*, 3.5 mL/min).

#### 3.3.1. Aurantiophilane A

Colorless crystal; m.p. 185.1–187.0 °C; [*α*${]}_{D}^{20}$ −158 (*c* 0.1, MeOH); IR *v*_max_ = 3396, 2924, and 1767 cm^−1^; UV (CH_3_CN) *λ*_max_ (log *ε*) = 223 (4.13) nm; ECD (CH_3_CN) *λ*_max_ (Δ*ε*) 218 (+11.56), and 242 (−11.64) nm. For ^1^H (400 MHz) and ^13^C NMR (100 MHz) data in CD_3_OD, see Table 1; (+)-HRESIMS [M + Na]^+^ *m*/*z* 285.1107 (calcd. for C_15_H_18_O_4_Na, 285.1103).

#### 3.3.2. Aurantiophilane B

Yellow crystal; m.p. 193.5–195.0 °C; [*α*${]}_{D}^{20}$ −447 (*c* 0.1, MeOH); IR *v*_max_ = 3420, 1775, and 1655 cm^−1^; UV (CH_3_CN) *λ*_max_ (log *ε*) = 213 (4.00), 263 (3.85), and 336 (4.20) nm; ECD (CH_3_CN) *λ*_max_ (Δ*ε*) 219 (+2.54), 245 (−2.97), 321 (+1.71), and 382 (−5.95) nm. For ^1^H (400 MHz) and ^13^C NMR (100 MHz) data in CDCl_3_, see Table 1; (+)-HRESIMS [M + Na]^+^ *m*/*z* 265.0835 (calcd. for C_15_H_14_O_3_Na, 265.0841).

#### 3.3.3. Aurantiophilane C

Colorless oil; [*α*${]}_{D}^{20}$ −135 (*c* 0.1, MeOH); IR *v*_max_ = 3433, 2922, and 1715 cm^−1^; UV (CH_3_CN) *λ*_max_ (log *ε*) = 191 (4.05) nm; ECD (CH_3_CN) *λ*_max_ (Δ*ε*) 216 (−2.1), 254 (+0.3), and 357 (−1.7) nm. For ^1^H (400 MHz) and ^13^C NMR (100 MHz) data in CDCl_3_, see Table 1; (+)-HRESIMS [M + Na]^+^ *m*/*z* 301.1416 (calcd. for C_16_H_22_O_4_Na, 301.1416).

#### 3.3.4. Aurantiophilane D

Yellow oil; [*α*${]}_{D}^{20}$ +279 (*c* 0.1, MeOH); IR v_max_ = 3422, 2923 and 1658 cm^−1^; UV (CH_3_CN) *λ*_max_ (log *ε*) = 191 (4.06) and 245 (3.94) nm; ECD (CH_3_CN) *λ*_max_ (Δ*ε*) 199 (+8.8), 246 (+8.3), and 280 (−3.1) nm. For ^1^H (400 MHz) and ^13^C NMR (100 MHz) data in CD_3_OD, see Table 2; (+)-HRESIMS [M + Na]^+^ *m*/*z* 273.1467 (calcd. for C_15_H_22_O_3_Na, 273.1467).

#### 3.3.5. Aurantiophilane E

Colorless oil; [*α*${]}_{D}^{20}$ +360 (*c* 0.1, MeOH); IR *v*_max_ = 3431, 2921,1731,1665 and 1617 cm^−1^; UV (CH_3_CN) *λ*_max_ (log ε) = 197 (3.92) and 302 (4.13) nm; ECD (CH_3_CN) *λ*_max_ (Δ*ε*) 223 (+7.9), 265 (+14.3), and 311 (−3.1) nm. For ^1^H (400 MHz) and ^13^C NMR (100 MHz) data in CDCl_3_, see Table 2; (+)-HRESIMS [M + Na]^+^ *m*/*z* 283.1313 (calcd. for C_16_H_20_O_3_ Na, 283.1310).

#### 3.3.6. Aurantiophilane F

White powder; [*α*${]}_{D}^{20}$ +291 (*c* 0.1, MeOH); IR *v*_max_ = 3419, 2920, and 1660 cm^−1^; UV (CH_3_CN) *λ*_max_ (log *ε*) = 190 (4.02) and 236 (4.05) nm; ECD (CH_3_CN) *λ*_max_ (Δ*ε*) 202 (−1.2) and 228 (+4.7) nm. For ^1^H (400 MHz) and ^13^C NMR (100 MHz) data in CDCl_3_, see Table 2; (+)-HRESIMS [M + Na]^+^ *m*/*z* 257.1514 (calcd. for C_15_H_22_O_2_Na, 257.1517).

### 3.4. X-Ray Crystallographic Analysis

Yellow crystals of **1** and **2** were obtained from MeOH at room temperature. Crystallographic data have been deposited at the Cambridge Crystallographic Data Center with the CCDC numbers 2484815 and 2484816. The data can be obtained free of charge from the CCDC, 12 Union Road, Cambridge CB1EZ, U.K. [fax: Int. +44-1223-336-033; deposit@ccdc.cam.ac.uk]

*Crystal Data of Aurantiophilane* (**1**): C_15_H_18_O_4_, *M* = 262.29, *a* = 9.0657 (16) Å, *b* = 10.0172 (18) Å, *c* = 14.548 (3) Å, *α* = 90°, *β* = 90°, *γ* =90°, *V* = 1321.1 (4) Å, *T* = 100 (2) K, space group *P*2_1_2_1_2_1_, *Z* = 4, *μ* (Cu K*α*) = 0.517 mm^−1^, 45435 reflections measured, 2698 independent reflections (*R_int_* = 0.0608). The final *R*_1_ values were 0.0290 (*I* > 2*σ* (*I*)). The final *wR* (*F*^2^) values were 0.0734 (*I* > 2*σ* (*I*)). The final *R*_1_ values were 0.0300 (all data). The final *w*R (*F*^2^) values were 0.0740 (all data). The goodness of fit on *F*^2^ was 1.061. Flack parameter = −0.21 (8).

*Crystal Data of Aurantiophilane* (**2**): C_15_H_14_O_3_, *M* = 242.26, *a*= 13.1718 (14) Å, *b* = 13.6360 (14) Å, *c* = 13.6360 (14) Å, *α* = 90°, *β* = 90°, *γ* =90°, *V* = 2449.2 (4) Å, *T* = 100 (2) K, space group *P*2_1_2_1_2_1_, *Z* = 8, *μ* (Cu K*α*) = 0.491 mm^−1^, 69507 reflections measured, 6129 independent reflections (*R_int_* = 0.0989). The final *R*_1_ values were 0.0754 (*I* > 2*σ* (*I*)). The final *wR* (*F*^2^) values were 0.1780 (*I* > 2*σ* (*I*)). The final *R*_1_ values were 0.1230 (all data). The final *w*R (*F*^2^) values were 0.2113 (all data). The goodness of fit on *F*^2^ was 0.958. Flack parameter = 0.02 (14).

### 3.5. Biological Activity

The experimental procedures and methods of immunosuppression used have been described in our previous reports [25].

## 4. Conclusions

In summary, six undescribed compound aurantiophilanes A–H (**1**–**6**) and six known ones (**7**–**12**) were first isolated from the fungus *A. aurantiobrunneus*. These findings indicated the structural diversity of sesquiterpenoids in *A. aurantiobrunneus*, which covered eremophilane-type sesquiterpenoids, eudesmane-type sesquiterpenoids, and sesquiterpenoid lactones. Despite the bioactivity assessments, none of them displayed any positive results up to the concentration of 40 µM; these novel compounds have significantly enriched the eremophilane-type sesquiterpenoid family with their diverse chemical structures.

## Figures and Tables

**Table 1:** ^1^H (400 MHz) and ^13^C (100 MHz) NMR data for compounds **1**–**3**.

	1 *^a^*		2 *^b^*		3 *^b^*	
No.	*δ*_C_, Type	*δ*_H_, (*J* in Hz)	*δ*_C_, Type	*δ*_H_, (*J* in Hz)	*δ*_C_, Type	*δ*_H_, (*J* in Hz)
1	204.2, C		128.3, CH	6.30, s	119.0, CH	5.49, dd (3.4, 3.1)
2	41.4, CH_2_	2.45, m	185.1, C		40.7, CH_2_	3.03, dd (14.0, 3.1)
		1.74, m				2.71, dd (14.0, 3.4)
3	29.3, CH_2_	1.83, m	128.0, CH	6.19, s	210.5, C	
		1.75, m				
4	42.1. CH	2.06, m	157.9, C		54.2, CH	2.55, m
5	47.5, C		42.3, C		42.8, C	
6	36.7, CH_2_	2.86, d (12.5)	31.4, CH_2_	3.04, d (16.5)	46.4, CH_2_	1.98, d (14.6)
		2.53, d (12.5)		2.53, d (16.5)		1.52, d (14.6)
7	159.4, C		144.8, C		78.4, C	
8	100.8, C		152.1, C		103.9, C	
9	127.7. CH	6.29, s	106.9, CH	6.23, s	33.8, CH_2_	2.78, d (15.2)
						2.59, d (15.2)
10	150.3, C		162.3, C		140.1, C	
11	124.7, C		125.7, C		153.5, C	
12	173.5, C		169.9, C		67.7, CH_2_	4.43, m
						4.37, m
13	8.4, CH_3_	1.83, s	9.1, CH_3_	2.01, s	104.4, CH_2_	5.22, d (2.6)
						4.94, d (2.6)
14	18.4, CH_3_	0.81, s	29.1, CH_3_	1.37, s	20.1, CH_3_	1.05, s
15	15.3, CH_3_	1.07, d (6.9)	19.4, CH_3_	2.12, s	7.3, CH_3_	1.03, d (6.6)
O-Me					48.5, CH_3_	3.30, s

*^a^ *Measured in CD_3_OD; *^b^* Measured in CDCl_3_.

**Table 2:** ^1^H (400 MHz) and ^13^C (100 MHz) NMR data for compounds **4**–**6**.

	4 *^a^*		5 *^b^*		6 *^b^*	
No.	*δ*_C_, Type	*δ*_H_, (*J* in Hz)	*δ*_C_, Type	*δ*_H_, (*J* in Hz)	*δ*_C_, Type	*δ*_H_, (*J* in Hz)
1	73.2, CH	4.51, m	128.0, CH	6.16, d (2.6)	54.4, CH_2_	2.31, m
2	34.1, CH_2_	2.21, m	138.8, CH	6.25, m	198.8, C	
		1.94, m				
3	26.3, CH_2_	2.08, m	32.6, CH_2_	2.23, m	127.3, CH	5.89, s
		1.65, m		2.22, m		
4	44.0, CH	1.77, m	37.9, CH	1.80, m	161.8, C	
5	42.6, C		38.5, C		47.4, CH	2.46, m
6	43.1, CH_2_	3.29, d (13.6)	38.1, CH_2_	2.84, d (14.2)	28.1, CH_2_	1.93, m
		2.40, d (13.6)		2.28, d (14.2)		1.48, m
7	130.9, C		132.0, C		55.4, CH	2.10, m
8	195.4, C		187.4, C		66.4, CH	3.79 dt (10.2, 4.5)
9	129.2, CH		124.3, CH	5.78, s	47.4, CH_2_	1.96, m
						1.39, m
10	169.6, C		163.1, C		39.0, C	
11	146.9, CH	4.53, m	137.2, C		145.7, C	
12	63.8, CH_2_	4.40, m	172.6, C		114.0, CH_2_	4.97, d (3.1)
						4.93, d (3.1)
13	18.4, CH_3_	2.35, s	17.0, CH_3_	2.01, s	19.5, CH_3_	1.78, s
14	18.7, CH_3_	1.43, s	17.1, CH_3_	0.98, s	18.1, CH_3_	0.94, s
15	15.9, CH_3_	1.25, d (6.8)	14.7, CH_3_	1.02, d (6.8)	22.3, CH_3_	1.90, s
O-Me			52.5, CH_3_	3.80, s		

*^a^* Measured in CD_3_OD; *^b^* Measured in CDCl_3_.

## Data Availability

The authors declare that all relevant data supporting the results of this study are available within the article and its Supplementary Materials, or from the corresponding authors upon request.

## Supplementary Materials

The following supporting information can be downloaded at: https://www.mdpi.com/article/10.3390/molecules30204068/s1. The ITS region and the colony diagram of the fungus *Aspergillus aurantiobrunneus*. Calculation details, 1D and 2D NMR spectra, HRMS, IR, and UV–vis spectra for compounds **1**–**6** (PDF) [26,27].
