# Supplementary material for: Eremophilane-Type Sesquiterpenoids from Fungus Aspergillus aurantiobrunneus
Source: Molecules. 2025 Oct 13;30(20):4068. doi: 10.3390/molecules30204068 (PMC12565795; doi:10.3390/molecules30204068)
Supplement: Supplementary file 1 [file molecules-30-04068-s001.zip › molecules-3887279-supplementary.pdf]

Supporting Information

Eremophilane-Type Sesquiterpenoids from Fungus *Aspergillus aurantiobrunneus*

Xueying Deng,† Mengsha Wei,† Yuyi Zheng,† Yong Shen, Alan Bao, Mengru Yu, Chunmei Chen*, Qin Li* and Hucheng Zhu*

Hubei Key Laboratory of Natural Medicinal Chemistry and Resource Evaluation, School of Pharmacy, Tongji Medical College, Huazhong University of Science and Technology, Wuhan 430030, People's Republic of China

---

* Corresponding author:
E-mail addresses: zhuhucheng@hust.edu.cn (H. Zhu), liqin2023@hust.edu.cn (Q. Li), chenchunmei@hust.edu.cn (C. Chen)
†X.D., M.W. and Y.Z.: These authors contributed equally.

Contents

The ITS region and the colony diagram of the fungus *Aspergillus aurantiobrunneus* 4
ECD calculation details of compounds 3−6 4
1.1 Methods...4
1.2 Results...5
Table S1. Gibbs free energies[a] and equilibrium populations[b] of low-energy conformers of **3**..5
Table S2. Cartesian coordinates for the low-energy reoptimized random research conformers of **3** at B3LYP-D3(BJ)/6-31G* level of theory in methanol. 5
Table S3. Gibbs free energies[a] and equilibrium populations[b] of low-energy conformers of **4**..9
Table S4. Cartesian coordinates for the low-energy reoptimized random research conformers of **4** at B3LYP-D3(BJ)/6-31G* level of theory in methanol. 9
Table S5. Gibbs free energies[a] and equilibrium populations[b] of low-energy conformers of **5** 19
Table S6. Cartesian coordinates for the low-energy reoptimized random research conformers of **5** at B3LYP-D3(BJ)/6-31G* level of theory in methanol. 19
Table S7. Gibbs free energies[a] and equilibrium populations[b] of low-energy conformers of **6**22
Table S8. Cartesian coordinates for the low-energy reoptimized random research conformers of **6** at B3LYP-D3(BJ)/6-31G* level of theory in methanol. 22
HRESIMS, IR, UV, 1D and 2D NMR spectra for compounds 1–6. 29
Figure S1. ^{1}H NMR spectrum of aurantiophilane A (**1**) in CD_3OD. 29
Figure S2. ^{13}C NMR spectrum of aurantiophilane A (**1**) in CD_3OD. 29
Figure S3. HSQC spectrum of aurantiophilane A (**1**) in CD3OD. 30
Figure S4. HMBC spectrum of aurantiophilane A (**1**) in CD_3OD. 30
Figure S5. ^{1}H–^{1}H COSY spectrum of aurantiophilane A (**1**) in CD_3OD. 31
Figure S6. NOESY spectrum of aurantiophilane A (**1**) in CD_3OD. 31
Figure S7. HRESIMS spectrum of aurantiophilane A (**1**) in CD_3OD. 32
Figure S8. IR spectrum of aurantiophilane A (**1**) in CD_3OD. 32
Figure S9. UV spectrum of aurantiophilane A (**1**) in MeCN. 33
Figure S10. ^{1}H NMR spectrum of aurantiophilane B (**2**) in $CDCl_3$. 33
Figure S11. ^{13}C NMR spectrum of aurantiophilane B (**2**) in $CDCl_3$. 34
Figure S13. HMBC spectrum of aurantiophilane B (**2**) in $CDCl_3$. 35
Figure S14. ^{1}H–^{1}H COSY spectrum of aurantiophilane B (**2**) in $CDCl_3$. 35
Figure S15. NOESY spectrum of aurantiophilane B (**2**) in $CDCl_3$. 36
Figure S16. HRESIMS spectrum of aurantiophilane B (**2**) in $CDCl_3$. 36
Figure S17. IR spectrum of aurantiophilane B (**2**) in $CDCl_3$. 37
Figure S18. UV spectrum of aurantiophilane B (**2**) in MeCN. 37
Figure S19. ^{1}H NMR spectrum of aurantiophilane C (**3**) in $CDCl_3$. 38
Figure S20. ^{13}C NMR spectrum of aurantiophilane C (**3**) in $CDCl_3$ 38
Figure S21. HSQC spectrum of aurantiophilane C (**3**) in $CDCl_3$. 39
Figure S22. HMBC spectrum of aurantiophilane C (**3**) in $CDCl_3$. 39
Figure S23. ^{1}H–^{1}H COSY spectrum of aurantiophilane C (**3**) in $CDCl_3$. 40
Figure S24. NOESY spectrum of aurantiophilane C (**3**) in $CDCl_3$. 40
Figure S25. HRESIMS spectrum of aurantiophilane C (**3**) in $CDCl_3$. 41
Figure S26. IR spectrum of aurantiophilane C (**3**) in $CDCl_3$. 41

Figure S27. UV spectrum of aurantiophilane C (**3**) in MeCN. 42
Figure S28. ^{1}H NMR spectrum of aurantiophilane E (**4**) in CD_3OD. 42
Figure S29. ^{13}C NMR spectrum of aurantiophilane E (**4**) in CD_3OD. 43
Figure S30. HSQC spectrum of aurantiophilane E (**4**) in CD_3OD. 43
Figure S31. HMBC spectrum of aurantiophilane E (**4**) in CD_3OD. 44
Figure S32. ^{1}H–^{1}H COSY spectrum of aurantiophilane E (**4**) in CD_3OD. 44
Figure S33. NOESY spectrum of aurantiophilane E (**4**) in CD_3OD. 45
Figure S34. HRESIMS spectrum of aurantiophilane E (**4**) in CD_3OD. 45
Figure S35. IR spectrum of aurantiophilane E (**4**) in CD_3OD. 46
Figure S36. UV spectrum of aurantiophilane E (**4**) in MeCN. 46
Figure S3710. ^{1}H NMR spectrum of aurantiophilane F (**5**) in $CDCl_3$. 47
Figure S38. ^{13}C NMR spectrum of aurantiophilane F (**5**) in $CDCl_3$. 47
Figure S39. HSQC spectrum of aurantiophilane F (**5**) in $CDCl_3$. 48
Figure S40. HMBC spectrum of aurantiophilane F (**5**) in $CDCl_3$. 48
Figure S41. ^{1}H–^{1}H COSY spectrum of aurantiophilane F (**5**) in $CDCl_3$. 49
Figure S42. NOESY spectrum of aurantiophilane F (**5**) in CDCl3. 49
Figure S43. HRESIMS spectrum of aurantiophilane F (**5**) in $CDCl_3$. 50
Figure S44. IR spectrum of aurantiophilane F (**5**) in $CDCl_3$. 50
Figure S45. UV spectrum of aurantiophilane F (**5**) in MeCN. 50
Figure S46. ^{1}H NMR spectrum of aurantiophilane G (**6**) in $CDCl_3$. 51
Figure S47. ^{13}C NMR spectrum of aurantiophilane G (**6**) in $CDCl_3$. 52
Figure S48. HSQC spectrum of aurantiophilane G (**6**) in $CDCl_3$. 52
Figure S49. HMBC spectrum of aurantiophilane G (**6**) in $CDCl_3$. 53
Figure S50. ^{1}H–^{1}H COSY spectrum of aurantiophilane G (**6**) in $CDCl_3$. 53
Figure S51. NOESY spectrum of aurantiophilane G (**6**) in $CDCl_3$. 54
Figure S52. HRESIMS spectrum of aurantiophilane G (**6**) in $CDCl_3$. 54
Figure S53. HRESIMS spectrum of aurantiophilane G (**6**) in $CDCl_3$. 55
Figure S54. IR spectrum of aurantiophilane G (**6**) in $CDCl_3$. 55
Figure S55. UV spectrum of aurantiophilane G (**6**) in MeCN. 56

The ITS region and the colony diagram of the fungus *Aspergillus aurantiobrunneus*

GTACGAGGTGCGAGAGCCCTCCGGGGCGCCCACCTCCCACCCGTGACTACCGAACCCT
GTTGCTTCGGCGGGGAGCGCGCCCAAAACCTCCCCTTTCCGGGGAGGGGGGCACGCA
AGCCGCCGGGGACCACACCGAACTTCATGCCTGAGAGTAGTGCAGTCTGAGCCCGCA
ATAGCGAAACAGTCAAAACTTTCAACAATGGATCTCTTGGTTCCGGCATCGATGAAGA
ACGCAGCGAACTGCGATAAGTAATGTGAATTGCAGAATTCAGTGAATCATCGAGTCTTT
GAACGCACATTGCGCCCCCTGGCATTCCGGGGGGCATGCCTGTCCGAGCGTCATTACT
GCCCATCAAGCCCGGCTTGTGTGTTGGGTCGCCGTCCCCGCCTCTCAGGGGACGGGCC
CGAAAGGCAGCGGCGGCACCGGGTCTGGTCCTCGAGCGTATGGGGCTTCGTCACCCG
CTCGTCTAGGGCCAGCCGGGCGCCCGCCGGCGTCTCATCAACCATTCTCTTCAGGTTG
ACCTCGGATCAGGTAGGGATACCCGCTGAACTTAAGCATATCATAAGGCGGGAGGAAA
GAGGCCCCGCCACCC

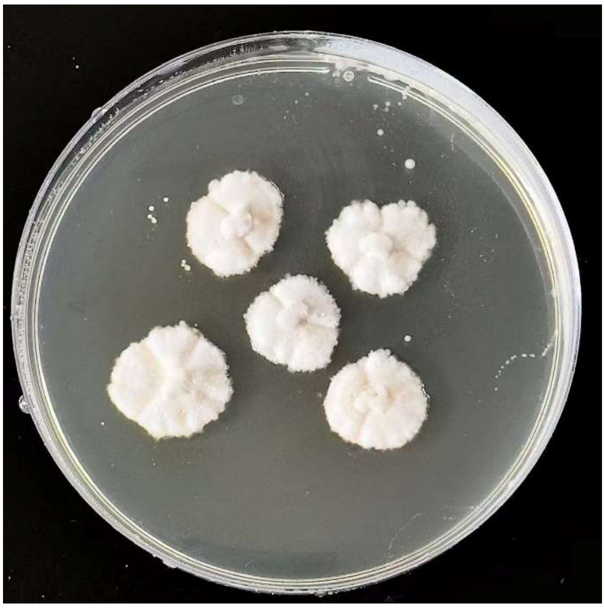

ECD calculation details of compounds 3−6

1.1 Methods

In general, conformational analyses were carried out via random searching in the Sybyl-X 2.0 using the MMFF94S force field [1]. The results showed lowest energy conformers. Subsequently, geometry optimizations and frequency analyses were implemented at the B3LYP-D3(BJ)/6-31G* level in CPCM acetonitrile using ORCA5.0.3 [2]. All conformers used for property calculations in this work were characterized to be stable point on potential energy surface (PES) with no imaginary frequencies. The excitation energies, oscillator strengths, and rotational strengths (velocity) of the first 60 excited states were calculated using the TD-DFT methodology at the PBE0/def2-TZVP level in CPCM acetonitrile using ORCA5.0.3 [2]. The ECD spectra were simulated by the overlapping Gaussian function (half the bandwidth at 1/e peak height, sigma = 0.30 for all) [3]. Gibbs free energies for conformers were determined by using thermal correction at B3LYP-D3(BJ)/6-31G* level and electronic energies evaluated at the wB97M-V/def2-TZVP level in CPCM acetonitrile using ORCA5.0.3 [2] To get the final spectra, the simulated spectra of the conformers were averaged according to the boltzmann distribution theory and their relative Gibbs free energy (ΔG). By comparing the experiment spectra with the calculated model molecules, the absolute configuration

of the compound was determined.

1.2 Results

Computational details for compound 3

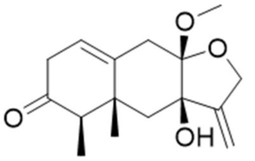

3

Table S1. Gibbs free energies[a] and equilibrium populations[b] of low-energy conformers of **3**

Conformers	ΔG(a.u.)	P(%)/100	G(a.u.)
**3**-a	0.00672	0.08	-923.514979
**3**-b	0.0	99.92	-923.521696
**3**-c	0.01119	0.0	-923.510503

[a]wB97M-V/def2-TZVP, in a.u.

[b]From ΔG values at 298.15K.

Table S2. Cartesian coordinates for the low-energy reoptimized random research conformers of **3** at B3LYP-D3(BJ)/6-31G* level of theory in methanol.

**3**-a		Standard Orientation (A.U.)			
Center number	Atomic number	Atomic Type	X	Y	Z
0	6	C	-4.211467	3.660663	-3.295824
1	6	C	-1.636656	3.382779	-4.571481
2	6	C	0.302886	1.712502	-3.23732
3	6	C	-1.029071	-0.780843	-2.373621
4	6	C	-3.160649	-0.035978	-0.567684
5	6	C	-4.608315	1.980934	-1.049886
6	6	C	0.775539	-2.667155	-1.077747
7	6	C	1.516474	-2.215215	1.693662
8	6	C	-0.759295	-1.106994	3.243382
9	6	C	-3.298123	-1.440267	1.896671
10	6	C	2.678534	1.402099	-4.835655
11	6	C	-2.161471	-2.162417	-4.683963
12	6	C	3.538189	-0.188633	2.104145

13	6	C	2.366344	1.923439	3.610041
14	8	O	-0.286637	1.500358	3.464416
15	6	C	5.932767	-0.374716	1.3509
16	8	O	2.279089	-4.597626	2.606406
17	8	O	-0.63172	-2.29824	5.628225
18	6	C	-2.287139	-1.396623	7.522557
19	8	O	-1.188843	4.471898	-6.532849
20	1	H	-5.6604	3.403023	-4.778323
21	1	H	-4.3838	5.666402	-2.739537
22	1	H	0.783784	2.73909	-1.483585
23	1	H	-6.0831	2.520589	0.296528
24	1	H	2.501129	-2.907812	-2.205568
25	1	H	-0.154171	-4.525269	-1.05843
26	1	H	-4.842321	-0.69208	3.064847
27	1	H	-3.602106	-3.485297	1.648015
28	1	H	3.329702	3.259518	-5.49528
29	1	H	4.208615	0.517663	-3.748966
30	1	H	2.313732	0.259036	-6.533425
31	1	H	-3.412399	-0.927654	-5.792435
32	1	H	-0.663322	-2.857582	-5.948349
33	1	H	-3.28261	-3.80141	-4.067339
34	1	H	2.763754	3.81846	2.851053
35	1	H	3.006409	1.870487	5.600205
36	1	H	7.322244	1.104415	1.743419
37	1	H	6.589603	-2.05132	0.337878
38	1	H	2.04038	-4.537675	4.42366
39	1	H	-4.242541	-2.062885	7.227734
40	1	H	-1.602245	-2.143397	9.334905
41	1	H	-2.282517	0.684859	7.590083

**3**-b		Standard Orientation (A.U.)			
Center number	Atomic number	Atomic Type	X	Y	Z
0	6	C	-3.157279	4.865229	-4.038493
1	6	C	-1.751666	3.10967	-5.811865
2	6	C	0.408081	1.668475	-4.581185
3	6	C	-0.614337	0.019752	-2.340796
4	6	C	-2.364536	1.597884	-0.67186
5	6	C	-3.420983	3.771259	-1.433375
6	6	C	1.694838	-0.802613	-0.741163
7	6	C	1.140326	-2.065532	1.806915
8	6	C	-0.783643	-0.499695	3.36216

9	6	C	-3.029187	0.491727	1.869232
10	6	C	1.966501	0.252038	-6.550317
11	6	C	-2.07795	-2.285345	-3.362098
12	6	C	3.438861	-1.816509	3.522763
13	6	C	3.131317	0.654955	4.913632
14	8	O	0.638083	1.529762	4.313143
15	6	C	5.261325	-3.533315	3.75731
16	8	O	0.341563	-4.558577	1.399049
17	8	O	-1.531439	-2.161056	5.331023
18	6	C	-3.074302	-1.129742	7.249676
19	8	O	-2.309252	2.892918	-8.020528
20	1	H	-5.011815	5.298871	-4.881116
21	1	H	-2.11587	6.681519	-3.980232
22	1	H	1.59383	3.139105	-3.685872
23	1	H	-4.647918	4.798422	-0.119759
24	1	H	2.844977	0.889733	-0.376979
25	1	H	2.888367	-2.129268	-1.804653
26	1	H	-4.051673	1.897855	3.005481
27	1	H	-4.30076	-1.136946	1.595894
28	1	H	2.798942	1.595161	-7.899705
29	1	H	3.513255	-0.829218	-5.683223
30	1	H	0.780295	-1.044653	-7.653993
31	1	H	-0.815238	-3.552761	-4.417574
32	1	H	-2.883347	-3.406983	-1.817934
33	1	H	-3.609074	-1.676691	-4.632322
34	1	H	4.482174	2.114528	4.292371
35	1	H	3.344593	0.408711	6.974527
36	1	H	6.851327	-3.280976	5.053343
37	1	H	5.17995	-5.274979	2.649067
38	1	H	-0.334713	-5.124575	3.007776
39	1	H	-3.07445	-2.481599	8.826248
40	1	H	-2.320179	0.695323	7.91545
41	1	H	-5.043355	-0.854772	6.612297

**3**-c		Standard Orientation (A.U.)			
Center number	Atomic number	Atomic Type	X	Y	Z
0	6	C	-2.666521	4.803924	-3.119965
1	6	C	-0.810101	3.409651	-4.815399
2	6	C	0.657417	1.263986	-3.575493
3	6	C	-1.257895	-0.592842	-2.298838
4	6	C	-2.930958	0.889946	-0.473306

5	6	C	-3.542191	3.312931	-0.874532
6	6	C	0.077634	-2.730045	-0.821937
7	6	C	0.898345	-2.152817	1.898721
8	6	C	-1.413025	-1.104264	3.519632
9	6	C	-3.71686	-0.520838	1.854562
10	6	C	2.524764	0.083752	-5.424272
11	6	C	-2.952313	-1.832751	-4.326014
12	6	C	2.86518	-0.076753	2.166076
13	6	C	1.643012	2.137656	3.466619
14	8	O	-0.54239	1.175158	4.66601
15	6	C	5.288386	-0.304787	1.504924
16	8	O	1.832945	-4.491946	2.810553
17	8	O	-2.291648	-2.736	5.375899
18	6	C	-0.751361	-3.263002	7.493315
19	8	O	-0.510732	4.01521	-7.001739
20	1	H	-4.248462	5.457613	-4.311503
21	1	H	-1.707327	6.555616	-2.491509
22	1	H	1.72764	2.156669	-2.023094
23	1	H	-4.734118	4.293165	0.503492
24	1	H	1.736694	-3.420286	-1.857735
25	1	H	-1.210885	-4.354733	-0.665393
26	1	H	-5.050984	0.56923	3.009993
27	1	H	-4.618009	-2.331956	1.365701
28	1	H	1.55199	-0.817566	-7.022981
29	1	H	3.753643	1.553505	-6.224764
30	1	H	3.729207	-1.328025	-4.493752
31	1	H	-1.860534	-3.135239	-5.525706
32	1	H	-4.472133	-2.92917	-3.424672
33	1	H	-3.839675	-0.411584	-5.557616
34	1	H	1.120666	3.619981	2.087068
35	1	H	2.880841	3.004815	4.899561
36	1	H	6.65778	1.204515	1.850024
37	1	H	5.978653	-2.021649	0.584444
38	1	H	3.002587	-4.145799	4.167132
39	1	H	0.675426	-1.777147	7.794528
40	1	H	-1.977253	-3.310817	9.174241
41	1	H	0.179213	-5.115624	7.298012

Computational details for compound 4

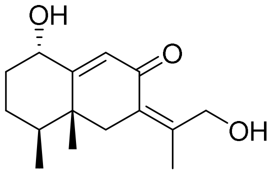

4

Table S3. Gibbs free energies[a] and equilibrium populations[b] of low-energy conformers of **4**

Conformers	ΔG(a.u.)	P(%)/100	G(a.u.)
**4**-a	0.00258	2.65	-810.189045
**4**-b	0.00208	4.49	-810.189541
**4**-c	0.00342	1.09	-810.188203
**4**-d	6e-05	38.38	-810.191567
**4**-e	0.0	40.76	-810.191624
**4**-f	0.00205	4.68	-810.189579
**4**-g	0.00205	4.63	-810.189571
**4**-h	0.00262	2.53	-810.189
**4**-i	0.00373	0.78	-810.187891

[a]wB97M-V/def2-TZVP, in a.u.

[b]From ΔG values at 298.15K.

Table S4. Cartesian coordinates for the low-energy reoptimized random research conformers of **4** at B3LYP-D3(BJ)/6-31G* level of theory in methanol.

**4**-a		Standard Orientation (A.U.)			
Center number	Atomic number	Atomic Type	X	Y	Z
0	6	C	-1.492945	4.86879	-4.688925
1	6	C	-0.139411	2.601058	-5.834902
2	6	C	1.540307	1.264289	-3.890291
3	6	C	0.048142	0.415436	-1.480647
4	6	C	-1.61849	2.570089	-0.522774
5	6	C	-3.131663	4.069144	-2.445024
6	6	C	1.981625	-0.264064	0.603603
7	6	C	0.834348	-0.668469	3.182436
8	6	C	-0.852608	1.421717	3.996811
9	6	C	-1.904388	3.025975	1.966533
10	8	O	-4.46511	6.122503	-1.397811
11	8	O	-1.484514	1.788815	6.199897
12	6	C	1.264086	-2.708793	4.687414
13	6	C	0.131339	-2.933108	7.30732
14	6	C	2.90654	-4.901547	3.944992
15	8	O	0.454954	-5.429196	8.219027
16	6	C	-1.667082	-1.887164	-2.008995

17	6	C	3.04705	-0.860913	-5.150436
18	1	H	-2.698123	5.835684	-6.081929
19	1	H	-0.08477	6.261754	-4.022005
20	1	H	-1.536479	1.26064	-6.615304
21	1	H	1.032996	3.206806	-7.446373
22	1	H	2.909975	2.690777	-3.215586
23	1	H	-4.609846	2.783643	-3.173646
24	1	H	3.075244	-1.913617	0.002176
25	1	H	3.334756	1.322922	0.731523
26	1	H	-3.187668	4.50445	2.615157
27	1	H	-3.237965	7.299431	-0.725205
28	1	H	1.062874	-1.52758	8.547883
29	1	H	-1.876503	-2.366134	7.269321
30	1	H	4.422147	-5.159531	5.350284
31	1	H	1.802694	-6.663287	4.007325
32	1	H	3.760973	-4.698076	2.071331
33	1	H	-0.258364	-5.498364	9.893321
34	1	H	-3.063392	-1.499815	-3.499032
35	1	H	-0.54353	-3.532901	-2.59896
36	1	H	-2.703891	-2.412934	-0.287044
37	1	H	4.301567	-1.851473	-3.822587
38	1	H	4.241091	-0.082541	-6.666397
39	1	H	1.792001	-2.274406	-6.020098

**4**-b		Standard Orientation (A.U.)			
Center number	Atomic number	Atomic Type	X	Y	Z
0	6	C	-1.49066	4.521612	-4.949967
1	6	C	-0.174657	2.167878	-5.957351
2	6	C	1.525899	0.954968	-3.950801
3	6	C	0.068097	0.288668	-1.464592
4	6	C	-1.577483	2.513	-0.636733
5	6	C	-3.107681	3.886801	-2.637915
6	6	C	2.031315	-0.243609	0.635284
7	6	C	0.916266	-0.471446	3.249897
8	6	C	-0.747377	1.685328	3.942524
9	6	C	-1.826514	3.143747	1.818308
10	8	O	-4.415511	6.014869	-1.716259
11	8	O	-1.333465	2.214662	6.122873
12	6	C	1.342074	-2.409665	4.882177
13	6	C	0.232552	-2.462558	7.543905
14	6	C	2.938625	-4.671605	4.247989

15	8	O	0.428494	-4.844751	8.744284
16	6	C	-1.657888	-2.041237	-1.798199
17	6	C	2.994135	-1.266154	-5.084419
18	1	H	-2.704074	5.406744	-6.389214
19	1	H	-0.060713	5.940056	-4.392018
20	1	H	-1.598188	0.790236	-6.615231
21	1	H	0.974571	2.646547	-7.626969
22	1	H	2.917206	2.412598	-3.398981
23	1	H	-4.601935	2.565098	-3.261592
24	1	H	3.128099	-1.924969	0.133707
25	1	H	3.378011	1.353537	0.638624
26	1	H	-3.094358	4.670802	2.378472
27	1	H	-3.172893	7.232972	-1.154516
28	1	H	1.10063	-0.941685	8.680657
29	1	H	-1.784832	-1.970374	7.476622
30	1	H	3.555201	-4.723884	2.27303
31	1	H	4.652044	-4.711781	5.443134
32	1	H	1.894449	-6.41364	4.688832
33	1	H	2.101762	-5.006082	9.448259
34	1	H	-0.5432	-3.731417	-2.267355
35	1	H	-2.680038	-2.428953	-0.031672
36	1	H	-3.066292	-1.767778	-3.30199
37	1	H	4.242043	-2.19269	-3.704953
38	1	H	4.191274	-0.601459	-6.651232
39	1	H	1.714881	-2.712319	-5.860378

**4**-c		Standard Orientation (A.U.)			
Center number	Atomic number	Atomic Type	X	Y	Z
0	6	C	-0.884519	4.669578	-3.709745
1	6	C	0.978782	2.616606	-4.526734
2	6	C	1.392452	0.548311	-2.524144
3	6	C	-1.201328	-0.554096	-1.616177
4	6	C	-2.764245	1.686114	-0.723926
5	6	C	-3.42052	3.621634	-2.721034
6	6	C	-0.912488	-2.460018	0.577612
7	6	C	-0.229986	-1.298743	3.087554
8	6	C	-1.961144	0.79041	3.834964
9	6	C	-3.239	2.123926	1.73962
10	8	O	-5.026577	5.573125	-1.897383
11	8	O	-2.22781	1.526039	6.016865
12	6	C	1.708767	-2.038065	4.599162

13	6	C	2.207348	-0.840801	7.175512
14	6	C	3.530617	-4.107005	3.913455
15	8	O	4.628842	-1.400875	8.165884
16	6	C	-2.585132	-1.957891	-3.76839
17	6	C	3.257369	-1.435439	-3.498133
18	1	H	-1.268613	5.998847	-5.264674
19	1	H	-0.044012	5.775567	-2.14865
20	1	H	0.318847	1.736698	-6.298036
21	1	H	2.811512	3.493519	-4.984734
22	1	H	2.238727	1.450458	-0.842309
23	1	H	-4.438519	2.705098	-4.289371
24	1	H	-2.765783	-3.397359	0.798619
25	1	H	0.409072	-3.951408	0.019739
26	1	H	-4.40303	3.725868	2.320706
27	1	H	-4.120995	6.571299	-0.662244
28	1	H	2.061589	1.22645	7.037678
29	1	H	0.680917	-1.38712	8.491823
30	1	H	3.248691	-4.859692	2.006432
31	1	H	5.483098	-3.418923	4.099717
32	1	H	3.352407	-5.703415	5.249068
33	1	H	4.566047	-3.036213	8.96746
34	1	H	-2.663537	-0.881889	-5.540329
35	1	H	-1.63679	-3.763025	-4.173888
36	1	H	-4.539322	-2.390903	-3.202633
37	1	H	2.524934	-2.417422	-5.180601
38	1	H	3.709122	-2.867324	-2.063019
39	1	H	5.048061	-0.526165	-4.043604

**4**-d		Standard Orientation (A.U.)			
Center number	Atomic number	Atomic Type	X	Y	Z
0	6	C	-2.444283	4.324658	-4.577464
1	6	C	-1.100897	2.100556	-5.817916
2	6	C	0.958502	1.010118	-4.096297
3	6	C	-0.078267	0.196577	-1.446616
4	6	C	-1.725323	2.292475	-0.346594
5	6	C	-3.63849	3.521727	-2.069191
6	6	C	2.196145	-0.215605	0.341004
7	6	C	1.456773	-0.577595	3.07608
8	6	C	-0.192507	1.491879	4.026704
9	6	C	-1.620241	2.925154	2.118897
10	8	O	-4.807842	5.557078	-0.810173

11	8	O	-0.447135	2.034738	6.279354
12	6	C	2.266318	-2.539882	4.520173
13	6	C	1.448175	-3.099905	7.225812
14	6	C	4.152139	-4.459222	3.582821
15	8	O	-1.029597	-2.564079	7.911524
16	6	C	-1.666644	-2.24956	-1.590614
17	6	C	2.428487	-1.06847	-5.471826
18	1	H	-3.924966	5.093621	-5.829103
19	1	H	-1.10098	5.870306	-4.189773
20	1	H	-2.483283	0.612822	-6.29899
21	1	H	-0.242913	2.693518	-7.620089
22	1	H	2.291061	2.571905	-3.709796
23	1	H	-5.052523	2.026183	-2.499288
24	1	H	3.301637	-1.822989	-0.345207
25	1	H	3.422195	1.468794	0.188616
26	1	H	-2.832321	4.416385	2.859183
27	1	H	-6.12657	6.207091	-1.888444
28	1	H	1.720395	-5.146565	7.52567
29	1	H	2.861058	-2.165005	8.478061
30	1	H	5.424159	-5.011641	5.138435
31	1	H	3.180522	-6.205359	2.977586
32	1	H	5.330433	-3.796541	2.012476
33	1	H	-1.173445	-0.723717	7.857535
34	1	H	-2.396514	-2.738388	0.292285
35	1	H	-3.287964	-2.050681	-2.875118
36	1	H	-0.515931	-3.845551	-2.259896
37	1	H	3.329	-0.28992	-7.178367
38	1	H	1.178763	-2.614542	-6.086636
39	1	H	3.933783	-1.891651	-4.299955

**4**-e		Standard Orientation (A.U.)			
Center number	Atomic number	Atomic Type	X	Y	Z
0	6	C	-2.434481	4.398357	-4.544099
1	6	C	-1.103972	2.158033	-5.767861
2	6	C	0.970302	1.08503	-4.053691
3	6	C	-0.052592	0.281635	-1.3976
4	6	C	-1.720824	2.360739	-0.29718
5	6	C	-3.624261	3.640155	-2.019821
6	6	C	2.226054	-0.114043	0.388444
7	6	C	1.493953	-0.507354	3.122216
8	6	C	-0.219753	1.498914	4.087098

9	6	C	-1.661336	2.929789	2.185931
10	8	O	-4.918993	5.663074	-0.872433
11	8	O	-0.515195	2.00231	6.34377
12	6	C	2.364244	-2.449926	4.559274
13	6	C	1.553468	-3.049144	7.258899
14	6	C	4.315479	-4.298878	3.617336
15	8	O	-0.954434	-2.635238	7.920285
16	6	C	-1.636561	-2.16885	-1.529273
17	6	C	2.441534	-0.994981	-5.425834
18	1	H	-3.921369	5.180633	-5.770358
19	1	H	-1.055117	5.929807	-4.197826
20	1	H	-2.494568	0.668944	-6.218473
21	1	H	-0.262377	2.727348	-7.5855
22	1	H	2.300655	2.653289	-3.682678
23	1	H	-5.099943	2.215621	-2.422057
24	1	H	3.351101	-1.703072	-0.308472
25	1	H	3.433432	1.585199	0.24564
26	1	H	-2.93205	4.35666	2.96047
27	1	H	-3.697944	6.973961	-0.506405
28	1	H	1.92289	-5.079465	7.563192
29	1	H	2.906459	-2.04432	8.522493
30	1	H	5.467873	-3.592794	2.046914
31	1	H	5.606959	-4.809556	5.171215
32	1	H	3.404823	-6.07738	3.011541
33	1	H	-1.181498	-0.803748	7.890693
34	1	H	-2.352305	-2.661834	0.35766
35	1	H	-3.267491	-1.972782	-2.801902
36	1	H	-0.485781	-3.760555	-2.208218
37	1	H	3.335303	-0.219642	-7.13729
38	1	H	1.193889	-2.54548	-6.033077
39	1	H	3.952495	-1.81164	-4.256453

**4**-f		Standard Orientation (A.U.)			
Center number	Atomic number	Atomic Type	X	Y	Z
0	6	C	0.035625	5.033126	-3.07814
1	6	C	1.7688	2.911141	-4.005326
2	6	C	1.798974	0.583146	-2.261019
3	6	C	-0.962173	-0.338278	-1.70913
4	6	C	-2.351252	1.941757	-0.657453
5	6	C	-2.665073	4.12303	-2.45027
6	6	C	-1.059526	-2.489568	0.261057

7	6	C	-0.351383	-1.681008	2.903094
8	6	C	-1.908367	0.494688	3.788332
9	6	C	-2.91321	2.179707	1.816789
10	8	O	-4.117661	6.047957	-1.322755
11	8	O	-2.30278	0.990416	6.030131
12	6	C	1.448602	-2.821356	4.33144
13	6	C	2.294762	-1.984761	6.957635
14	6	C	2.854189	-5.147673	3.481473
15	8	O	2.393404	0.591857	7.468284
16	6	C	-2.268104	-1.34214	-4.115938
17	6	C	3.542201	-1.461564	-3.326625
18	1	H	-0.062902	6.55795	-4.500184
19	1	H	0.793222	5.868098	-1.326136
20	1	H	1.203736	2.326829	-5.926108
21	1	H	3.709198	3.644317	-4.189631
22	1	H	2.569895	1.181356	-0.416585
23	1	H	-3.58396	3.455917	-4.208521
24	1	H	-3.029355	-3.182648	0.289886
25	1	H	0.096742	-4.069557	-0.406853
26	1	H	-3.890634	3.860465	2.49913
27	1	H	-4.10333	7.49557	-2.431789
28	1	H	1.094581	-3.037413	8.330416
29	1	H	4.2264	-2.720813	7.236962
30	1	H	3.026767	-6.483036	5.073748
31	1	H	1.964424	-6.159127	1.908079
32	1	H	4.80529	-4.656196	2.925544
33	1	H	0.639917	1.165091	7.482426
34	1	H	-1.477878	-3.192687	-4.638201
35	1	H	-4.303698	-1.603351	-3.776759
36	1	H	-2.051458	-0.087898	-5.754126
37	1	H	5.455336	-0.69341	-3.611878
38	1	H	2.875453	-2.161875	-5.170223
39	1	H	3.717938	-3.088826	-2.046915

**4**-g		Standard Orientation (A.U.)			
Center number	Atomic number	Atomic Type	X	Y	Z
0	6	C	-1.52361	4.837151	-4.64641
1	6	C	-0.129497	2.591697	-5.788775
2	6	C	1.571721	1.290196	-3.838729
3	6	C	0.101922	0.440804	-1.413301
4	6	C	-1.567855	2.596696	-0.464261

5	6	C	-3.133275	3.998775	-2.39561
6	6	C	2.06014	-0.194218	0.662371
7	6	C	0.937128	-0.553629	3.257095
8	6	C	-0.704393	1.584624	4.059608
9	6	C	-1.788014	3.143858	2.01263
10	8	O	-4.381344	6.045397	-1.232136
11	8	O	-1.270606	2.01134	6.267695
12	6	C	1.340454	-2.579431	4.783712
13	6	C	0.226718	-2.761565	7.437899
14	6	C	2.920118	-4.81899	4.034705
15	8	O	0.375847	-5.211623	8.502741
16	6	C	-1.579606	-1.892947	-1.9056
17	6	C	3.098149	-0.826002	-5.089997
18	1	H	-2.746647	5.754265	-6.066082
19	1	H	-0.171427	6.274952	-3.97656
20	1	H	-1.499061	1.222725	-6.569379
21	1	H	1.034528	3.216005	-7.399116
22	1	H	2.92439	2.739165	-3.180039
23	1	H	-4.538256	2.609943	-3.115778
24	1	H	3.160669	-1.847044	0.079983
25	1	H	3.404119	1.402291	0.752813
26	1	H	-3.021026	4.674677	2.628466
27	1	H	-5.455933	6.832608	-2.476983
28	1	H	1.124472	-1.32553	8.659233
29	1	H	-1.780387	-2.227452	7.400541
30	1	H	1.862359	-6.573451	4.384553
31	1	H	3.537576	-4.775201	2.05961
32	1	H	4.633513	-4.933041	5.225496
33	1	H	2.048722	-5.447327	9.18632
34	1	H	-2.983801	-1.551497	-3.399523
35	1	H	-0.434212	-3.532145	-2.472365
36	1	H	-2.60527	-2.407615	-0.17341
37	1	H	4.373279	-1.788335	-3.760955
38	1	H	4.273426	-0.046503	-6.620062
39	1	H	1.856374	-2.263821	-5.938847

**4**-h		Standard Orientation (A.U.)			
Center number	Atomic number	Atomic Type	X	Y	Z
0	6	C	-1.192494	4.612935	-3.57121
1	6	C	0.734309	2.639091	-4.440223
2	6	C	1.239783	0.537642	-2.490361

3	6	C	-1.303439	-0.683283	-1.579571
4	6	C	-2.906757	1.505112	-0.640649
5	6	C	-3.661527	3.418233	-2.621071
6	6	C	-0.91871	-2.597744	0.593129
7	6	C	-0.197328	-1.419038	3.084312
8	6	C	-2.031985	0.563345	3.903447
9	6	C	-3.300035	1.951476	1.835671
10	8	O	-5.214713	5.384364	-1.720826
11	8	O	-2.403353	1.157433	6.110047
12	6	C	1.876974	-2.025921	4.467073
13	6	C	2.508295	-0.808275	6.989711
14	6	C	3.775027	-3.994301	3.677339
15	8	O	5.150027	-0.59388	7.394487
16	6	C	-2.643402	-2.117531	-3.736987
17	6	C	3.165078	-1.359569	-3.516899
18	1	H	-1.636418	5.951608	-5.101798
19	1	H	-0.424577	5.723857	-1.984864
20	1	H	0.098907	1.774902	-6.228976
21	1	H	2.532863	3.589272	-4.886784
22	1	H	2.064987	1.434291	-0.795709
23	1	H	-4.58831	2.441728	-4.222988
24	1	H	-2.742651	-3.581922	0.851673
25	1	H	0.430927	-4.046845	-0.006396
26	1	H	-4.394935	3.593075	2.439069
27	1	H	-6.787004	4.656087	-1.144919
28	1	H	1.523653	1.00317	7.235818
29	1	H	1.78947	-2.051557	8.503775
30	1	H	3.187521	-5.102488	2.030177
31	1	H	5.617051	-3.118281	3.267227
32	1	H	4.128379	-5.304705	5.258244
33	1	H	5.759622	0.769611	6.345804
34	1	H	-4.58451	-2.61304	-3.175386
35	1	H	-2.750012	-1.037816	-5.505019
36	1	H	-1.642451	-3.892524	-4.148708
37	1	H	4.912032	-0.375385	-4.074996
38	1	H	2.448174	-2.3467	-5.203092
39	1	H	3.68867	-2.79073	-2.105902

**4**-i		Standard Orientation (A.U.)			
Center number	Atomic number	Atomic Type	X	Y	Z
0	6	C	-1.23481	4.38082	-3.880159

1	6	C	0.627905	2.320411	-4.684953
2	6	C	1.169393	0.35457	-2.609009
3	6	C	-1.357615	-0.733582	-1.511592
4	6	C	-2.900751	1.538989	-0.668266
5	6	C	-3.697997	3.314055	-2.741798
6	6	C	-0.922829	-2.494686	0.775983
7	6	C	-0.110992	-1.170268	3.16261
8	6	C	-1.818634	0.952407	3.876218
9	6	C	-3.212026	2.16166	1.780894
10	8	O	-5.247556	5.246153	-1.762294
11	8	O	-1.973912	1.802865	6.02701
12	6	C	1.922701	-1.792218	4.599653
13	6	C	2.56092	-0.422289	7.056218
14	6	C	3.726001	-3.888021	3.945151
15	8	O	5.055566	-0.871264	7.915793
16	6	C	-2.809635	-2.291714	-3.505245
17	6	C	3.00847	-1.651396	-3.586238
18	1	H	-1.69445	5.61039	-5.503344
19	1	H	-0.383209	5.588017	-2.411157
20	1	H	-0.093175	1.34595	-6.382345
21	1	H	2.422124	3.203652	-5.266414
22	1	H	2.088302	1.347522	-1.019866
23	1	H	-4.723619	2.246338	-4.221567
24	1	H	-2.750552	-3.430315	1.159521
25	1	H	0.382143	-4.008239	0.237791
26	1	H	-4.306469	3.831774	2.290705
27	1	H	-5.563114	6.45059	-3.094686
28	1	H	2.357545	1.628595	6.800889
29	1	H	1.138583	-0.914235	8.504271
30	1	H	5.677217	-3.171408	3.958546
31	1	H	3.653522	-5.388672	5.396941
32	1	H	3.33624	-4.775992	2.116769
33	1	H	5.083982	-2.455843	8.815146
34	1	H	-4.738576	-2.69434	-2.839099
35	1	H	-2.958091	-1.340605	-5.342896
36	1	H	-1.862653	-4.115369	-3.822402
37	1	H	2.206513	-2.716263	-5.184737
38	1	H	3.54798	-3.012579	-2.113246
39	1	H	4.759055	-0.749958	-4.260151

Computational details for compound 5

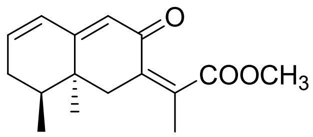

5

Table S5. Gibbs free energies[a] and equilibrium populations[b] of low-energy conformers of **5**

Conformers	ΔG(a.u.)	P(%)/100	G(a.u.)
**5**-a	0.0	79.91	-847.119098
**5**-b	0.0013	20.09	-847.117794

[a]wB97M-V/def2-TZVP, in a.u.

[b]From ΔG values at 298.15K.

Table S6. Cartesian coordinates for the low-energy reoptimized random research conformers of **5** at B3LYP-D3(BJ)/6-31G* level of theory in methanol.

**5**-a		Standard Orientation (A.U.)			
Center number	Atomic number	Atomic Type	X	Y	Z
0	6	C	-2.963007	1.090793	6.665208
1	6	C	-3.332088	-1.511959	5.589676
2	6	C	-3.714946	-1.456512	2.698276
3	6	C	-1.738047	0.239353	1.513206
4	6	C	-1.774835	2.952759	2.460028
5	6	C	-2.300454	3.08094	5.270165
6	6	C	-0.090884	-0.605196	-0.241609
7	6	C	1.840329	0.926219	-1.453708
8	6	C	1.970348	3.672632	-0.640955
9	6	C	0.760762	4.239859	1.899798
10	8	O	1.718084	5.748513	3.34058
11	6	C	3.490249	0.049792	-3.240614
12	6	C	5.385814	1.756623	-4.533761
13	6	C	-3.820013	4.50224	1.008912
14	6	C	-3.857067	-4.128146	1.608954
15	6	C	3.571348	-2.656105	-3.992702
16	8	O	2.145555	-4.360345	-3.372711
17	8	O	5.563633	-3.104909	-5.555555
18	6	C	5.826462	-5.672981	-6.367379
19	1	H	-3.253706	1.329379	8.687721
20	1	H	-4.970522	-2.427264	6.475007
21	1	H	-1.69296	-2.709975	6.044183
22	1	H	-5.555808	-0.537325	2.383856
23	1	H	-2.036412	4.926036	6.126985
24	1	H	-0.187672	-2.548696	-0.848396

25	1	H	0.982299	4.85221	-2.043852
26	1	H	3.905792	4.377454	-0.542835
27	1	H	7.288797	1.528889	-3.738734
28	1	H	5.531706	1.28882	-6.539154
29	1	H	4.867844	3.746497	-4.384363
30	1	H	-3.454716	4.508312	-1.027259
31	1	H	-5.685959	3.675268	1.330343
32	1	H	-3.850608	6.45589	1.690817
33	1	H	-5.377573	-5.185274	2.533934
34	1	H	-2.090073	-5.163089	1.912308
35	1	H	-4.235549	-4.105536	-0.424219
36	1	H	6.089043	-6.927137	-4.746499
37	1	H	4.151579	-6.283492	-7.412078
38	1	H	7.490496	-5.698609	-7.582779

**5**-b		Standard Orientation (A.U.)			
Center number	Atomic number	Atomic Type	X	Y	Z
0	6	C	-4.231715	1.677423	5.74633
1	6	C	-4.586904	-0.954185	4.736997
2	6	C	-4.412199	-1.049586	1.825453
3	6	C	-2.140009	0.46796	0.972746
4	6	C	-2.214557	3.23661	1.740249
5	6	C	-3.19873	3.546576	4.409937
6	6	C	-0.202594	-0.575777	-0.313659
7	6	C	2.038162	0.77341	-1.158149
8	6	C	2.03526	3.59918	-0.689665
9	6	C	0.423013	4.423592	1.536814
10	8	O	1.15658	6.040075	2.991162
11	6	C	4.049046	-0.259074	-2.40218
12	6	C	6.155259	1.383049	-3.428831
13	6	C	-3.925422	4.763684	-0.113357
14	6	C	-4.508562	-3.772725	0.86561
15	6	C	4.402527	-2.990229	-2.977895
16	8	O	5.854606	-3.774012	-4.58437
17	8	O	3.039483	-4.598231	-1.514096
18	6	C	3.393935	-7.227311	-2.07196
19	1	H	-4.851475	2.039687	7.674134
20	1	H	-6.425666	-1.708543	5.334551
21	1	H	-3.154907	-2.224767	5.552006
22	1	H	-6.10059	-0.063803	1.114755
23	1	H	-2.959795	5.416002	5.220057

24	1	H	-0.292853	-2.557501	-0.775564
25	1	H	1.281935	4.567379	-2.373252
26	1	H	3.930247	4.350571	-0.380842
27	1	H	7.332939	0.281596	-4.710021
28	1	H	5.422923	3.020423	-4.462439
29	1	H	7.367145	2.108744	-1.907862
30	1	H	-3.977227	6.75462	0.447597
31	1	H	-3.230816	4.634332	-2.057851
32	1	H	-5.854978	4.027469	-0.062221
33	1	H	-2.906246	-4.878026	1.572592
34	1	H	-4.481924	-3.854448	-1.201701
35	1	H	-6.244487	-4.690843	1.519578
36	1	H	2.862251	-7.639647	-4.025011
37	1	H	5.367459	-7.767186	-1.791147
38	1	H	2.169963	-8.234998	-0.755301

Computational details for compound 6

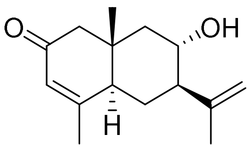

6

Table S7. Gibbs free energies[a] and equilibrium populations[b] of low-energy conformers of **6**

Conformers	ΔG(a.u.)	P(%)/100	G(a.u.)
**6**-a	0.01621	0.0	-734.948434
**6**-b	0.0	27.67	-734.964636
**6**-c	4e-05	26.61	-734.964599
**6**-d	0.01398	0.0	-734.950656
**6**-e	0.0	27.77	-734.96464
**6**-f	0.00041	17.95	-734.964228

[a]wB97M-V/def2-TZVP, in a.u.

[b]From ΔG values at 298.15K.

Table S8. Cartesian coordinates for the low-energy reoptimized random research conformers of **6** at B3LYP-D3(BJ)/6-31G* level of theory in methanol.

**6**-a		Standard Orientation (A.U.)			
Center number	Atomic number	Atomic Type	X	Y	Z
0	6	C	-17.707438	1.035771	1.597181
1	6	C	-16.893435	-1.59751	2.048736
2	6	C	-14.487193	-2.395676	1.742804
3	6	C	-12.535754	-0.548166	0.734842
4	6	C	-13.014894	2.169889	1.751706
5	6	C	-15.665819	2.931058	0.839231
6	6	C	-9.73394	-1.232284	0.924903
7	6	C	-8.181263	0.369769	-0.977199
8	6	C	-9.389156	3.017542	-1.493962
9	6	C	-10.922601	3.98236	0.760045
10	6	C	-13.728684	-5.077103	2.239281
11	6	C	-5.417341	0.507037	-0.226037
12	6	C	-3.660278	-0.750871	-1.546257
13	6	C	-4.72327	2.07981	2.046675
14	8	O	-19.907869	1.671036	1.817762
15	6	C	-12.958701	2.264491	4.662586
16	8	O	-10.797797	3.04991	-3.783957
17	1	H	-18.382635	-2.884552	2.677433
18	1	H	-12.954715	-0.505029	-1.315759
19	1	H	-16.239201	4.799237	1.552439
20	1	H	-15.67931	3.069957	-1.24512

21	1	H	-9.072414	-0.917499	2.872664
22	1	H	-9.393046	-3.23839	0.516484
23	1	H	-8.257115	-0.592035	-2.8195
24	1	H	-7.84945	4.368811	-1.852027
25	1	H	-9.572991	4.344997	2.299885
26	1	H	-11.752865	5.825286	0.266614
27	1	H	-12.234789	-5.169881	3.690016
28	1	H	-15.341941	-6.217949	2.876347
29	1	H	-12.93425	-5.961039	0.524944
30	1	H	-1.662847	-0.696888	-1.016731
31	1	H	-4.162246	-1.873816	-3.207338
32	1	H	-2.702137	1.908805	2.493777
33	1	H	-5.131496	4.098327	1.723439
34	1	H	-5.803188	1.51853	3.738077
35	1	H	-11.147617	1.56679	5.412397
36	1	H	-13.173576	4.236868	5.289846
37	1	H	-14.4798	1.154709	5.535058
38	1	H	-12.16957	1.857211	-3.654167

**6**-b		Standard Orientation (A.U.)			
Center number	Atomic number	Atomic Type	X	Y	Z
0	6	C	-17.985289	1.061449	2.178517
1	6	C	-17.063833	-1.550236	2.493531
2	6	C	-14.737133	-2.321381	1.778121
3	6	C	-12.922538	-0.489241	0.510717
4	6	C	-13.402221	2.292734	1.327733
5	6	C	-16.203781	2.884193	0.847818
6	6	C	-10.113349	-1.19982	0.679722
7	6	C	-8.476583	0.59414	-0.930484
8	6	C	-8.909758	3.358253	-0.152386
9	6	C	-11.719319	4.026714	-0.295743
10	6	C	-13.930501	-5.018913	2.111972
11	6	C	-5.708772	-0.155149	-0.921061
12	6	C	-4.708211	-1.409925	-2.878672
13	6	C	-4.168872	0.551938	1.364034
14	8	O	-20.073159	1.70723	2.899229
15	6	C	-12.827468	2.716586	4.151411
16	8	O	-7.404918	5.048818	-1.57645
17	1	H	-18.403244	-2.859635	3.366316
18	1	H	-13.404476	-0.576398	-1.528748
19	1	H	-16.705083	4.819236	1.420843
20	1	H	-16.600574	2.733517	-1.199257

21	1	H	-9.472912	-1.174526	2.661369
22	1	H	-9.808997	-3.134752	-0.009174
23	1	H	-9.164301	0.413462	-2.89808
24	1	H	-8.264702	3.6083	1.809391
25	1	H	-11.972264	6.01359	0.27214
26	1	H	-12.320982	3.892334	-2.291807
27	1	H	-12.273837	-5.160655	3.367628
28	1	H	-15.459666	-6.173177	2.911104
29	1	H	-13.356068	-5.856603	0.290355
30	1	H	-2.73187	-2.014628	-2.883114
31	1	H	-5.835511	-1.875063	-4.548955
32	1	H	-3.934786	2.619784	1.453914
33	1	H	-5.094955	-0.038965	3.135749
34	1	H	-2.280888	-0.312136	1.30366
35	1	H	-10.860365	2.272809	4.644367
36	1	H	-13.157694	4.711068	4.643701
37	1	H	-14.046849	1.555844	5.367353
38	1	H	-7.619591	4.649635	-3.346764

**6**-c		Standard Orientation (A.U.)			
Center number	Atomic number	Atomic Type	X	Y	Z
0	6	C	-18.031356	1.191584	2.038793
1	6	C	-17.277677	-1.491407	1.986152
2	6	C	-14.97911	-2.296497	1.21938
3	6	C	-13.018931	-0.417769	0.279266
4	6	C	-13.355016	2.236648	1.494026
5	6	C	-16.098606	3.073561	1.044092
6	6	C	-10.265344	-1.326154	0.402299
7	6	C	-8.467481	0.578578	-0.874775
8	6	C	-8.761933	3.220032	0.296314
9	6	C	-11.519762	4.080434	0.187905
10	6	C	-14.345212	-5.059867	1.176435
11	6	C	-5.752021	-0.343119	-0.898091
12	6	C	-4.760975	-1.379865	-2.983645
13	6	C	-4.255417	-0.059239	1.504807
14	8	O	-20.102167	1.855853	2.791111
15	6	C	-12.845956	2.196094	4.362272
16	8	O	-7.112424	5.002169	-0.823472
17	1	H	-18.720258	-2.826209	2.624466
18	1	H	-13.440914	-0.167703	-1.759666
19	1	H	-16.499967	4.929303	1.892088
20	1	H	-16.433817	3.258266	-1.011134
21	1	H	-9.688684	-1.64386	2.378596
22	1	H	-10.058323	-3.150078	-0.567872
23	1	H	-9.091125	0.735951	-2.865568
24	1	H	-8.169927	3.135766	2.28845
25	1	H	-11.671324	5.973564	1.040491
26	1	H	-12.063236	4.281256	-1.819407
27	1	H	-15.967949	-6.21712	1.756714
28	1	H	-13.754722	-5.663059	-0.73067
29	1	H	-12.747486	-5.485778	2.444389
30	1	H	-2.826717	-2.107115	-3.018995
31	1	H	-5.853713	-1.537	-4.732415
32	1	H	-5.287681	-0.811887	3.151988
33	1	H	-2.427872	-1.039143	1.383948
34	1	H	-3.889349	1.956723	1.880454
35	1	H	-14.184333	0.963473	5.363221
36	1	H	-10.933063	1.536234	4.824924
37	1	H	-13.05041	4.115029	5.139941
38	1	H	-7.290465	4.880258	-2.638147

6-d		Standard Orientation (A.U.)			
Center number	Atomic number	Atomic Type	X	Y	Z
0	6	C	-17.677247	1.139275	1.679831
1	6	C	-16.886516	-1.48433	2.212751
2	6	C	-14.496406	-2.320451	1.878029
3	6	C	-12.544483	-0.544923	0.763417
4	6	C	-12.977659	2.221675	1.661456
5	6	C	-15.638678	2.95699	0.757865
6	6	C	-9.749446	-1.25738	0.970442
7	6	C	-8.192159	0.244512	-1.008455
8	6	C	-9.442258	2.828954	-1.689064
9	6	C	-10.90571	3.964936	0.514172
10	6	C	-13.76818	-4.996014	2.453165
11	6	C	-5.433926	0.472587	-0.259136
12	6	C	-3.653832	-0.842509	-1.490776
13	6	C	-4.765363	2.193874	1.910806
14	8	O	-19.859994	1.81914	1.956606
15	6	C	-12.845627	2.474385	4.559062
16	8	O	-11.169845	2.577354	-3.738899
17	1	H	-18.375211	-2.72967	2.921947
18	1	H	-12.959405	-0.535641	-1.280739
19	1	H	-16.178705	4.873515	1.36204
20	1	H	-15.669616	2.942211	-1.328431
21	1	H	-9.054821	-0.874385	2.895133
22	1	H	-9.434853	-3.282212	0.640688
23	1	H	-8.235216	-0.854939	-2.778073
24	1	H	-7.923857	4.160646	-2.233133
25	1	H	-9.522537	4.440935	1.98845
26	1	H	-11.75024	5.763406	-0.101983
27	1	H	-13.013778	-5.947628	0.75687
28	1	H	-12.252347	-5.063945	3.882812
29	1	H	-15.385971	-6.092952	3.153413
30	1	H	-1.660234	-0.73118	-0.956326
31	1	H	-4.132916	-2.083631	-3.073254
32	1	H	-5.159403	4.186809	1.444305
33	1	H	-5.875706	1.753974	3.61742
34	1	H	-2.751967	2.045525	2.398598
35	1	H	-14.3378	1.402007	5.52423
36	1	H	-11.012873	1.824612	5.301018
37	1	H	-13.058444	4.474817	5.093949
38	1	H	-10.261703	1.933103	-5.185229

**6**-e		Standard Orientation (A.U.)			
Center number	Atomic number	Atomic Type	X	Y	Z
0	6	C	-17.98608	1.028033	2.118297
1	6	C	-17.083935	-1.589351	2.441596
2	6	C	-14.753686	-2.373735	1.752658
3	6	C	-12.913823	-0.551604	0.507437
4	6	C	-13.386088	2.232521	1.321566
5	6	C	-16.178483	2.840769	0.809241
6	6	C	-10.110948	-1.278898	0.706607
7	6	C	-8.446982	0.506336	-0.885277
8	6	C	-8.871181	3.272231	-0.108139
9	6	C	-11.674804	3.958333	-0.280749
10	6	C	-13.967075	-5.076242	2.093566
11	6	C	-5.68407	-0.259952	-0.84886
12	6	C	-4.671935	-1.519563	-2.797406
13	6	C	-4.162308	0.435961	1.45178
14	8	O	-20.07798	1.685975	2.815934
15	6	C	-12.842267	2.650494	4.15212
16	8	O	-7.340608	4.955314	-1.513315
17	1	H	-18.441006	-2.891471	3.297804
18	1	H	-13.374027	-0.63434	-1.537248
19	1	H	-16.674959	4.778622	1.376997
20	1	H	-16.551621	2.692861	-1.242451
21	1	H	-9.49165	-1.257549	2.695042
22	1	H	-9.810391	-3.215341	0.020277
23	1	H	-9.1157	0.331787	-2.859947
24	1	H	-8.24579	3.515293	1.860919
25	1	H	-11.921556	5.945927	0.287431
26	1	H	-12.255852	3.830532	-2.283374
27	1	H	-13.376912	-5.916125	0.278003
28	1	H	-12.325668	-5.22865	3.367844
29	1	H	-15.512095	-6.221874	2.87439
30	1	H	-2.699468	-2.136577	-2.782683
31	1	H	-5.785547	-1.976643	-4.479045
32	1	H	-2.278746	-0.438683	1.408297
33	1	H	-3.917372	2.502349	1.546126
34	1	H	-5.10841	-0.151591	3.214023
35	1	H	-10.882924	2.19818	4.667998
36	1	H	-13.170122	4.645711	4.643071
37	1	H	-14.080816	1.493468	5.352082
38	1	H	-7.54186	4.562589	-3.286648

**6**-f		Standard Orientation (A.U.)			
Center number	Atomic number	Atomic Type	X	Y	Z
0	6	C	-17.908925	1.44682	2.302148
1	6	C	-17.471059	-1.05296	1.148738
2	6	C	-15.248076	-1.756615	0.109193
3	6	C	-13.039572	0.078256	0.017028
4	6	C	-13.124286	2.031478	2.211705
5	6	C	-15.729114	3.313452	2.149722
6	6	C	-10.416785	-1.136183	-0.232386
7	6	C	-8.359467	0.887399	-0.622534
8	6	C	-8.408023	2.870272	1.496849
9	6	C	-11.039991	4.009826	1.764905
10	6	C	-14.941024	-4.315625	-1.073361
11	6	C	-5.779828	-0.31245	-1.036009
12	6	C	-4.14067	-0.749109	0.847511
13	6	C	-5.194794	-1.027039	-3.726519
14	8	O	-19.920659	1.996972	3.275376
15	6	C	-12.766255	0.768529	4.812377
16	8	O	-6.715426	4.874177	0.994227
17	1	H	-19.08824	-2.338864	1.188572
18	1	H	-13.320614	1.184157	-1.741777
19	1	H	-15.953802	4.704776	3.678673
20	1	H	-15.942121	4.35093	0.347545
21	1	H	-9.959344	-2.271759	1.452117
22	1	H	-10.379352	-2.447768	-1.843461
23	1	H	-8.838118	1.928121	-2.369834
24	1	H	-7.902728	1.935761	3.299691
25	1	H	-11.022759	5.396249	3.317502
26	1	H	-11.445172	5.075094	0.018734
27	1	H	-14.337464	-4.151308	-3.063225
28	1	H	-13.466475	-5.423023	-0.103066
29	1	H	-16.714032	-5.393631	-1.014787
30	1	H	-2.319803	-1.657612	0.489162
31	1	H	-4.554533	-0.250818	2.8088
32	1	H	-6.700716	-2.231706	-4.5203
33	1	H	-5.107397	0.674827	-4.929606
34	1	H	-3.391313	-2.044445	-3.895684
35	1	H	-10.960976	-0.244252	4.966409
36	1	H	-12.797759	2.218764	6.303767
37	1	H	-14.279166	-0.593637	5.224083
38	1	H	-5.058148	4.137633	0.763836

HRESIMS, IR, UV, 1D and 2D NMR spectra for compounds 1–6.

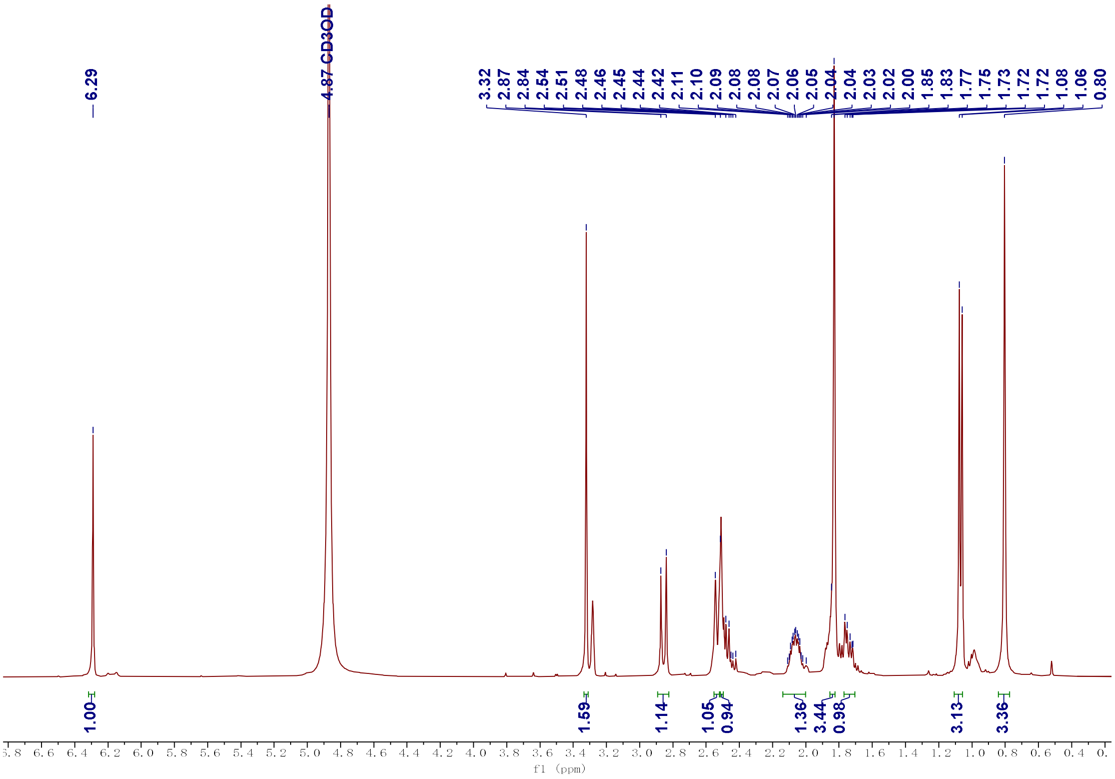

Figure S1. ^{1}H NMR spectrum of aurantiophilane A (**1**) in CD_3OD.

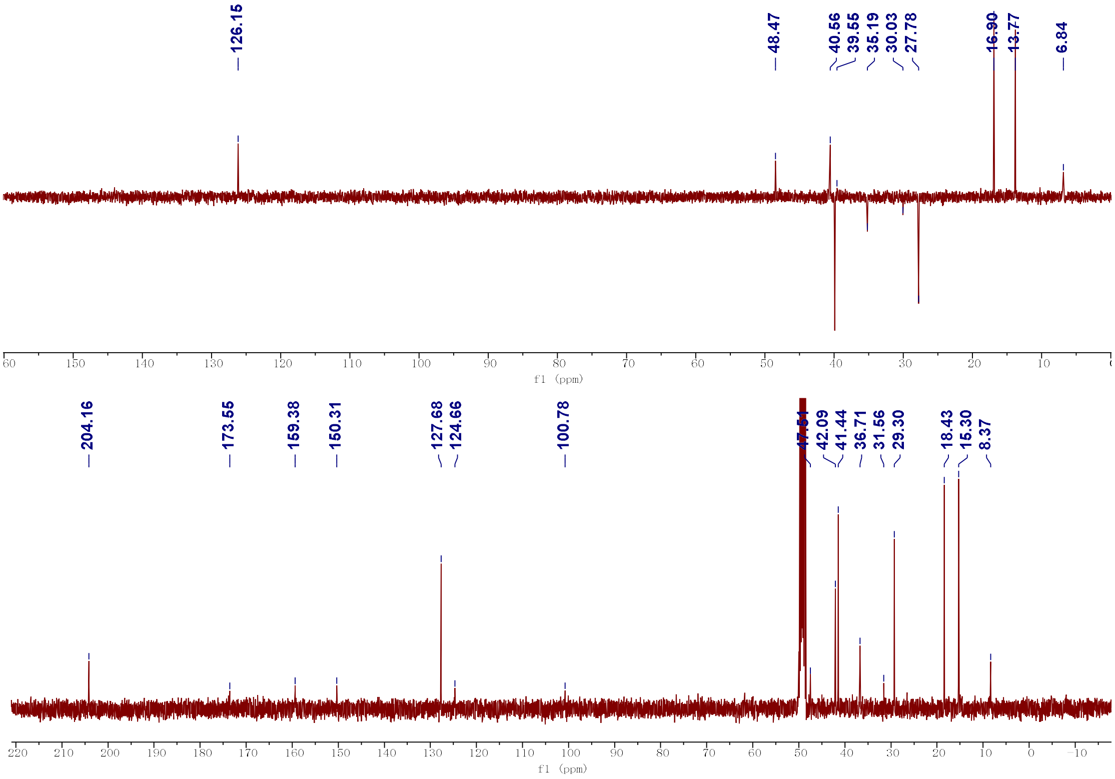

Figure S2. ^{13}C NMR spectrum of aurantiophilane A (**1**) in CD_3OD.

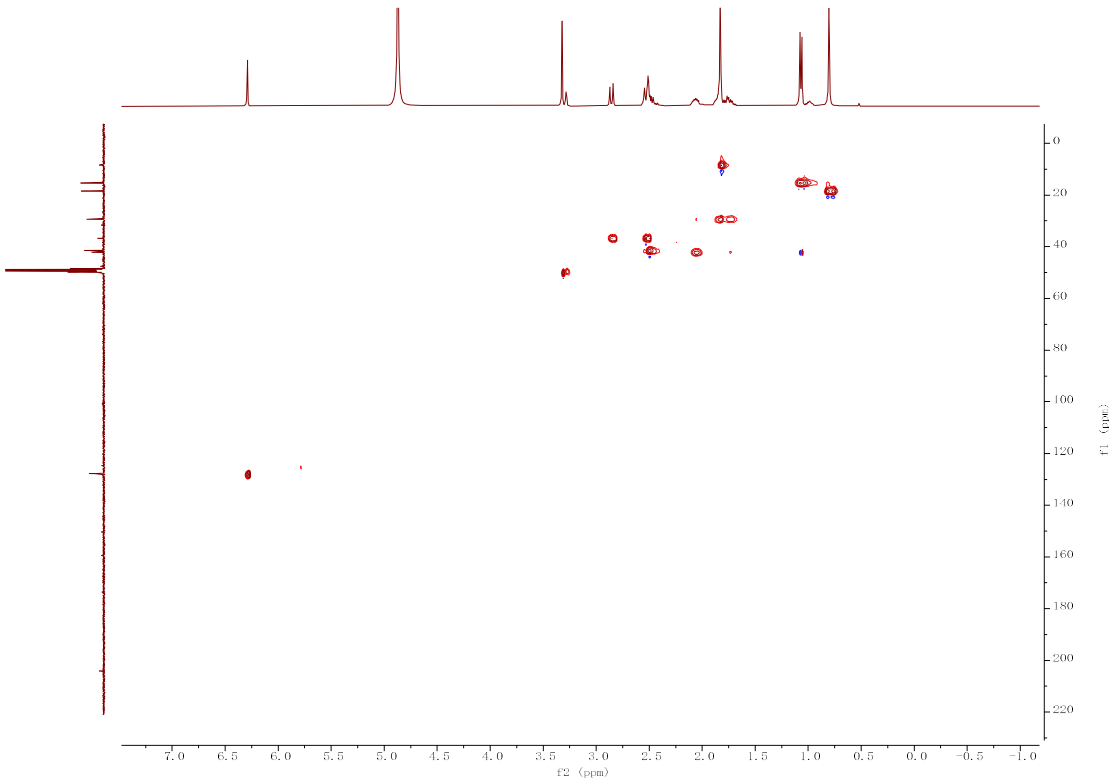

Figure S3. HSQC spectrum of aurantiophilane A (**1**) in CD3OD.

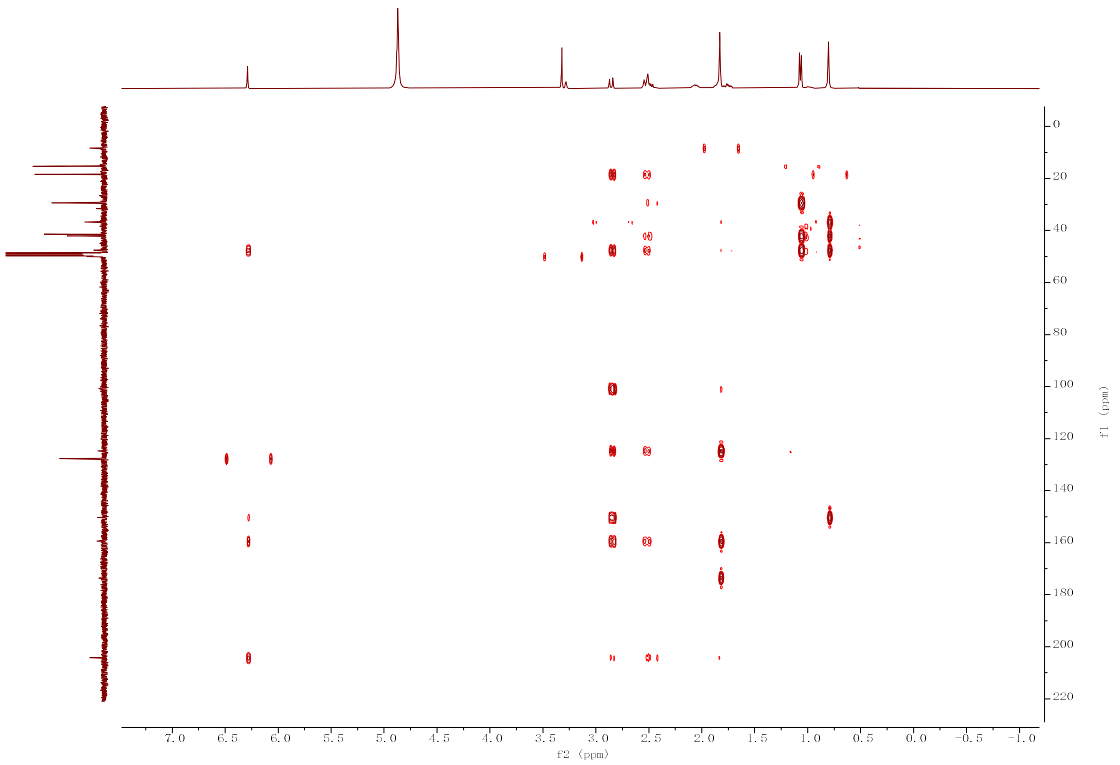

Figure S4. HMBC spectrum of aurantiophilane A (**1**) in CD_3OD.

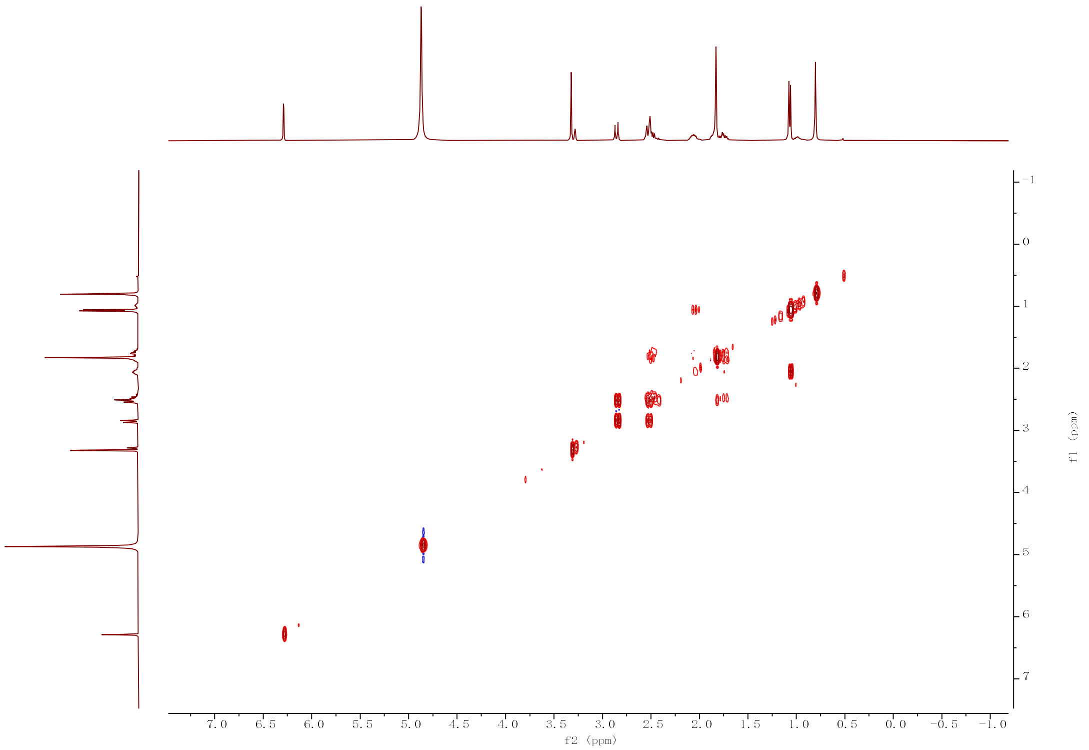

Figure S5. ^{1}H–^{1}H COSY spectrum of aurantiophilane A (**1**) in CD_3OD.

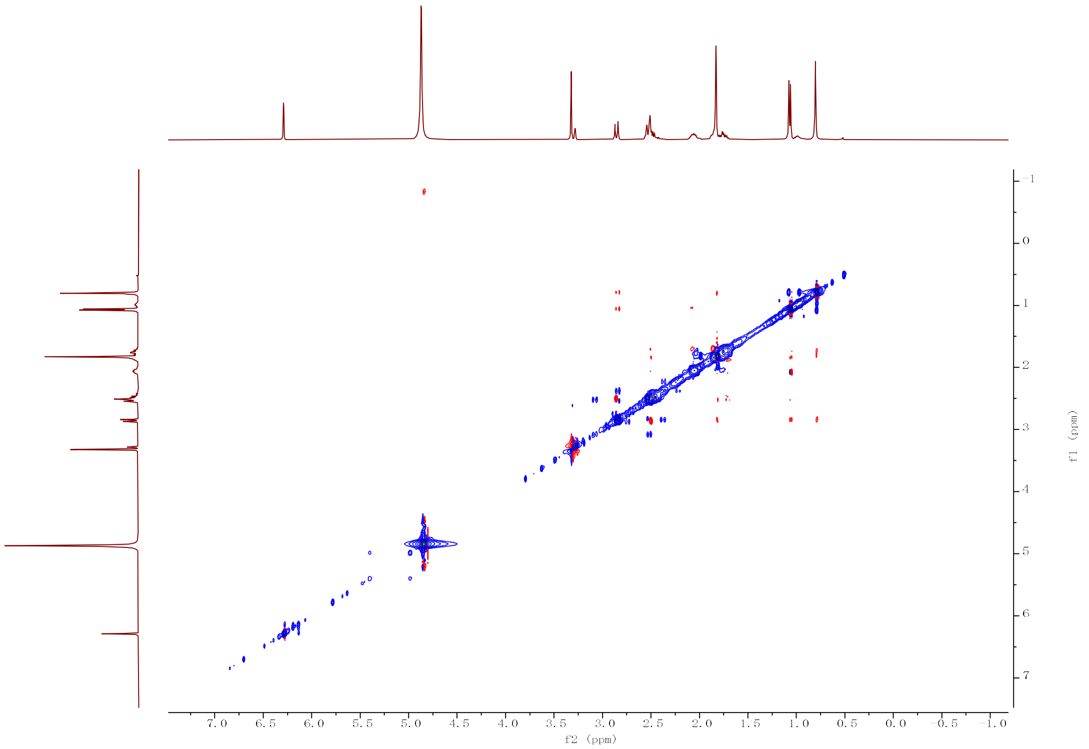

Figure S6. NOESY spectrum of aurantiophilane A (**1**) in CD_3OD.

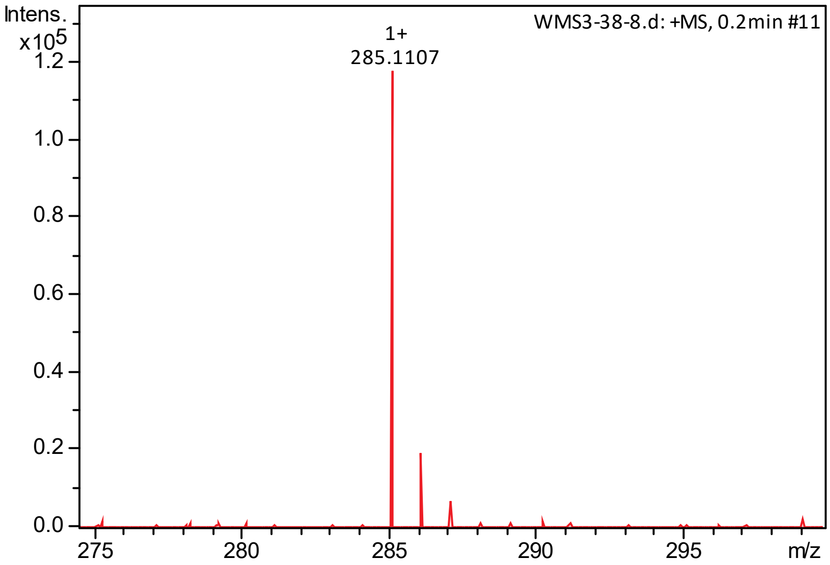

Figure S7. HRESIMS spectrum of aurantiophilane A (**1**) in CD_3OD.

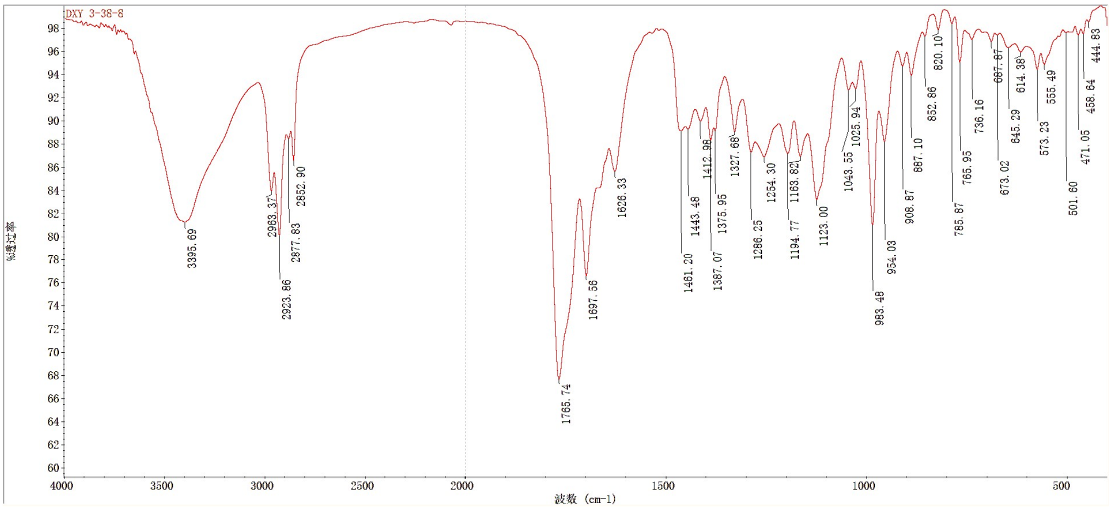

Figure S8. IR spectrum of aurantiophilane A (**1**) in CD_3OD.

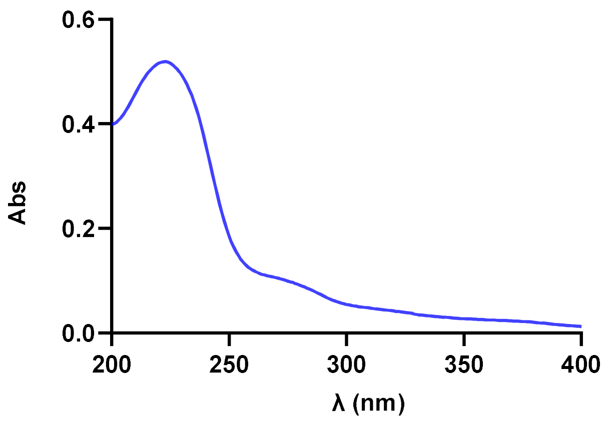

Figure S9. UV spectrum of aurantiophilane A (**1**) in MeCN.

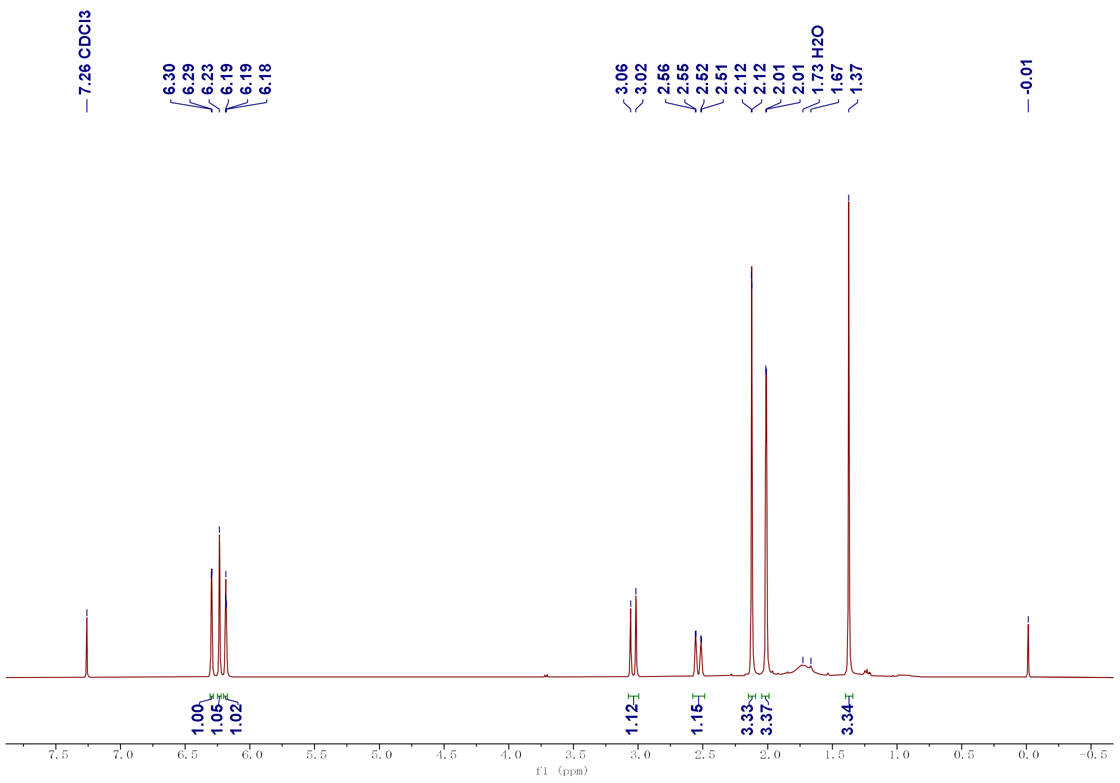

Figure S10. ^1H NMR spectrum of aurantiophilane B (**2**) in $CDCl_3$.

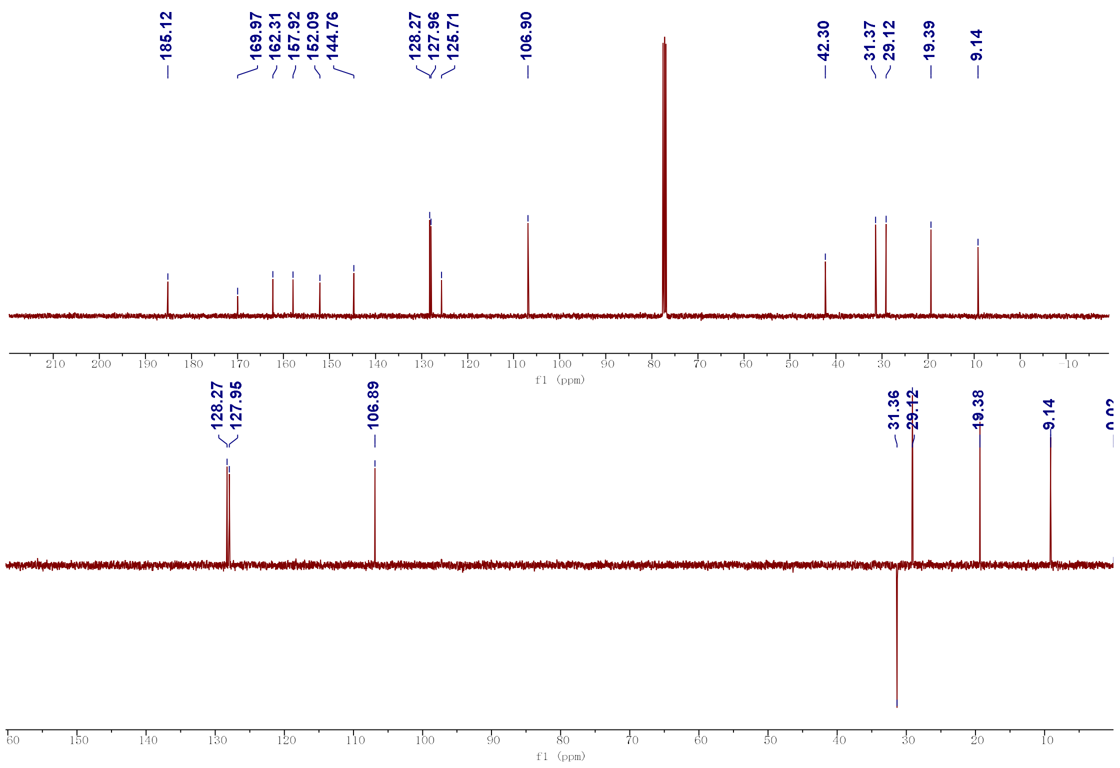

Figure S11. ^{13}C NMR spectrum of aurantiophilane B (**2**) in $CDCl_3$.

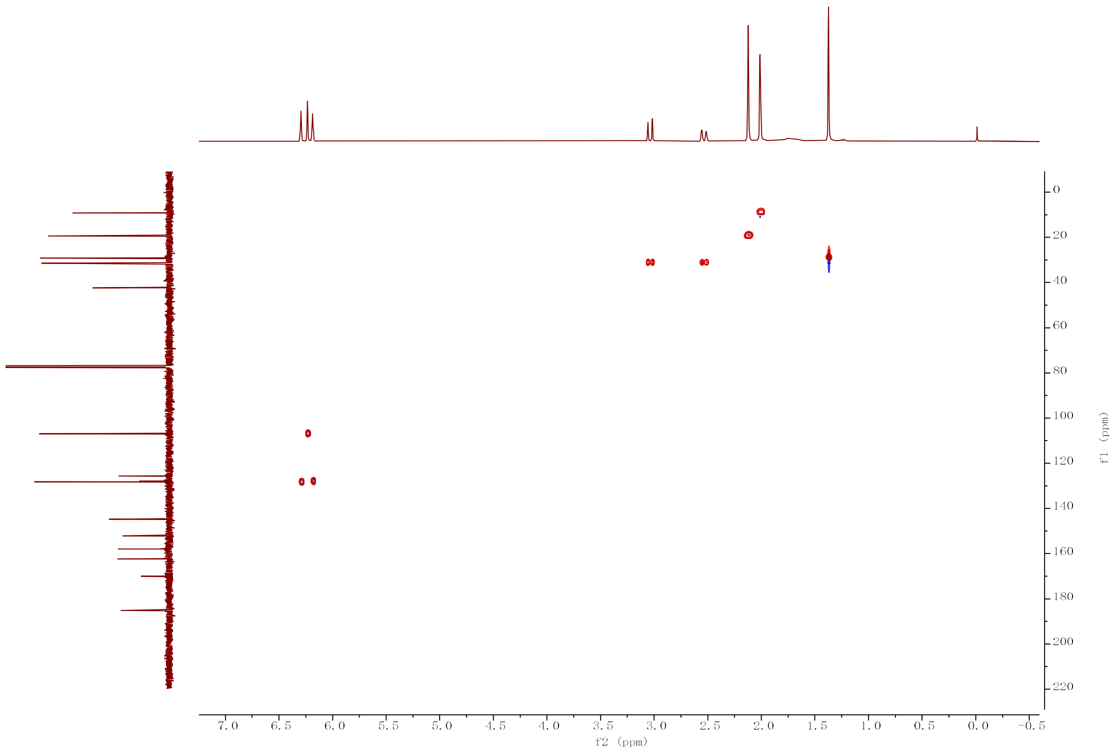

Figure S12. HSQC spectrum of aurantiophilane B (**2**) in $CDCl_3$.

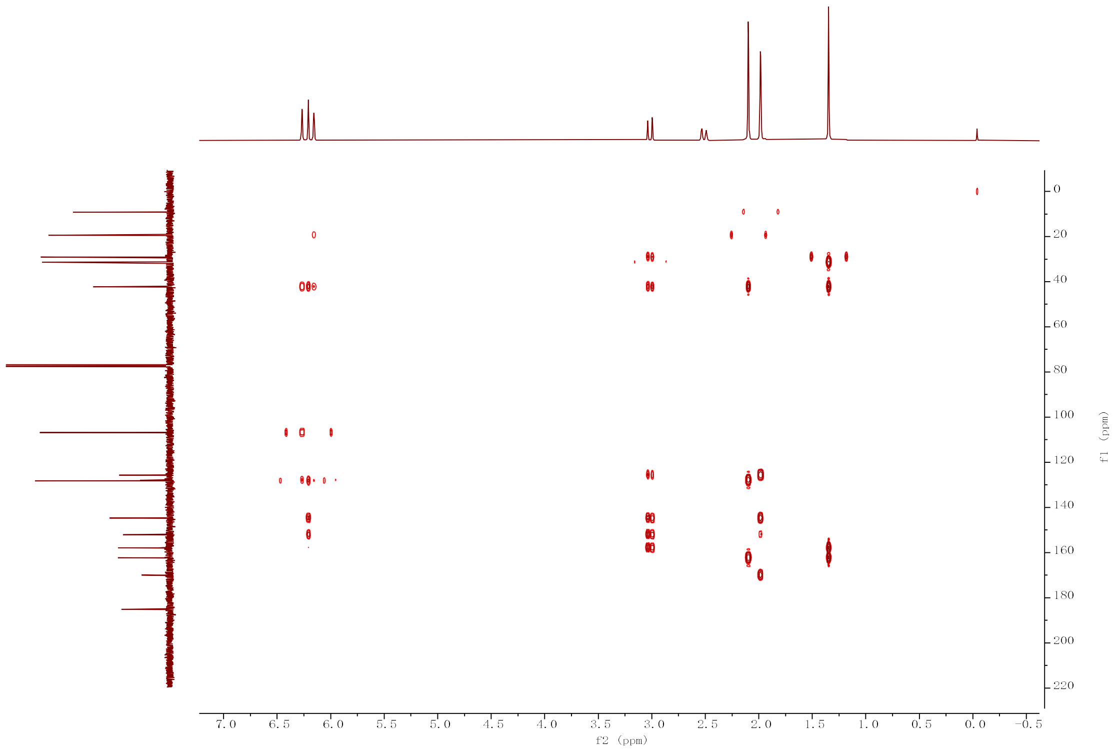

Figure S13. HMBC spectrum of aurantiophilane B (**2**) in $CDCl_3$.

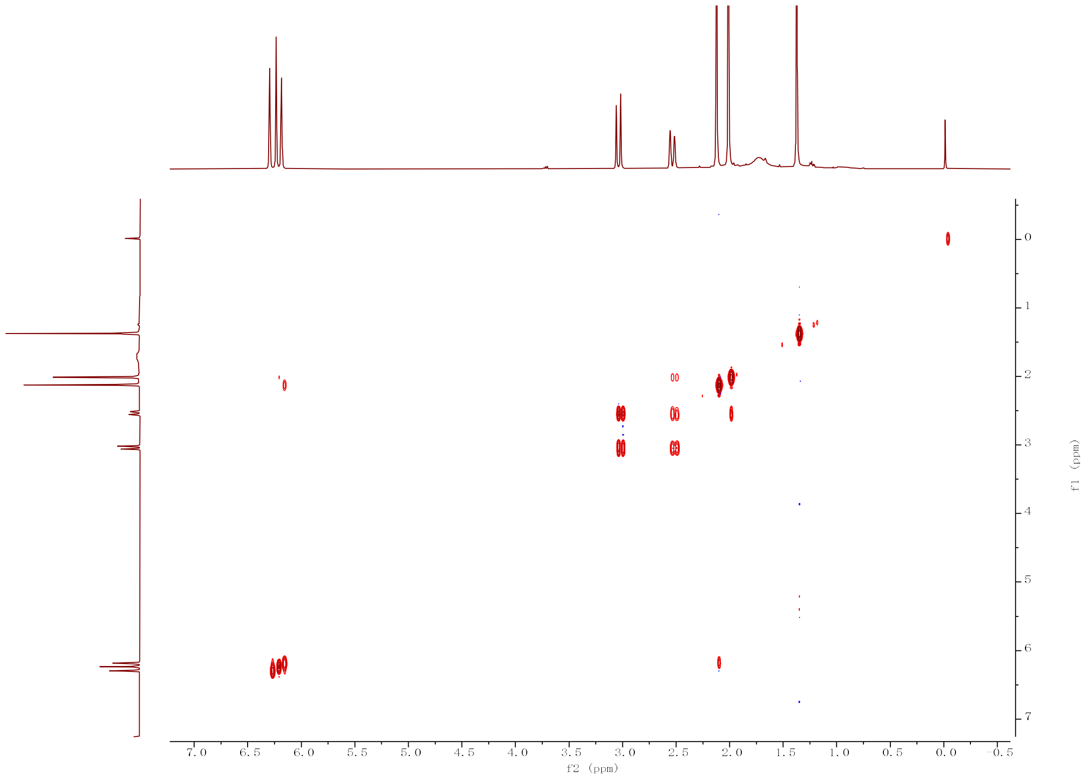

Figure S14. ^1H–^1H COSY spectrum of aurantiophilane B (**2**) in $CDCl_3$.

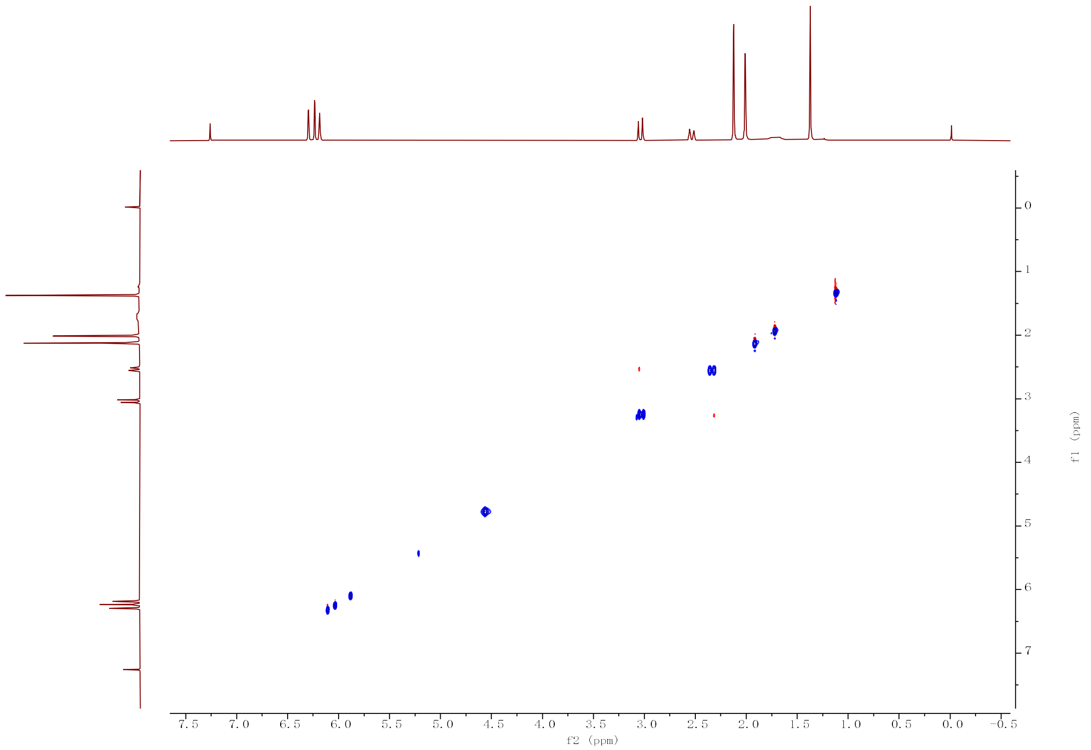

Figure S15. NOESY spectrum of aurantiophilane B (**2**) in $CDCl_3$.

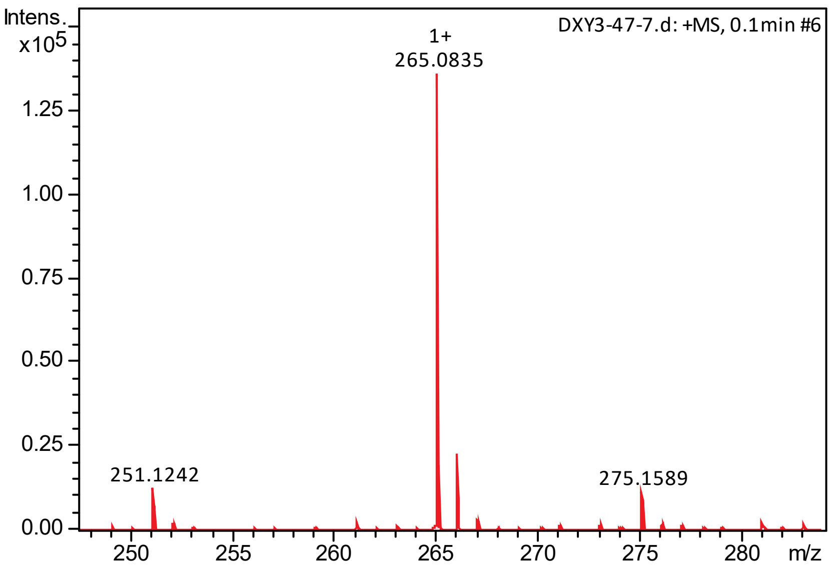

Figure S16. HRESIMS spectrum of aurantiophilane B (**2**) in $CDCl_3$.

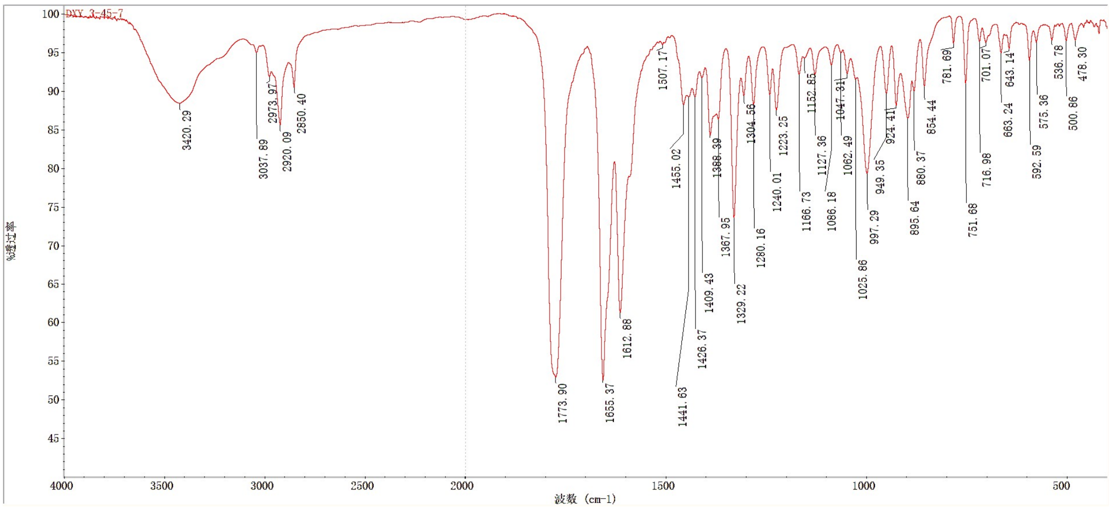

Figure S17. IR spectrum of aurantiophilane B (**2**) in $CDCl_3$.

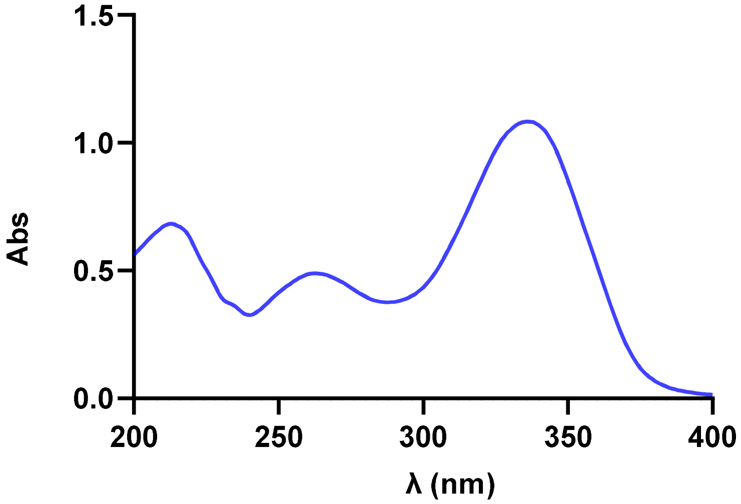

Figure S18. UV spectrum of aurantiophilane B (**2**) in MeCN.

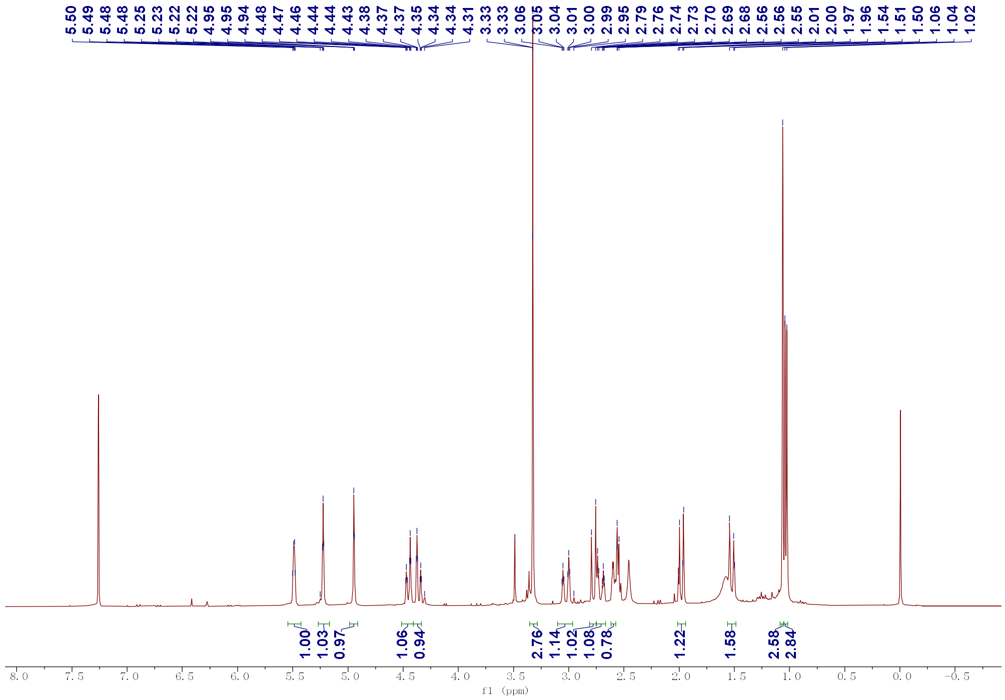

Figure S19. ^{1}H NMR spectrum of aurantiophilane C (**3**) in $CDCl_3$.

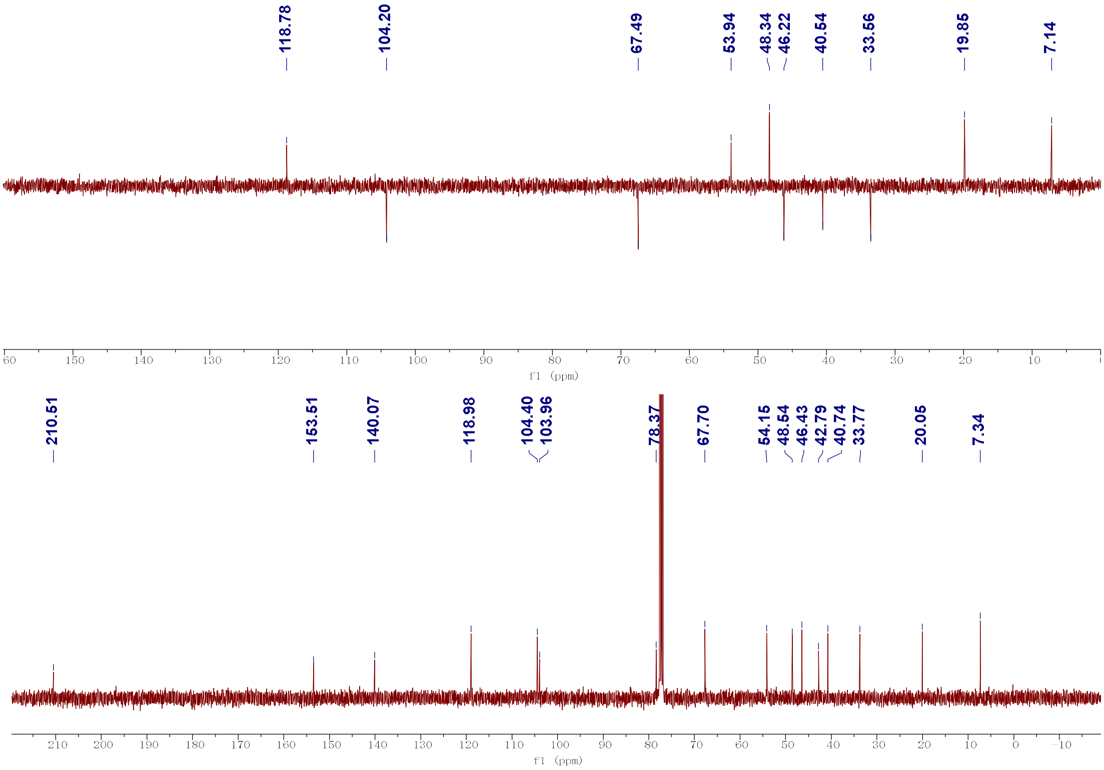

Figure S20. 13C NMR spectrum of aurantiophilane C (**3**) in CDCl3.

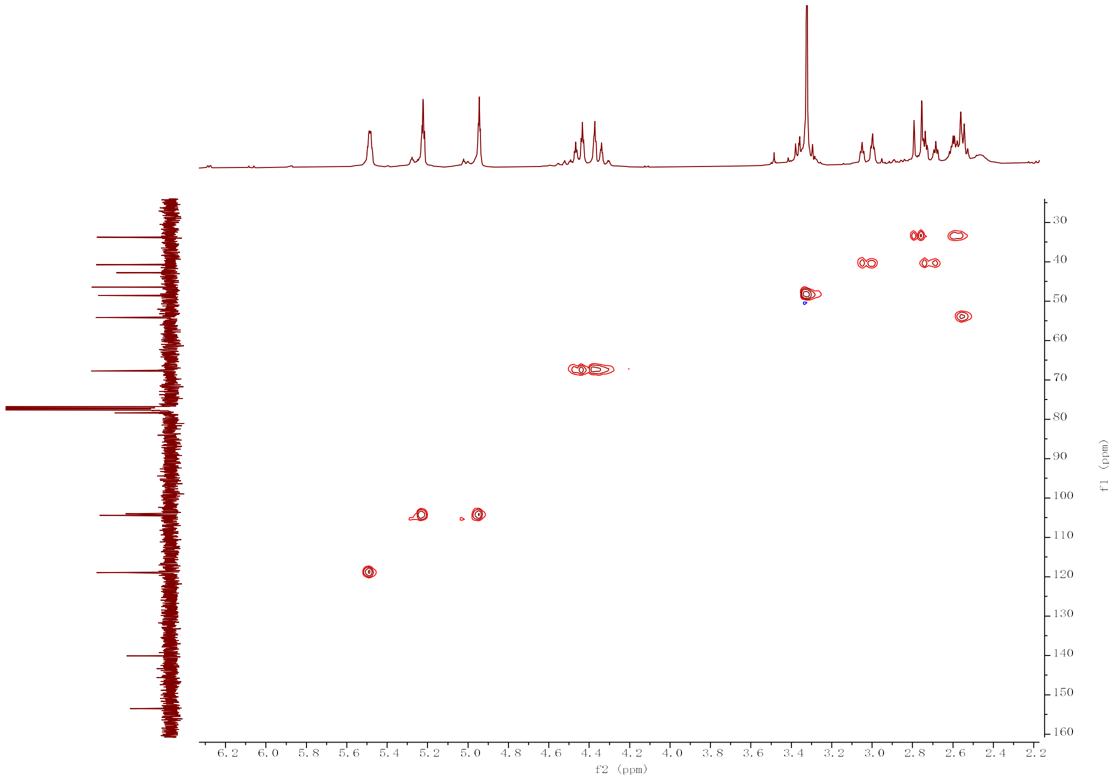

Figure S21. HSQC spectrum of aurantiophilane C (**3**) in $CDCl_3$.

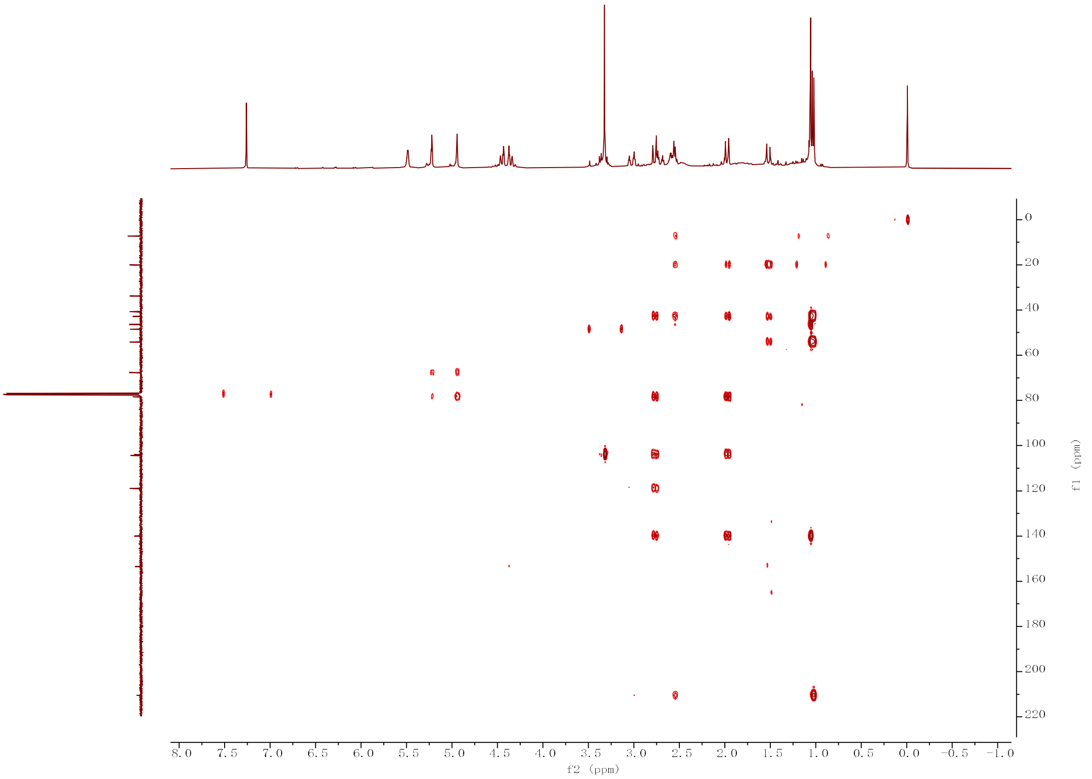

Figure S22. HMBC spectrum of aurantiophilane C (**3**) in $CDCl_3$.

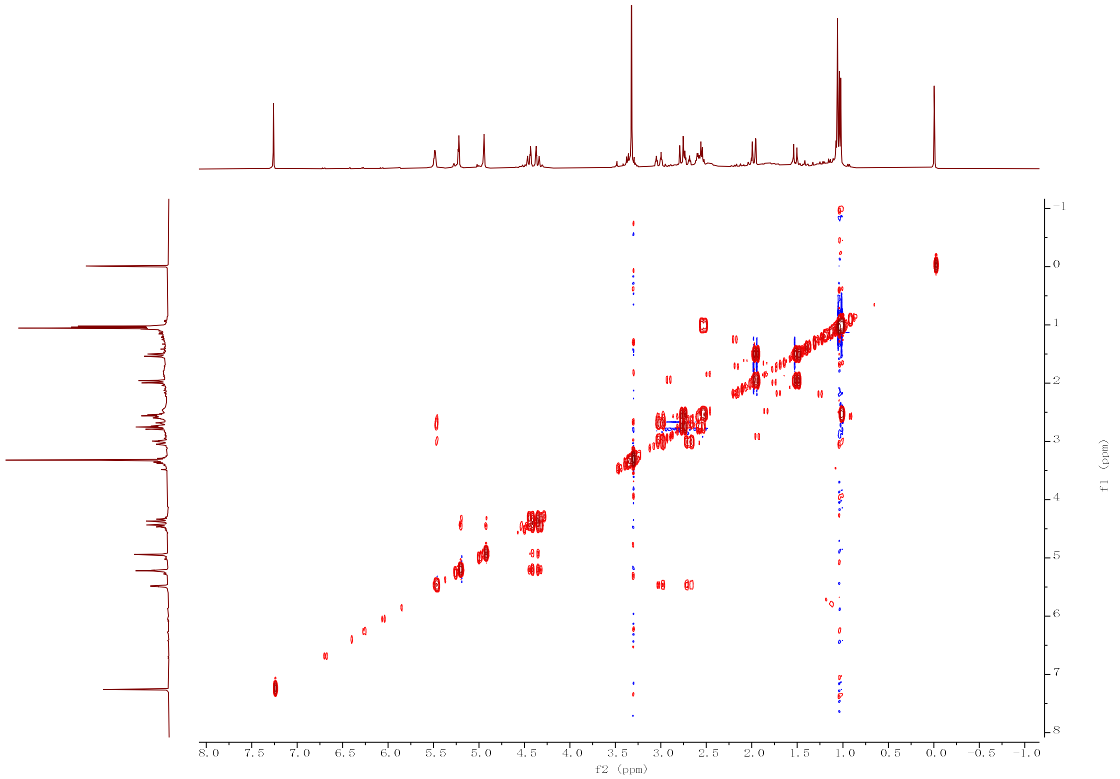

Figure S23. ^{1}H–^{1}H COSY spectrum of aurantiophilane C (**3**) in $CDCl_3$.

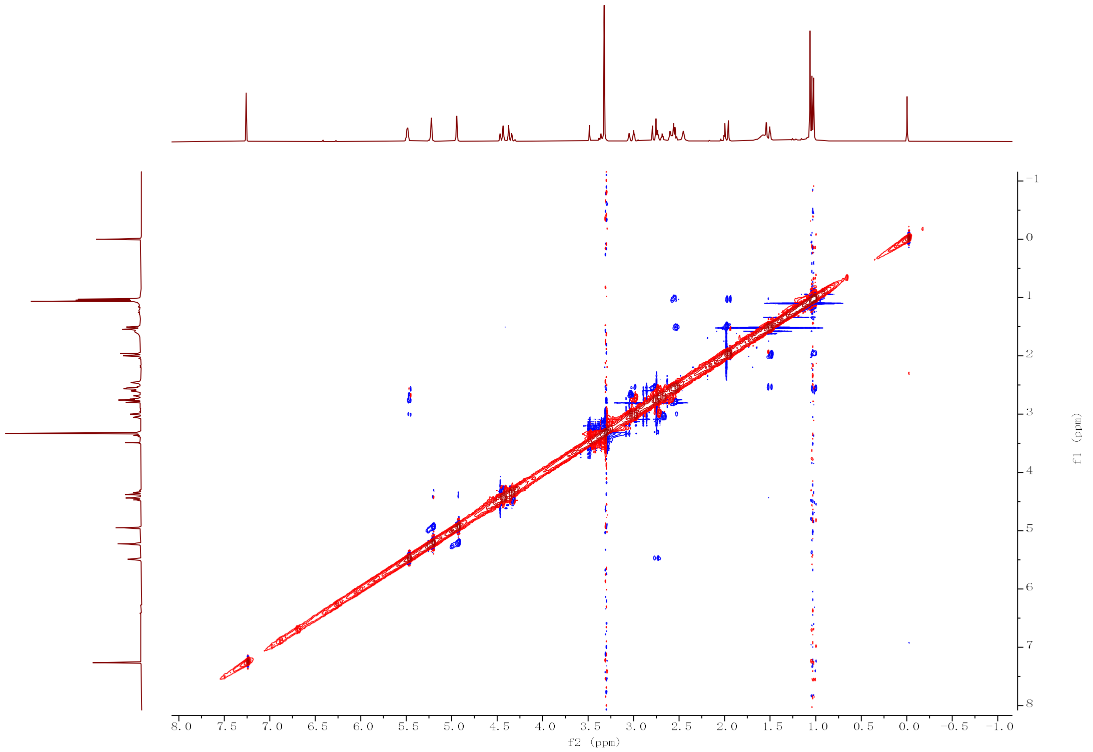

Figure S24. NOESY spectrum of aurantiophilane C (**3**) in $CDCl_3$.

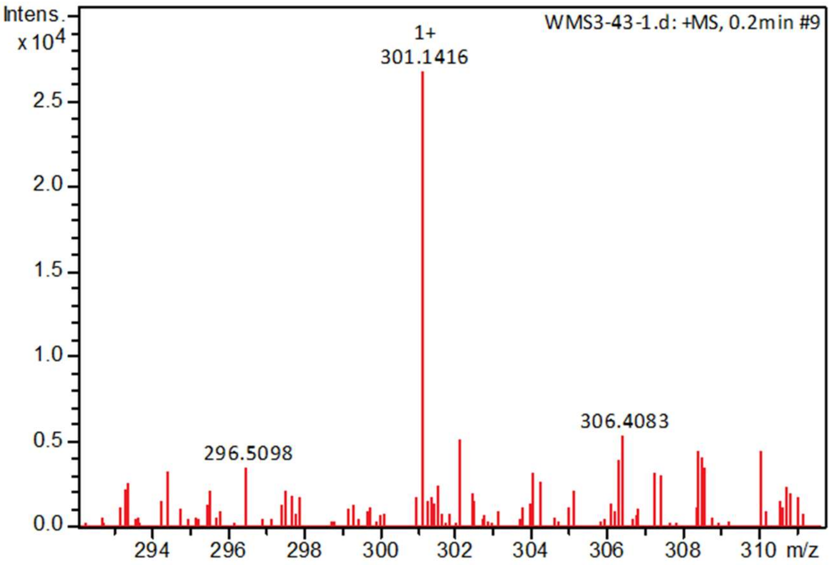

Figure S25. HRESIMS spectrum of aurantiophilane C (**3**) in $CDCl_3$.

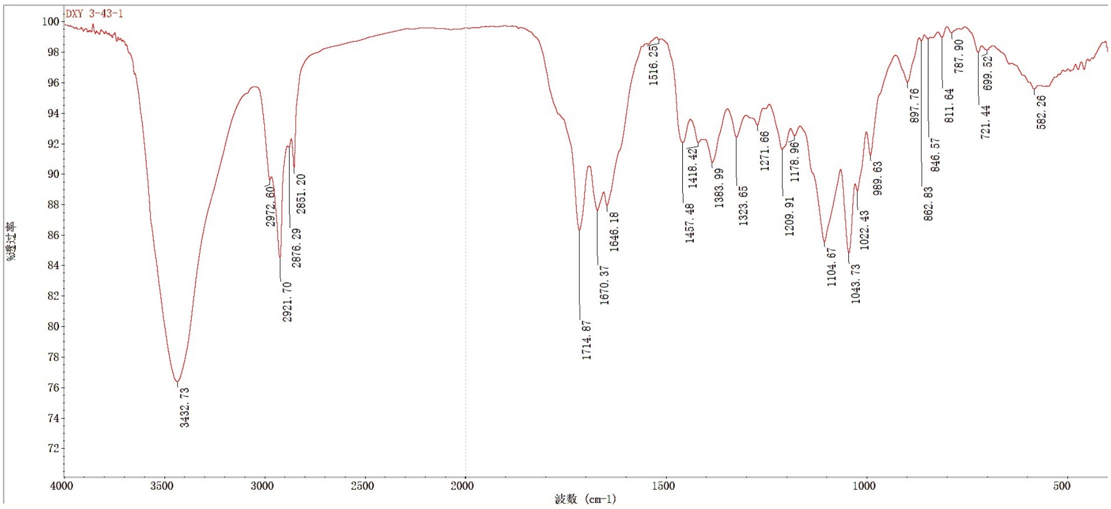

Figure S26. IR spectrum of aurantiophilane C (**3**) in $CDCl_3$.

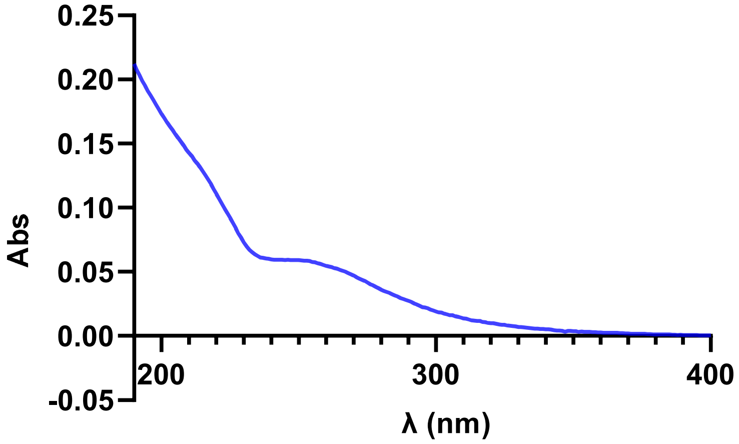

Figure S27. UV spectrum of aurantiophilane C (**3**) in MeCN.

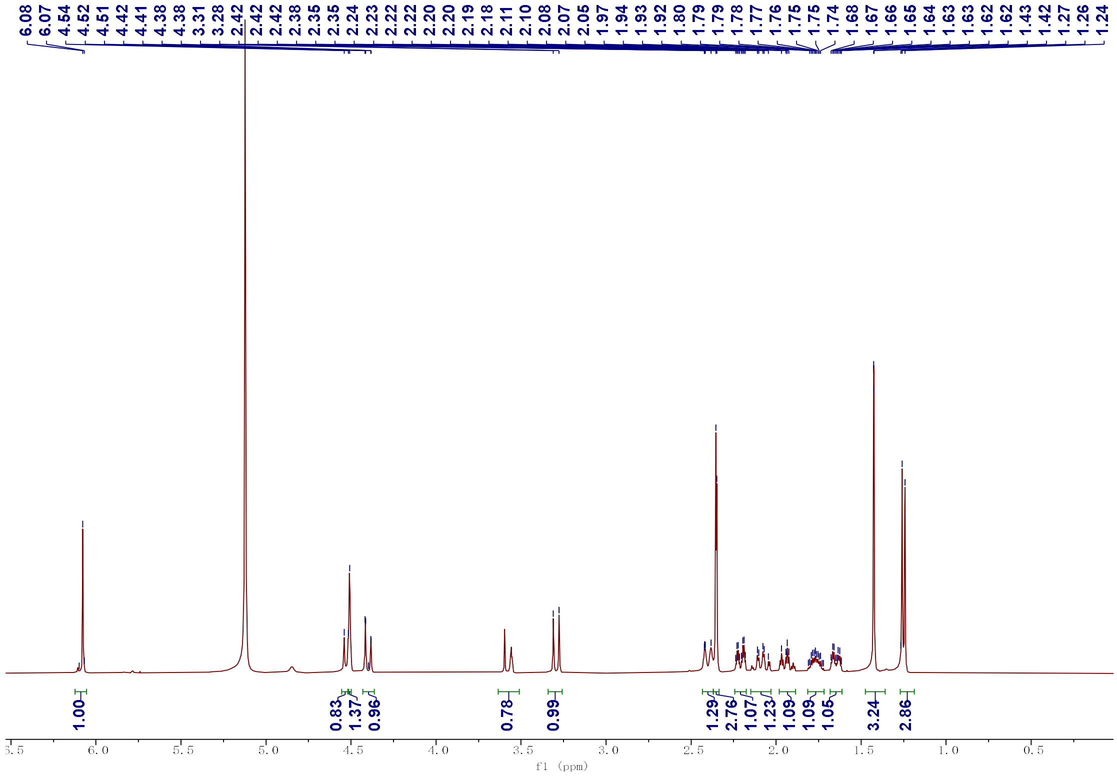

Figure S28. ^{1}H NMR spectrum of aurantiophilane E (**4**) in CD_3OD.

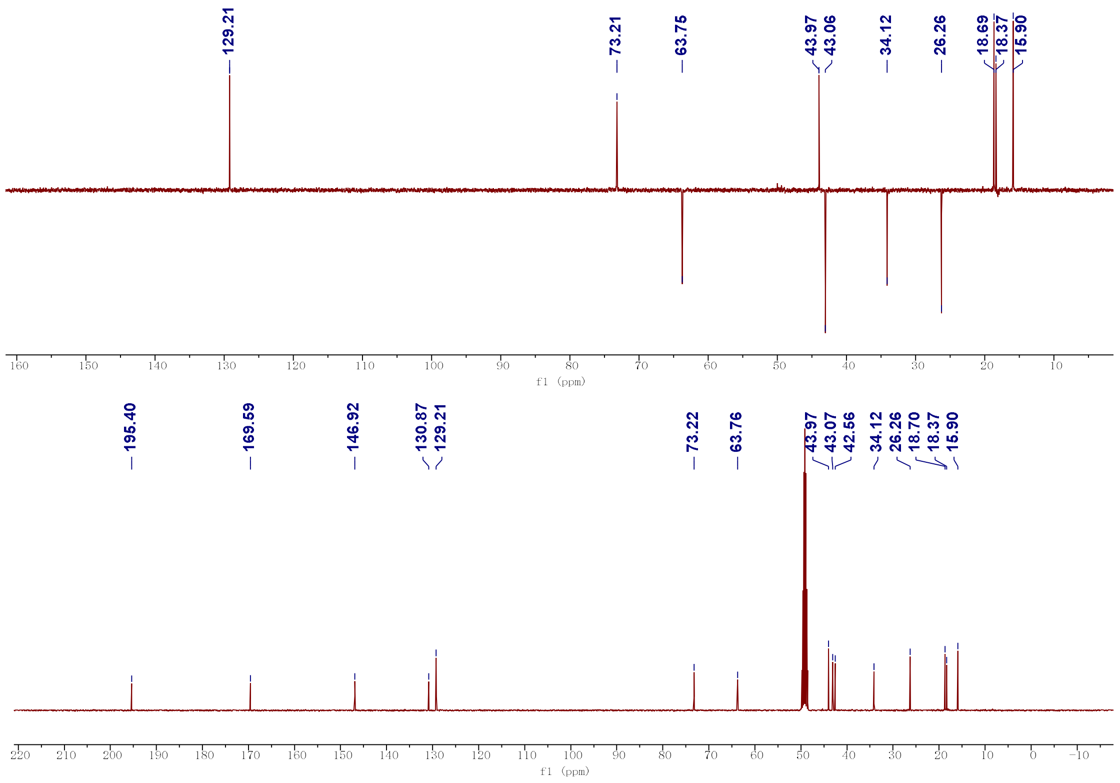

Figure S19. ^{13}C NMR spectrum of aurantiophilane E (**4**) in CD_3OD.

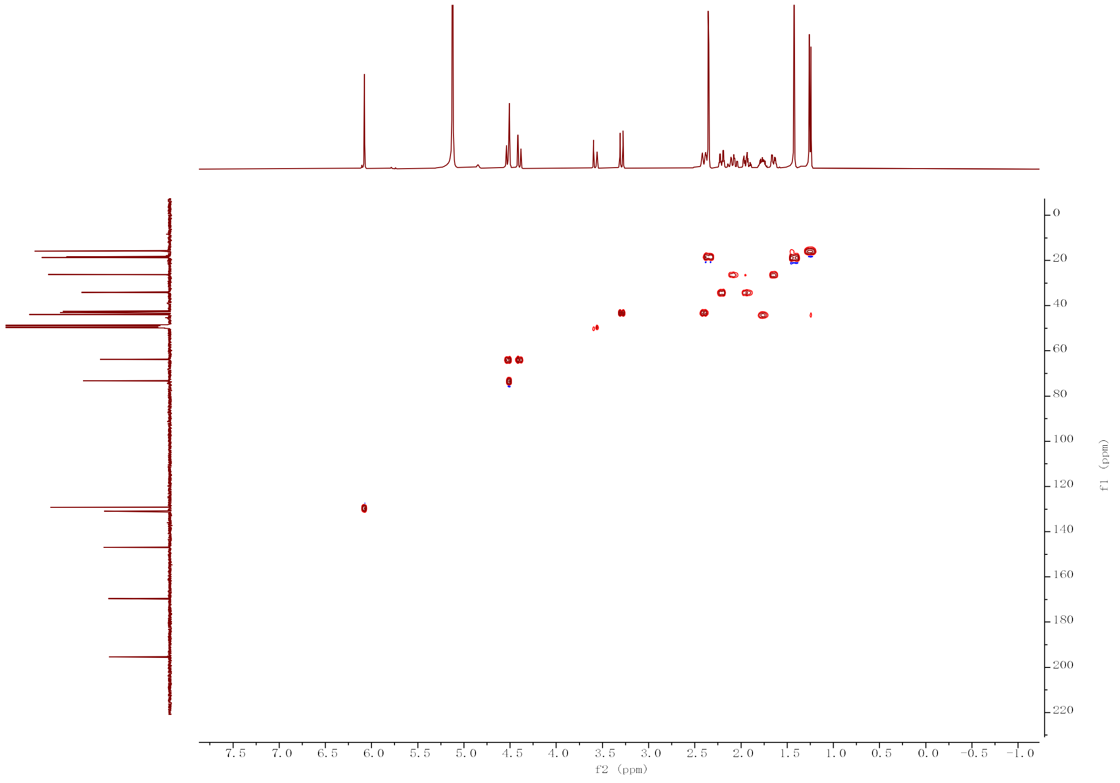

Figure S20. HSQC spectrum of aurantiophilane E (**4**) in CD_3OD.

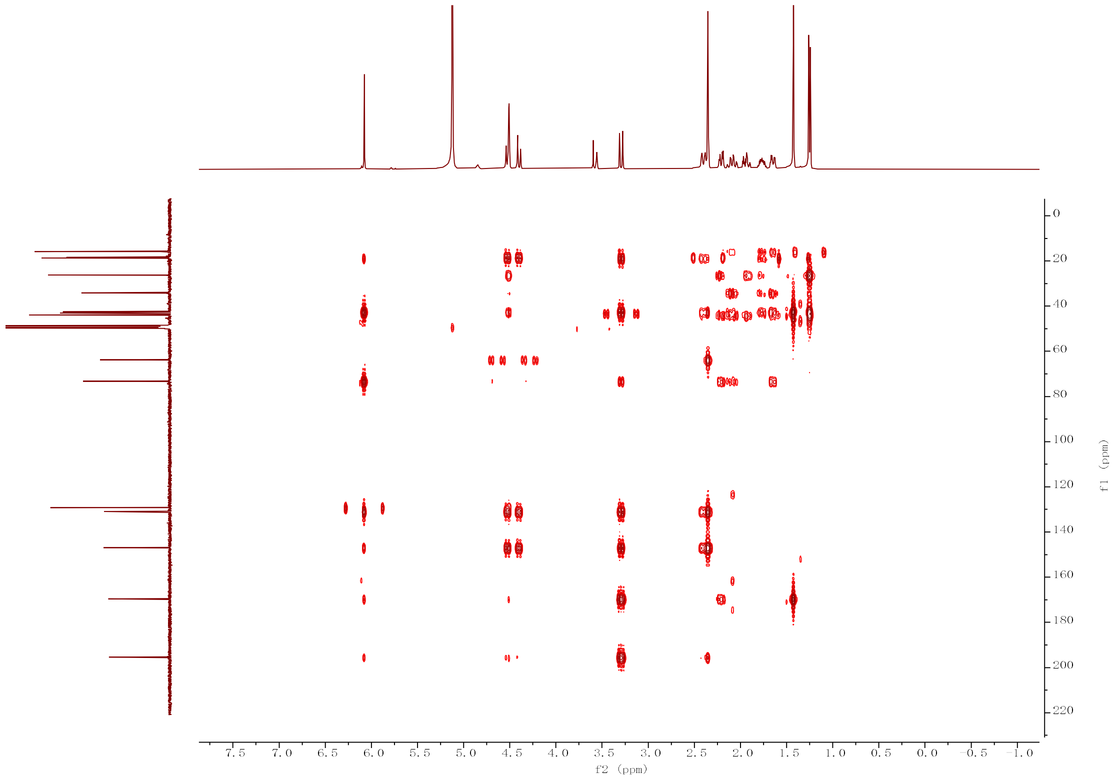

Figure S31. HMBC spectrum of aurantiophilane E (**4**) in CD_3OD.

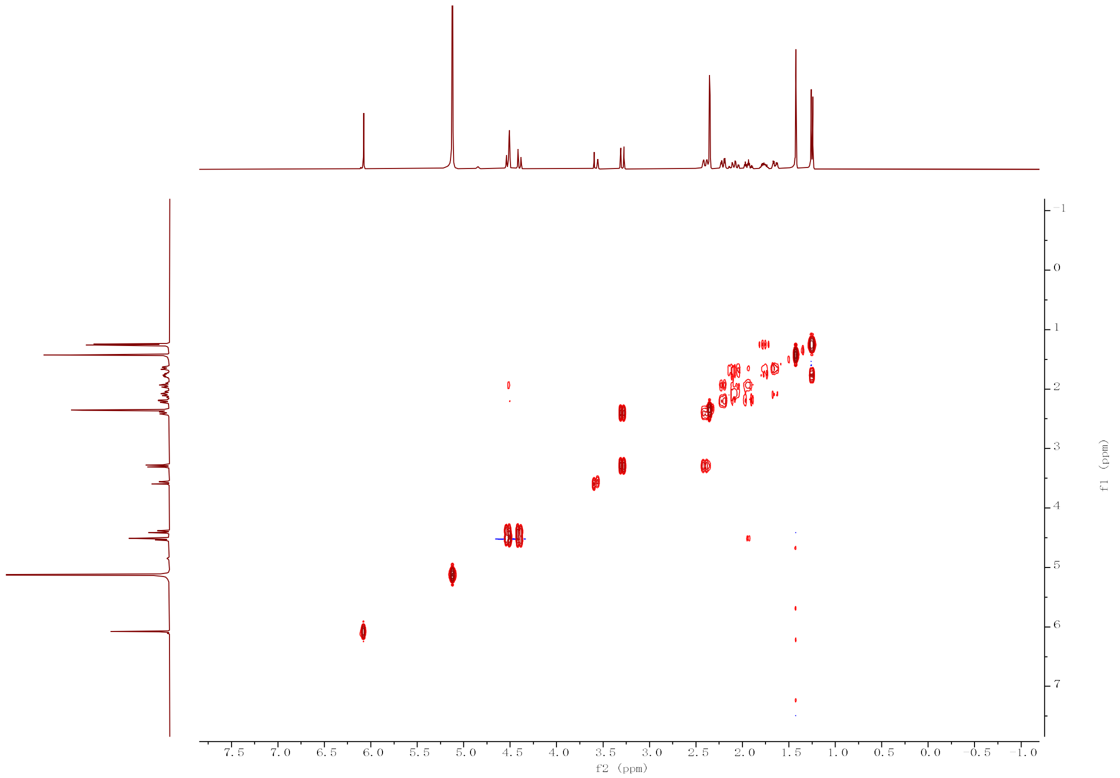

Figure S32. ^1H–^1H COSY spectrum of aurantiophilane E (**4**) in CD_3OD.

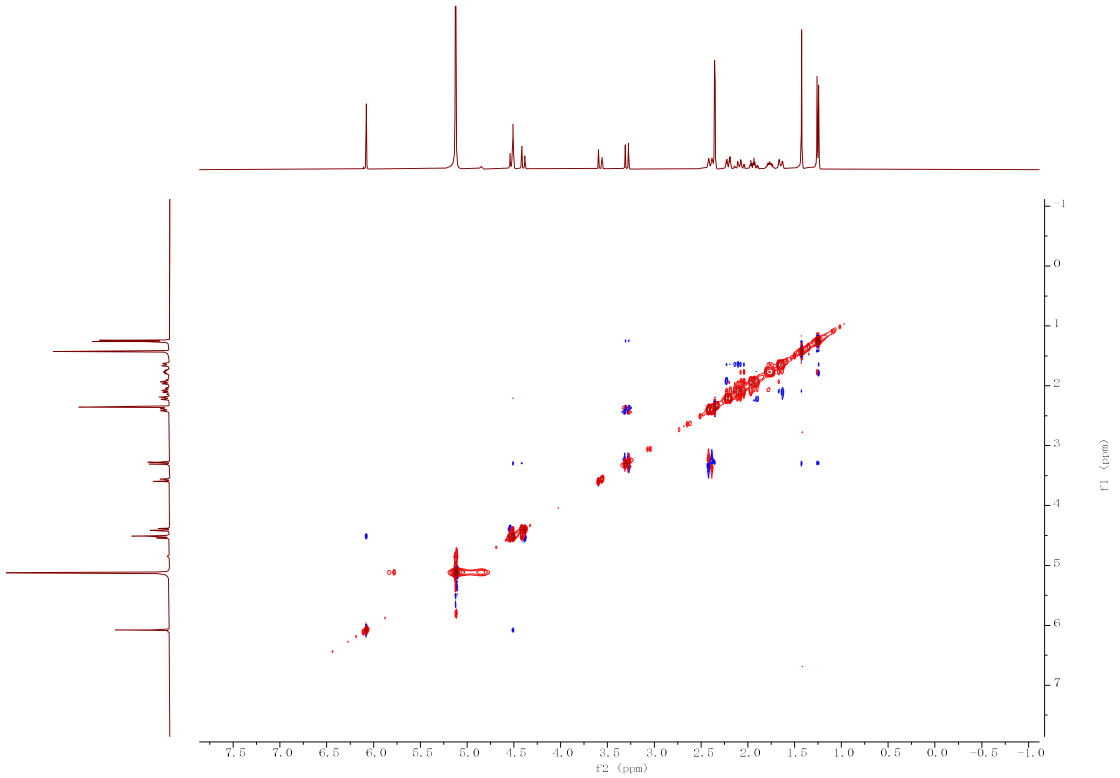

Figure S33. NOESY spectrum of aurantiophilane E (**4**) in CD_3OD.

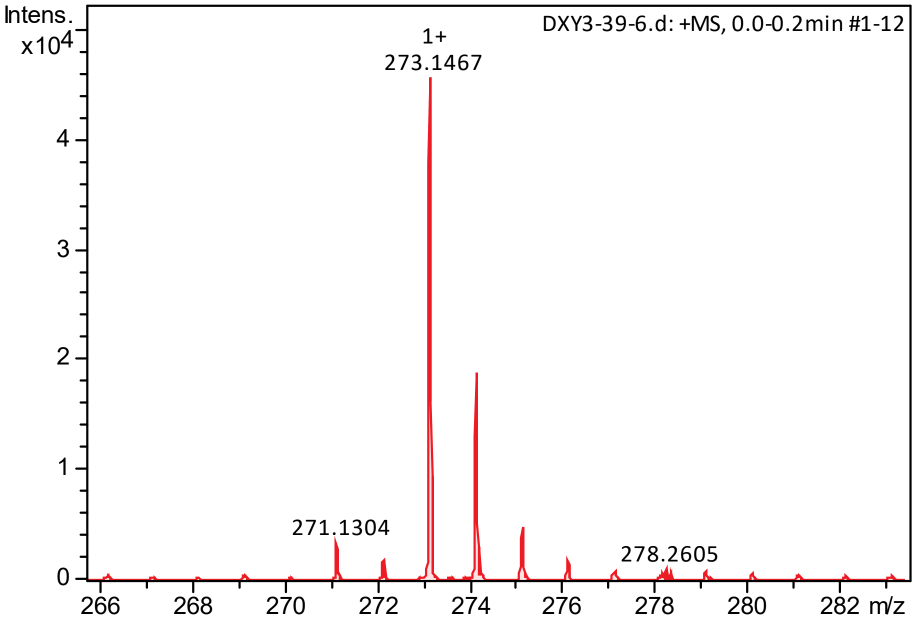

Figure S34. HRESIMS spectrum of aurantiophilane E (**4**) in CD_3OD.

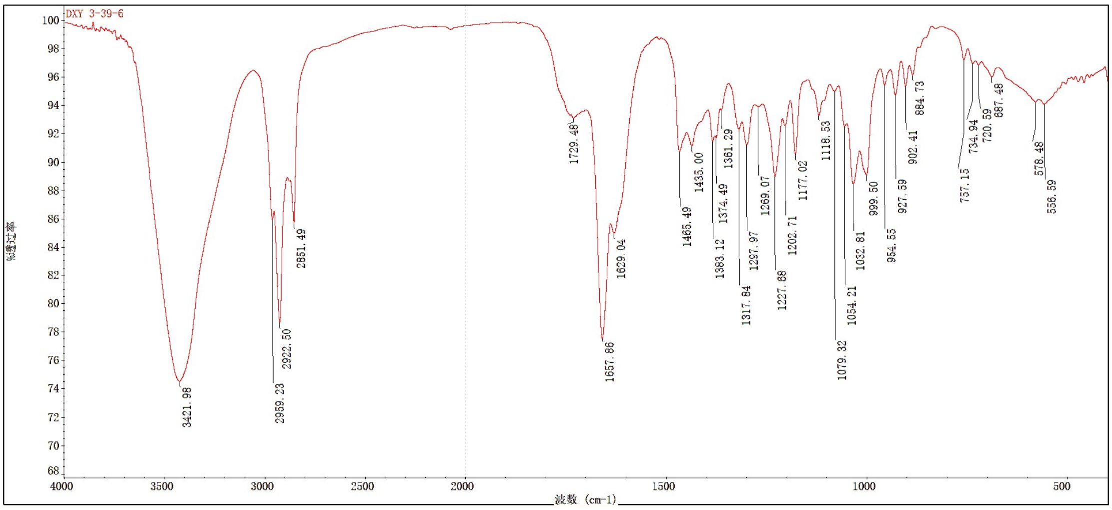

Figure S35. IR spectrum of aurantiophilane E (**4**) in CD_3OD.

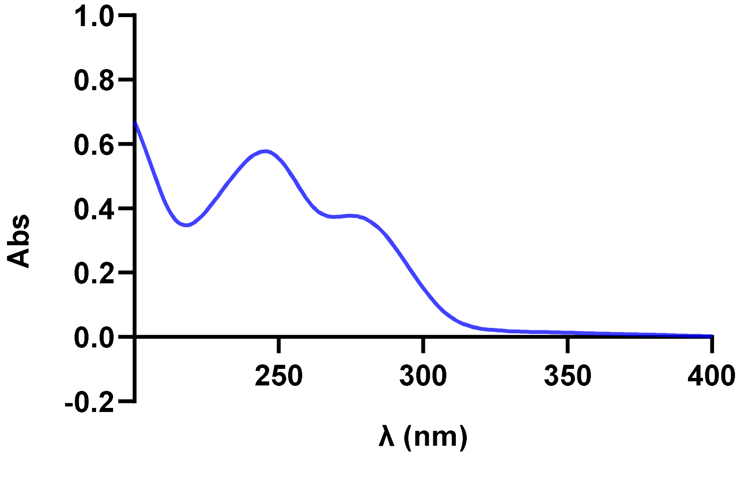

Figure S36. UV spectrum of aurantiophilane E (**4**) in MeCN.

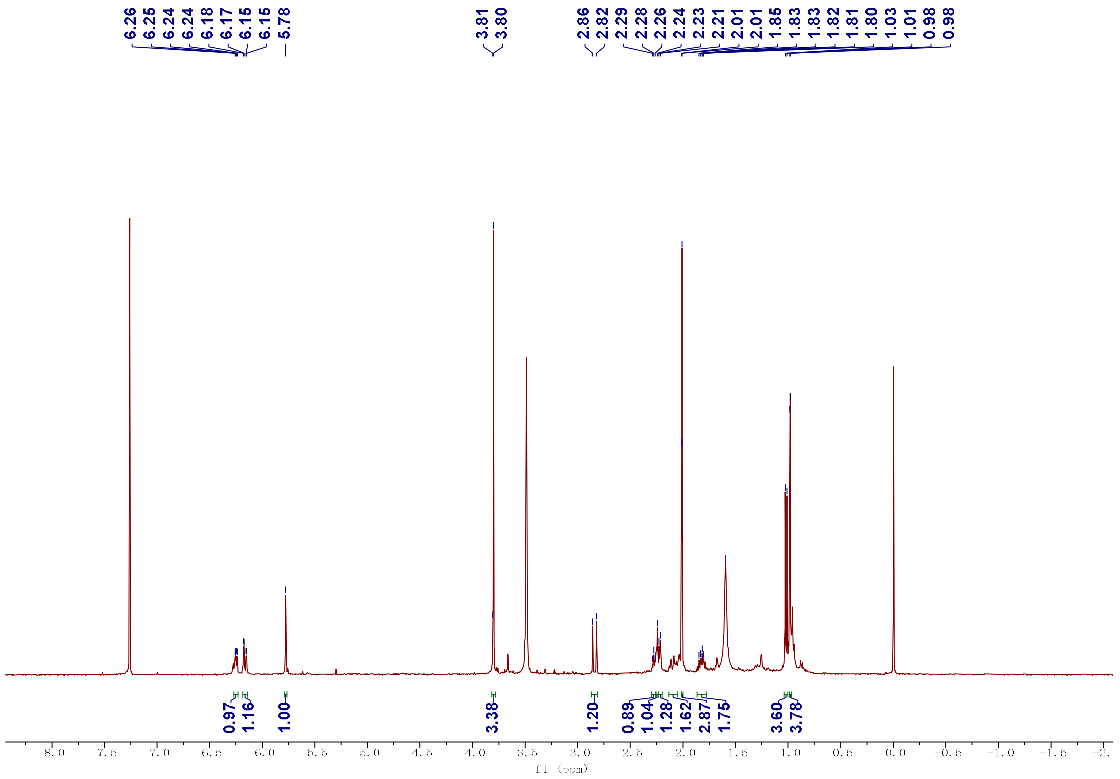

Figure S373. ^{1}H NMR spectrum of aurantiophilane F (**5**) in $CDCl_3$.

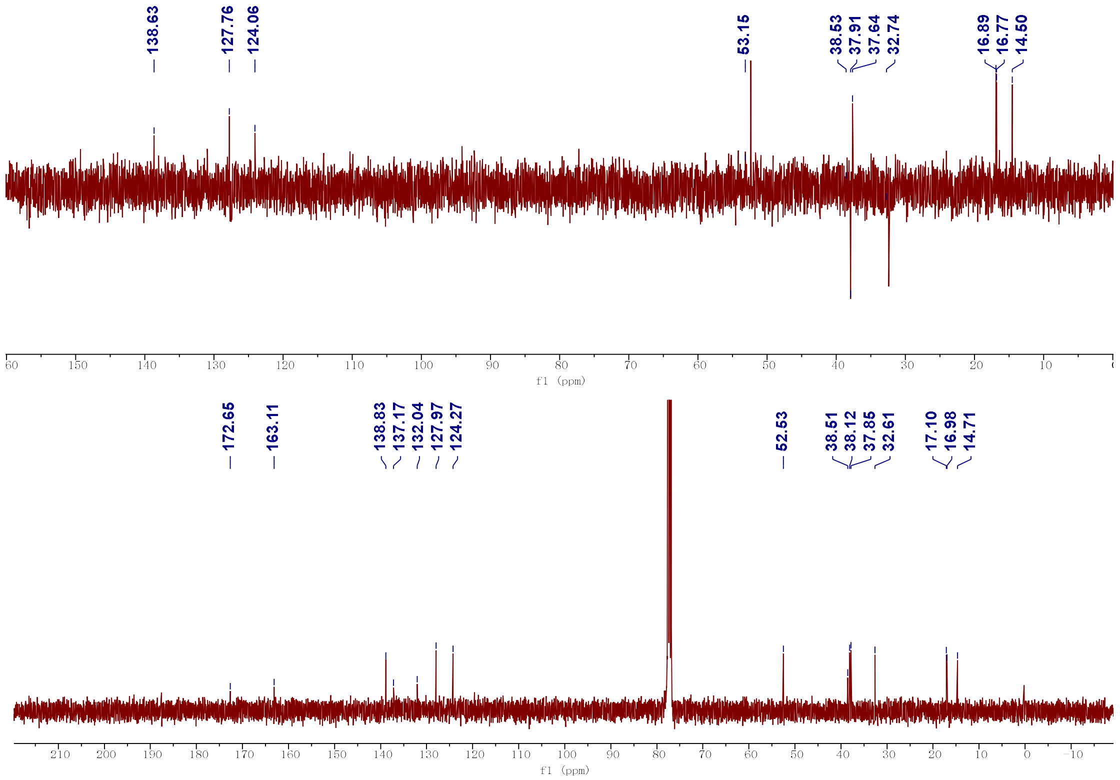

Figure S38. ^{13}C NMR spectrum of aurantiophilane F (**5**) in $CDCl_3$.

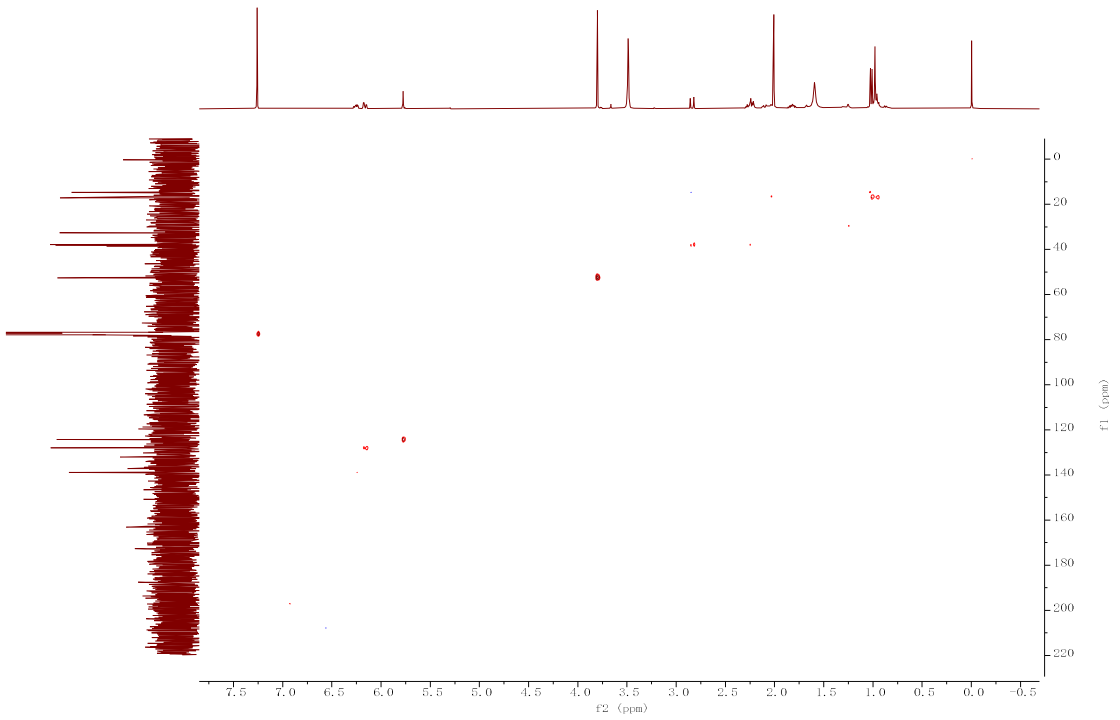

Figure S39. HSQC spectrum of aurantiophilane F (**5**) in $CDCl_3$.

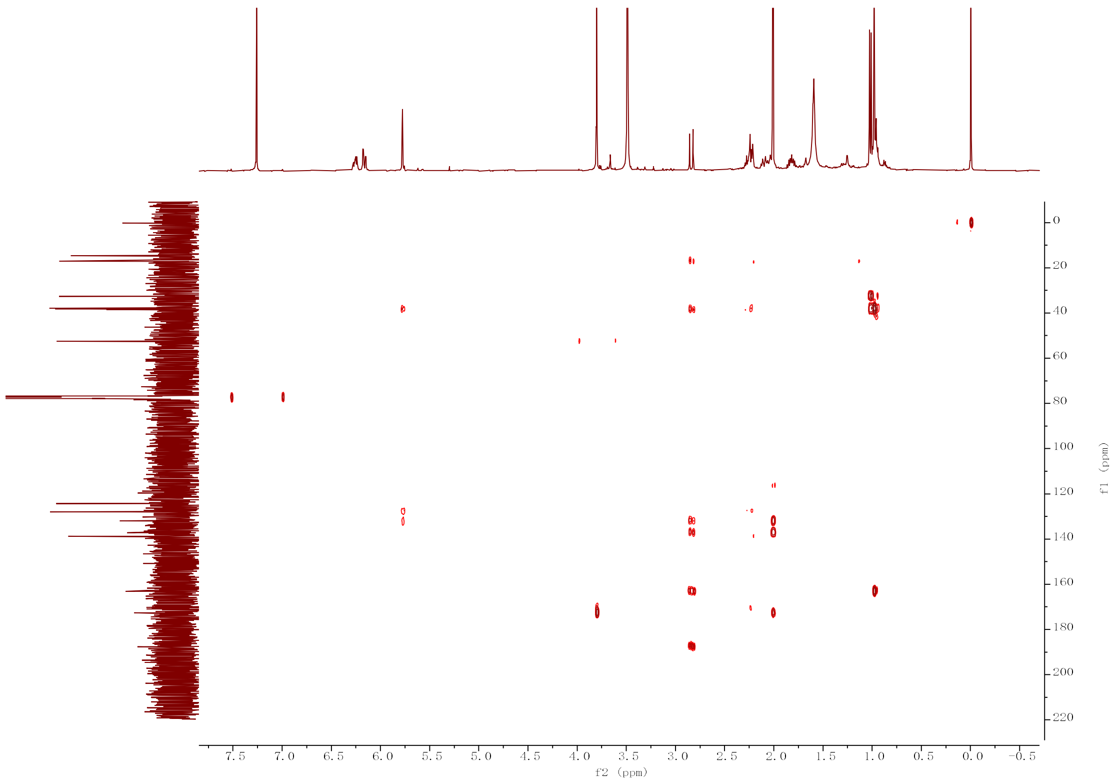

Figure S40. HMBC spectrum of aurantiophilane F (**5**) in $CDCl_3$.

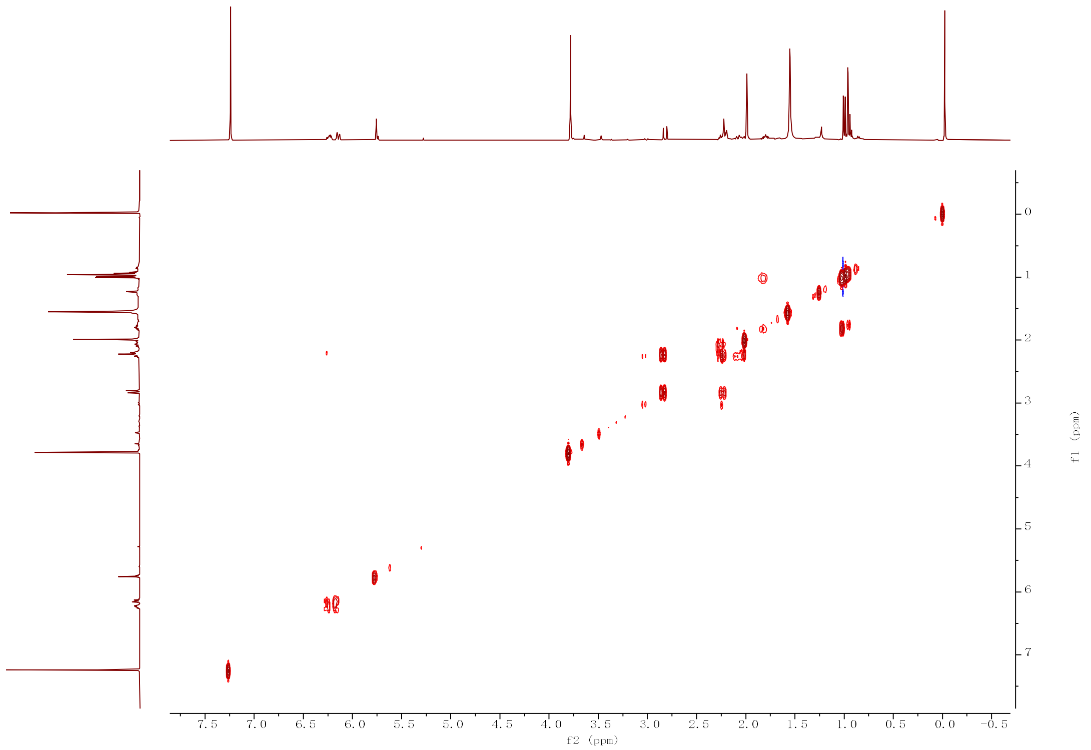

Figure S41. ^{1}H–^{1}H COSY spectrum of aurantiophilane F (**5**) in $CDCl_3$.

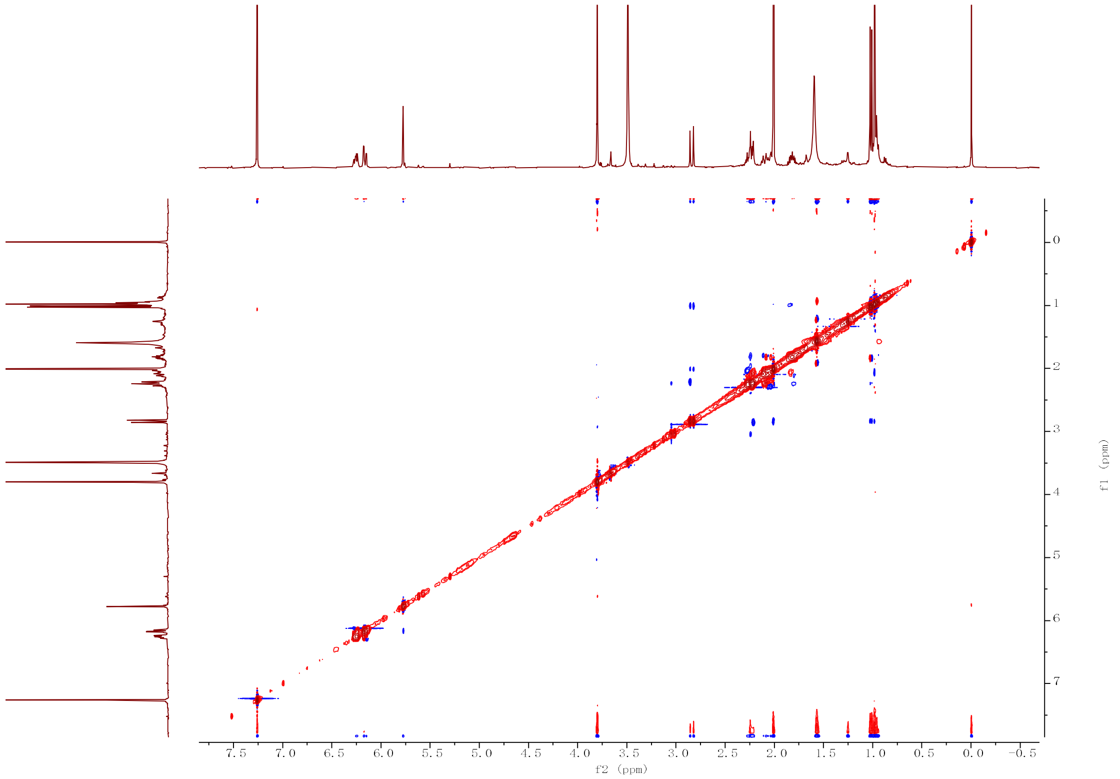

Figure S42. NOESY spectrum of aurantiophilane F (**5**) in CDCl3.

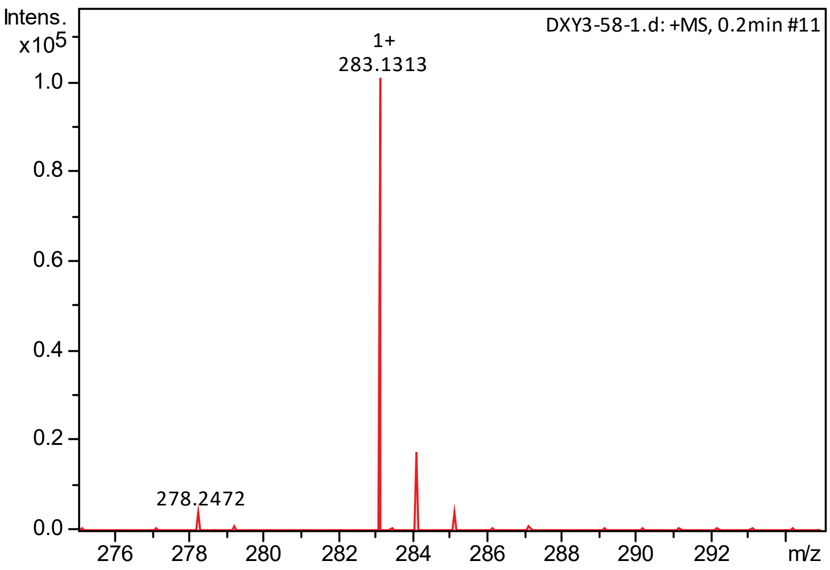

Figure S43. HRESIMS spectrum of aurantiophilane F (**5**) in $CDCl_3$.

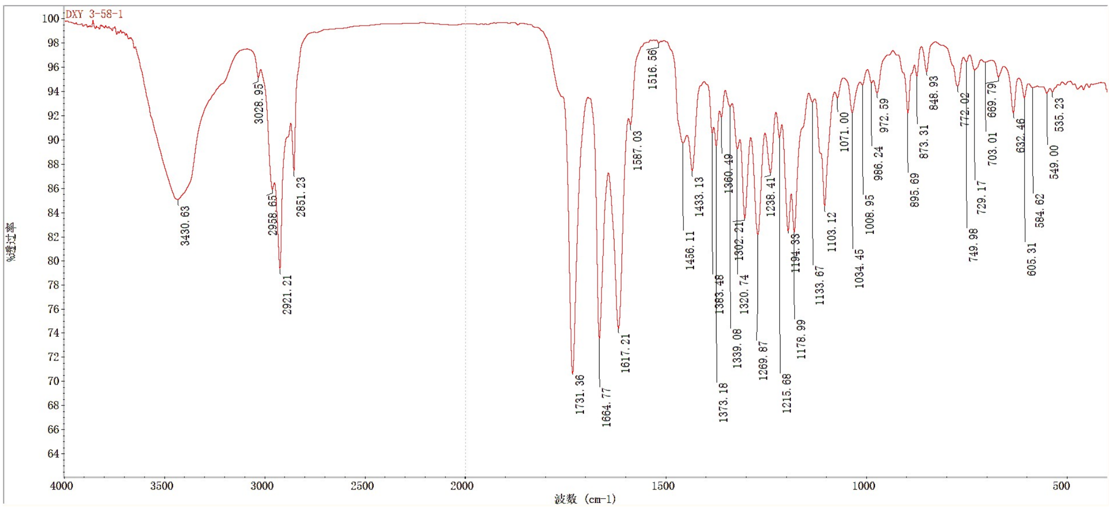

Figure S44. IR spectrum of aurantiophilane F (**5**) in $CDCl_3$.

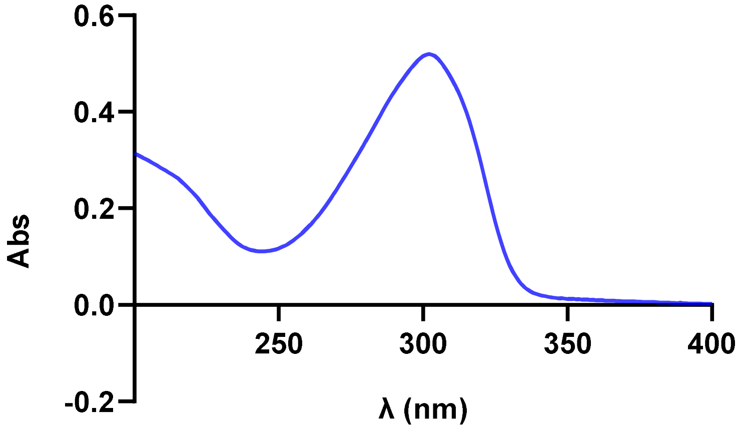

Figure S45. UV spectrum of aurantiophilane F (**5**) in MeCN.

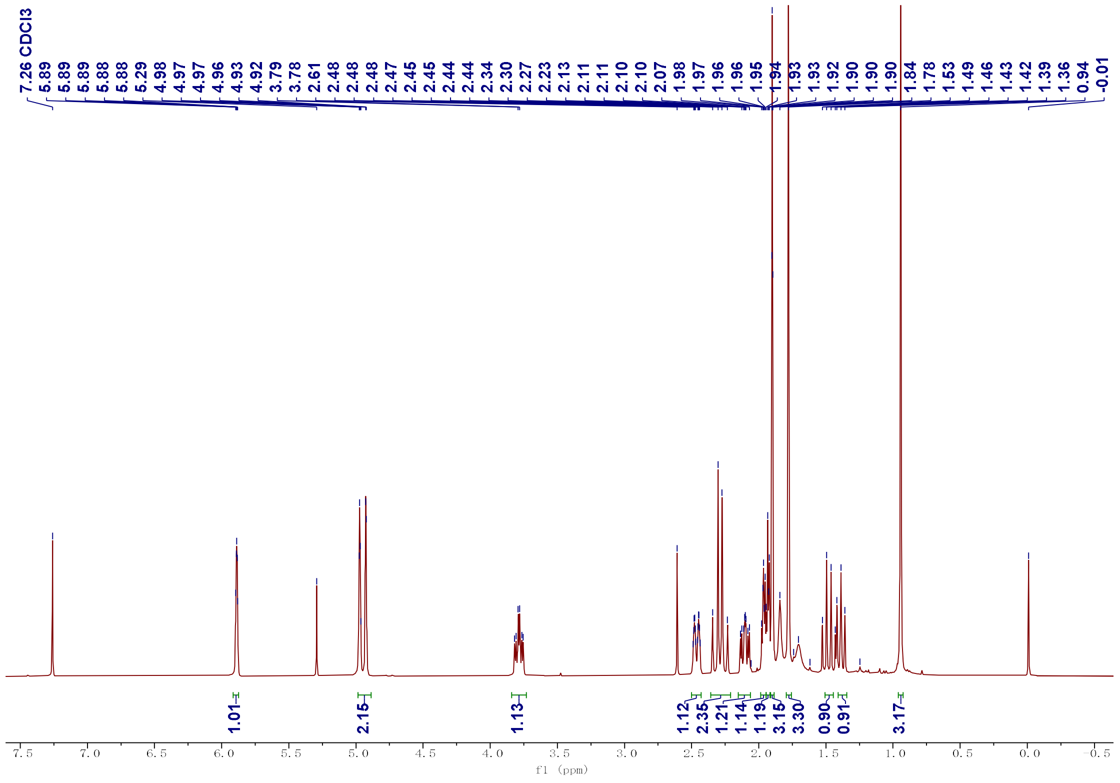

Figure S46. ^{1}H NMR spectrum of aurantiophilane G (**6**) in $CDCl_3$.

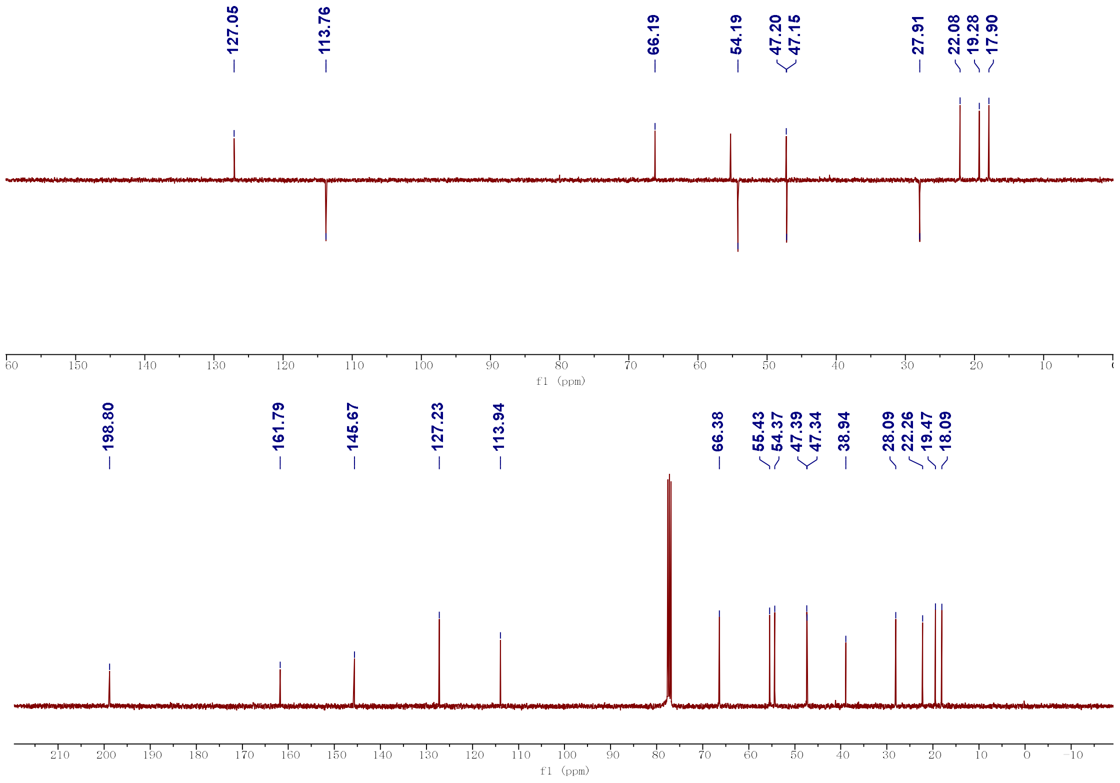

Figure S47. ^{13}C NMR spectrum of aurantiophilane G (**6**) in $CDCl_3$.

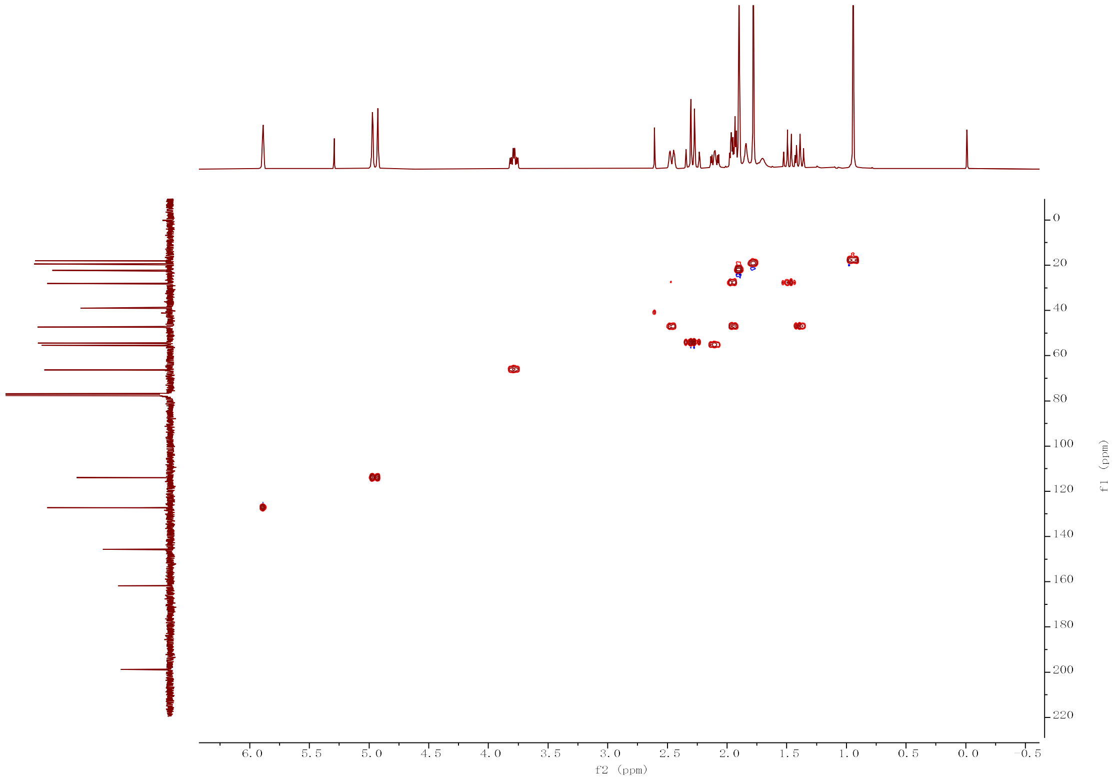

Figure S48. HSQC spectrum of aurantiophilane G (**6**) in $CDCl_3$.

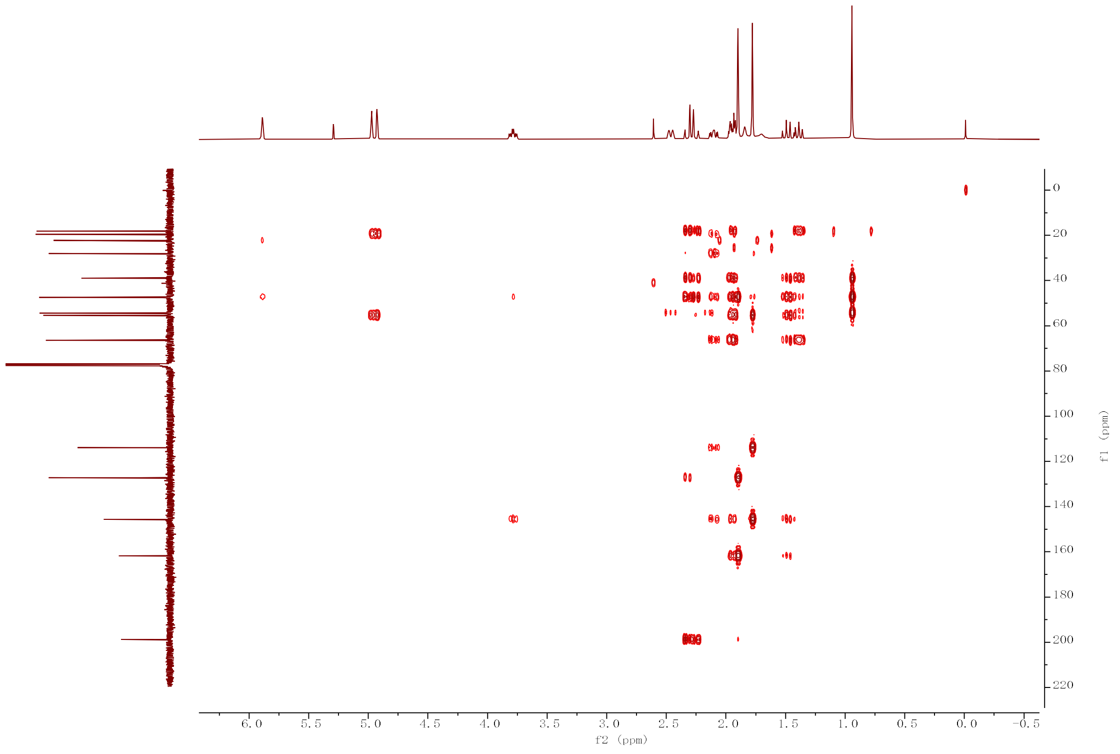

Figure S49. HMBC spectrum of aurantiophilane G (**6**) in $CDCl_3$.

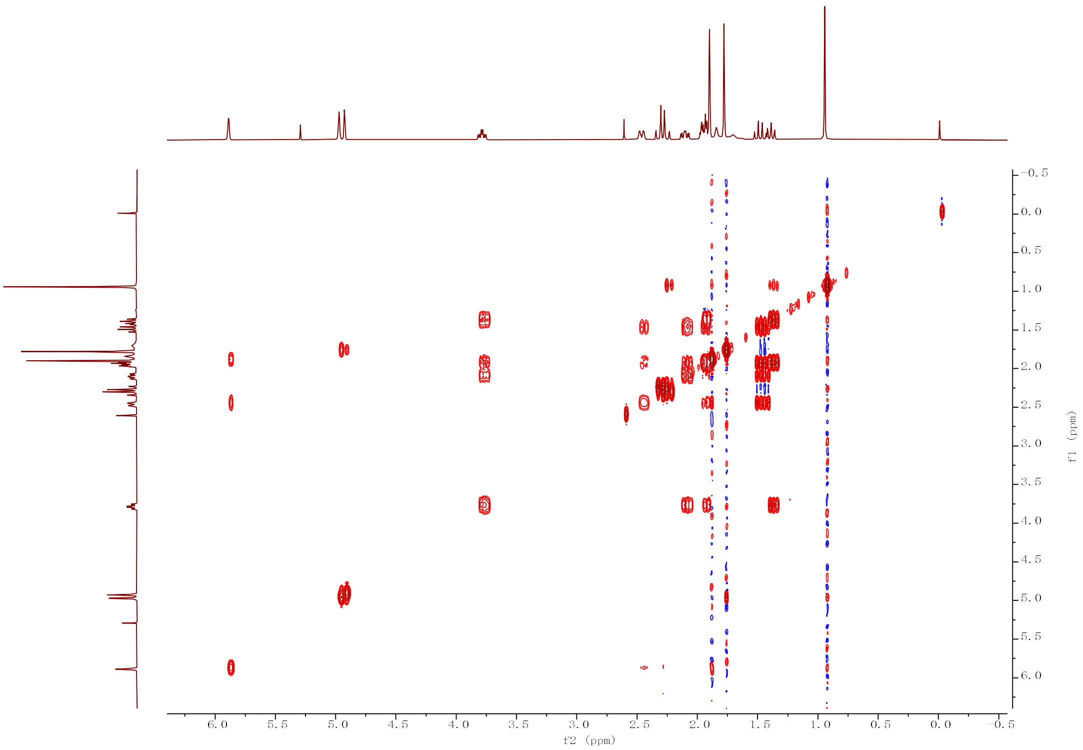

Figure S50. ^{1}H–^{1}H COSY spectrum of aurantiophilane G (**6**) in $CDCl_3$.

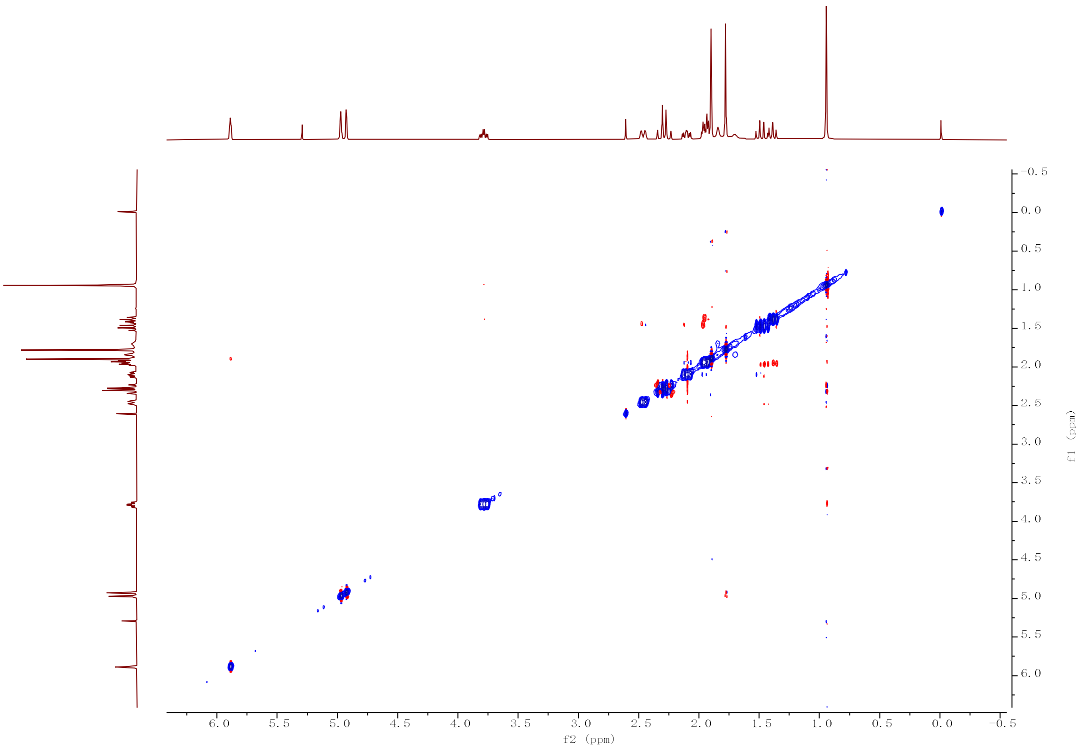

Figure S51. NOESY spectrum of aurantiophilane G (**6**) in $CDCl_3$.

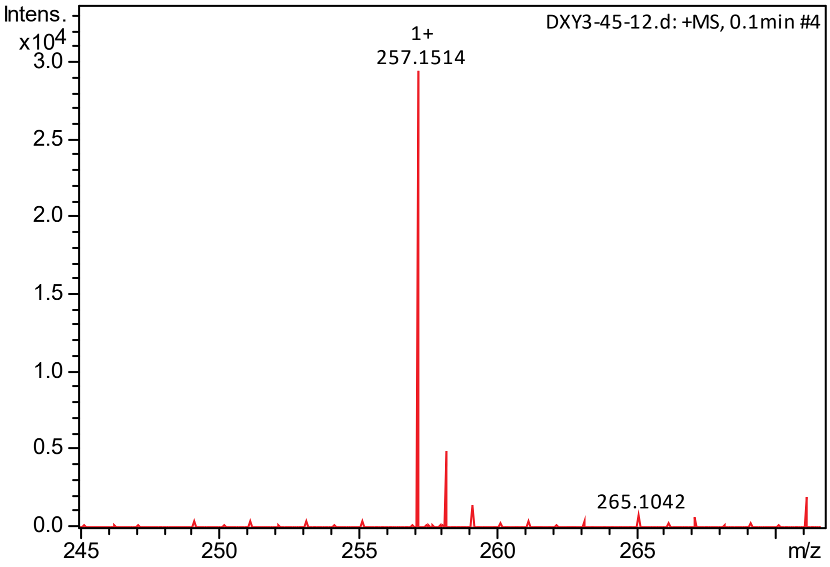

Figure S52. HRESIMS spectrum of aurantiophilane G (**6**) in $CDCl_3$.

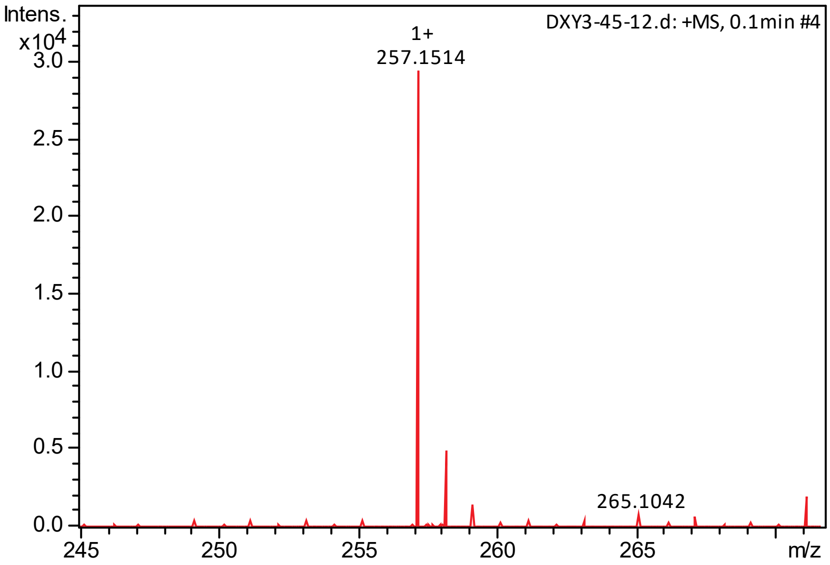

Figure S53. HRESIMS spectrum of aurantiophilane G (**6**) in $CDCl_3$.

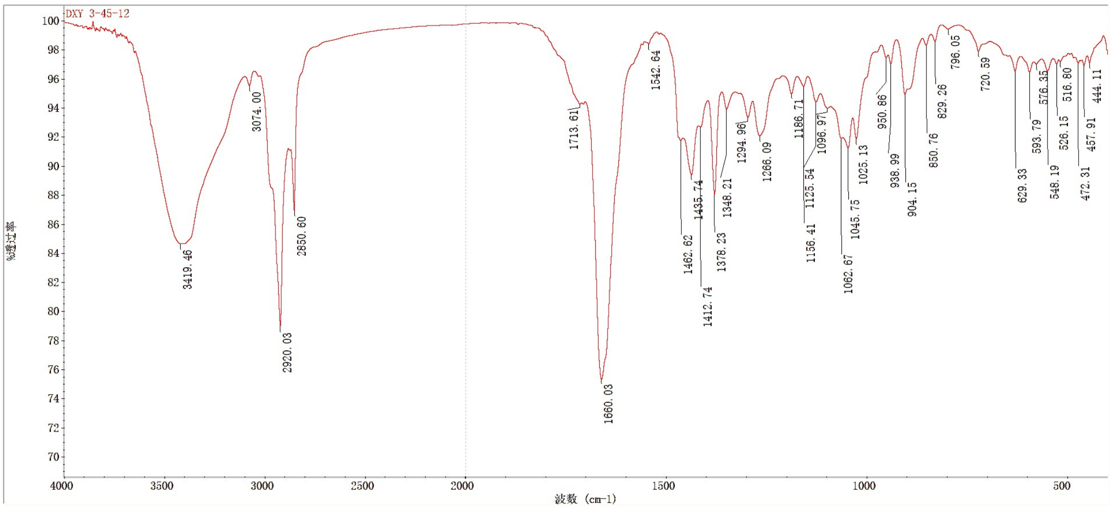

Figure S54. IR spectrum of aurantiophilane G (**6**) in $CDCl_3$.

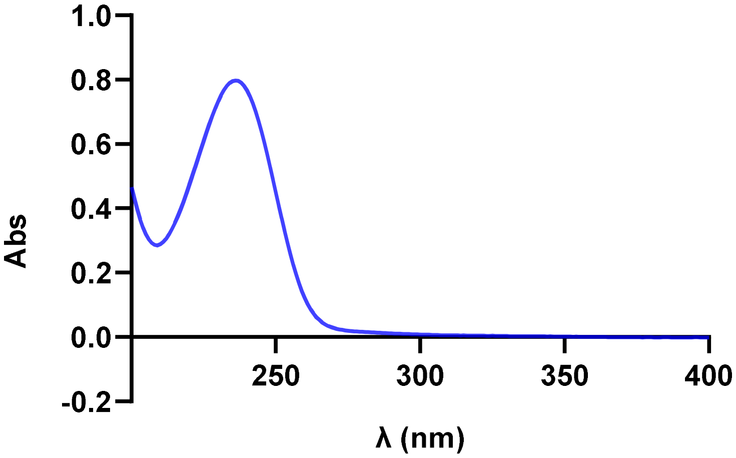

Figure S55. UV spectrum of aurantiophilane G (**6**) in MeCN.
